# Photoinduced in situ generation of DNA-targeting ligands: DNA-binding and DNA-photodamaging properties of benzo[*c*]quinolizinium ions

**DOI:** 10.3762/bjoc.20.11

**Published:** 2024-01-18

**Authors:** Julika Schlosser, Olga Fedorova, Yuri Fedorov, Heiko Ihmels

**Affiliations:** 1 Department of Chemistry and Biology, and Center of Micro- and Nanochemistry and (Bio)Technology (Cµ), University of Siegen, Adolf-Reichwein-Str. 2, D-57068 Siegen, Germany; 2 A. N. Nesmeyanov Institute of Organoelement Compounds, Russian Academy of Sciences, Vavilova str. 28, 119991 Moscow, Russia

**Keywords:** DNA intercalators, heterocycles, photocyclization, photosensitizer, styrylpyridines

## Abstract

The photoreactions of selected styrylpyridine derivatives to the corresponding benzo[*c*]quinolizinium ions are described. It is shown that these reactions are more efficient in aqueous solution (97–44%) than in organic solvents (78–20% in MeCN). The quinolizinium derivatives bind to DNA by intercalation with binding constants of 6–11 × 10^4^ M^−1^, as shown by photometric and fluorimetric titrations as well as by CD- and LD-spectroscopic analyses. These ligand–DNA complexes can also be established in situ upon irradiation of the styrylpyridines and formation of the intercalator directly in the presence of DNA. In addition to the DNA-binding properties, the tested benzo[*c*]quinolizinium derivatives also operate as photosensitizers, which induce DNA damage at relative low concentrations and short irradiation times, even under anaerobic conditions. Investigations of the mechanism of the DNA damage revealed the involvement of intermediate hydroxyl radicals and C-centered radicals. Under aerobic conditions, singlet oxygen only contributes to marginal extent to the DNA damage.

## Introduction

DNA intercalators – most often represented by small planar heteroaromatic compounds – play an important role as chemotherapeutic agents [1–4]. Specifically, upon intercalation into the DNA double helix such ligands can cause a change of the DNA structure or occupy binding sites of essential enzymes, which in turn may influence or even inhibit important biochemical processes, for example DNA replication or transcription [1–2]. As a result, the development of DNA-targeting drugs still involves the design of suitable DNA intercalators, and some currently applied anticancer drugs actually operate on the basis of intercalation [3]. Hence, several classes of compounds have been established, whose DNA-binding properties can be tailored and fine-tuned for that purpose, for example anthracyclines [5], indolocarbazoles [6], acridines [7], quinoxalines [8], naphthalimides [9], phenanthridines [10], cyanines [11], or indoles [12], as well as metal-organic complexes [13], and several others [1–2,4].

In this context, benzoquinolizinium derivatives and resembling polycyclic azoniahetarenes are an established class of DNA-binding compounds, which have been employed in biomedical imaging and as potential DNA-targeting anticancer agents [14–17]. More recently, a benzoquinolizinium-based fluorescent dye was reported to be used as imaging agent for inflammation and for the evaluation of the physiological response to anti-inflammatory drugs [18].

**Scheme 1:**
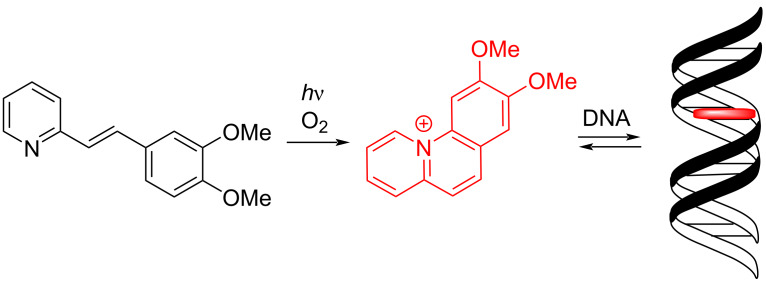
Photoinduced formation of benzo[*c*]quinolizinium and its interaction with DNA upon intercalation.

In this context, the benzo[*c*]quinolizinium structure provides some special features. First of all, it has the general requirements of a DNA intercalator, namely a planar, polycyclic heteroaromatic structure and a permanent positive charge [14]. Moreover, it has been shown that this DNA-binder and resembling intercalators can be directly generated upon irradiation of styrylazines under aerobic conditions, even in the presence of DNA, which provides local and temporal control of the DNA-binding event (Scheme 1) [14]. Specifically, the styrylpyridine, which does not bind to DNA, can be delivered without effect to the binding site, where the DNA-binding benzoquinolizinium ligand can then be generated as needed upon irradiation. Notably, the use of light for the activation of photo-controllable DNA ligands offers several advantages because it is easy to apply, traceless, and non-invasive [19]. As a result, several photoactive compounds have been developed, whose DNA-binding properties can be efficiently switched on and off by light [14,20–34].

In addition to their DNA-binding properties some annelated quinolizinium derivatives have also the ability to induce DNA damage upon irradiation [35–38], and may therefore be considered as promising basis for the development of new reagents for photodynamic (chemo)therapy (PDT). Notably, PDT has developed into an important therapeutic tool against several serious diseases, such as cancer [39], and bacterial, fungal, parasitic and viral infections [40–41]. In general, PDT operates on the basis of a photosensitizer, which generates reactive intermediates upon irradiation [42–45]. Hence, in the type-I mechanism the photosensitizer induces the formation of reactive oxygen species (ROS), such peroxyl, alkoxy and hydroxyl radicals, or carbon-centered radicals, which subsequently induce DNA strand cleavage. In the type-II mechanism, a triplet-excited photosensitizer reacts with molecular oxygen to give highly reactive singlet oxygen, ^1^O_2_, as reactive intermediate, which in turn oxidizes the DNA bases [46]. As a result, various classes of photosensitizers [47–49] have been established, for example, porphyrins [50], chlorins [51], phthalocyanines [52], porphycenes [53], metal-organic complexes [54–56], dye aggregates [57], as well as nano-drug carriers and metal-based nanoparticles [58–59]. But although these classes of compounds have been intensively studied and already contributed significantly to the field of PDT, there is still a demand for novel DNA-photodamaging ligands that could be applied for specific purposes, e.g., to improve efficacy or to limit side-effects. Therefore, the search for a class of photosensitizers is still a topical research area in photobiology [60]. To this end, benzo[*c*]quinolizinium derivatives may be considered as feasible photosensitizers because they can be formed readily in situ in the presence of DNA and because the structurally related alkaloids berberine [61–65] and coralyne [36,66–67] have been shown already to act as efficient photosensitizers for DNA damage. To the best of our knowledge, however, benzo[*c*]quinolizinium derivatives have not been investigated with respect to their DNA-photodamaging properties, so far. Therefore, we have synthesized selected benzo[*c*]quinolizinium derivatives and studied their DNA-binding and DNA-photodamaging properties.

## Results and Discussion

### Synthesis

**Scheme 2:**
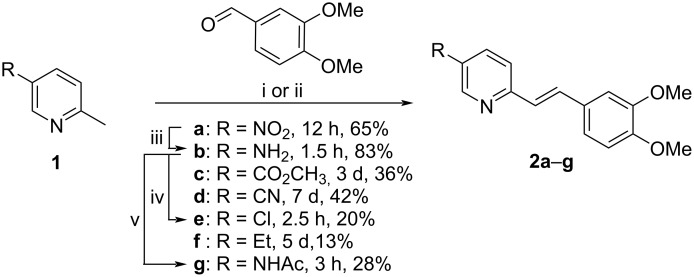
Synthesis of styrylpyridine derivatives **2a**–**g**. Conditions: i: piperidine, MeOH, reflux (**2a**,**c**), ii: Ca(OTf)_2_, Bu_4_NPF_6_, 130 °C (**2d**,**f**), iii: N_2_H_4_·H_2_O, Pd/C, MeOH, reflux (**2b**), iv: NaNO_2_, CuCl, aq HCl (37%), room temp., 2 h, 60 °C, 30 min (**2e**), v: acetyl chloride, pyridine, THF, room temp. (**2g**).

The styrylpyridine derivatives **2a**,**c**,**d**,**f** were synthesized by a piperidine- or Ca(OTf)_2_-catalyzed condensation reaction of 3,4-dimethoxybenzaldehyde with 5-substituted 2-picoline derivatives in low to moderate yields ranging from 13% (**2f**) to 65% (**2a**) (Scheme 2). The amino-substituted derivative **2b** was synthesized by reduction of the nitrostyrylpyridine **2a** with Pd/C and hydrazine in 83% yield. Subsequent acylation of the amine **2b** gave the corresponding amide **2g** in 28% yield. The chloro-substituted derivative **2e** was synthesized in a Sandmeyer-reaction from **2b** in 20% yield. The products **2a**–**g** were identified and fully characterized by NMR spectroscopy (^1^H, ^13^C, COSY, HSQC, and HMBC), elemental analyses, and mass spectrometry. In all cases, the *E*-configuration of the alkene double bonds was indicated by characteristic coupling constants of the alkene protons (^3^*J*_H–H_ = 16 Hz).

### Absorption properties and photoreactions of styrylpyridine derivatives

**Figure 1:**
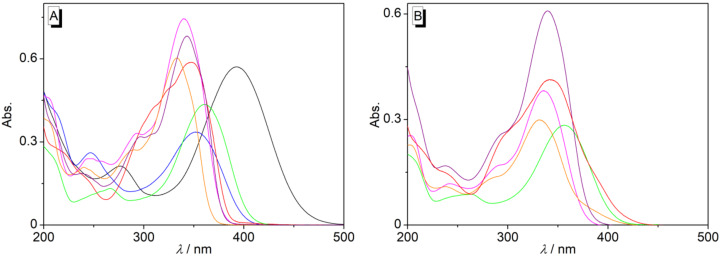
Absorption spectra of styrylpyridine derivatives **2a** (black), **2b** (red), **2c** (blue), **2d** (green), **2e** (magenta), **2f** (orange), and **2g** (purple) in MeCN (A) and H_2_O (B) (*c* = 20 µM).

In acetonitrile solution, the styrylpyridines **2a**–**g** exhibited long-wavelength absorption bands with maxima in a range from λ_max_ = 333 nm for the ethyl-substituted compound **2f** to λ_max_ = 394 nm for the nitro-substituted derivative **2a** (Figure 1A, Supporting Information File 1, Table S1). As compared with the absorption maximum of the parent compound at λ_max_ = 330 nm [20], the derivatives **2b**–**g** showed a slight bathochromic shift mostly in the range of λ_max_ = 333–360 nm, whereas for the nitro-substituted compound **2a** a stronger red shift of the absorption maximum was observed, presumably caused by the strong electron-withdrawing property of the nitro group resulting in a more pronounced intramolecular charge transfer [68]. In water, the absorption maxima showed only a small shift of the absorption maxima of 1–4 nm as compared with the ones in acetonitrile (Figure 1B, Supporting Information File 1, Table S1). The absorption spectra of compounds **2a** and **2c** could not be recorded because of their low water solubility.

**Figure 2:**
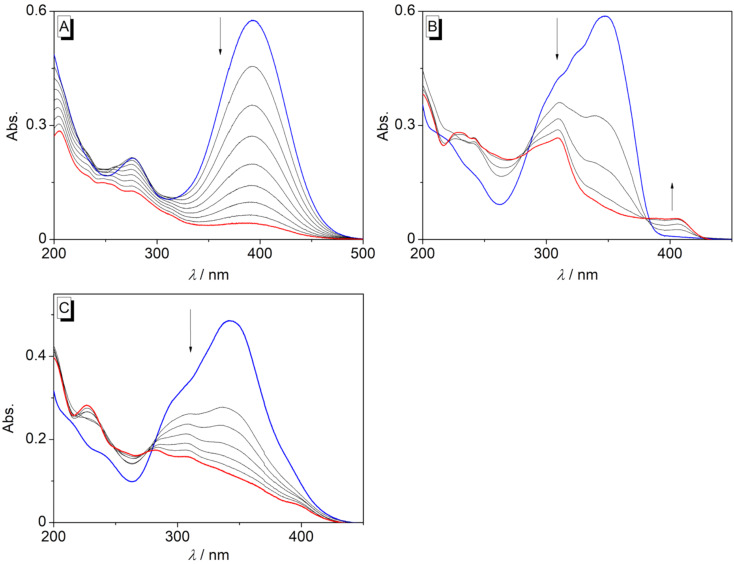
Changes of the absorption spectra during the irradiation of **2a** in MeCN for 16 min (A), **2b** in MeCN for 4 min (B), and **2b** in H_2_O/MeCN 49:1 for 6 min (C) (*c* = 20 µM, λ_ex_ > 220 nm, irradiated in a cuvette). Blue: spectrum of the starting material before irradiation; red: spectrum at the end of the irradiation.

The styrylpyridine derivatives **2a**–**g** were irradiated in oxygen-saturated solutions in MeCN, H_2_O, MeOH, or MeCN/H_2_O with a high-pressure Hg lamp (λ > 220 nm), and the course of the photocyclization reaction was monitored by absorption spectroscopy (Figure 2). In general, the absorption maximum of the derivatives **2b**–**g** decreased during the photoreaction with formation of new red-shifted absorption bands. Nevertheless, the new red-shifted absorption band of the amino-substituted styryl derivative **2b** in MeCN was weak and very broad, indicating only negligible formation of the photocyclization product (Figure 2B). Likewise, in H_2_O solution with MeCN as co-solvent, no formation of a new absorption band was observed upon irradiation of **2b**, either (Figure 2C). Moreover, the aminobenzoquinolizinium **3b** could not be isolated after irradiation of **2b** at larger scale. As an exception, the irradiation of the nitro-substituted styrylpyridine derivative **2a** in MeCN or H_2_O led to disappearance of the long-wavelength absorption maximum with no formation of a distinct new band (Figure 2A), which usually indicates photoinduced decomposition. This observation is in agreement with reports on resembling aromatic *p*-nitro-substituted derivatives, which do not react in a photocyclization reaction [69].

**Figure 3:**
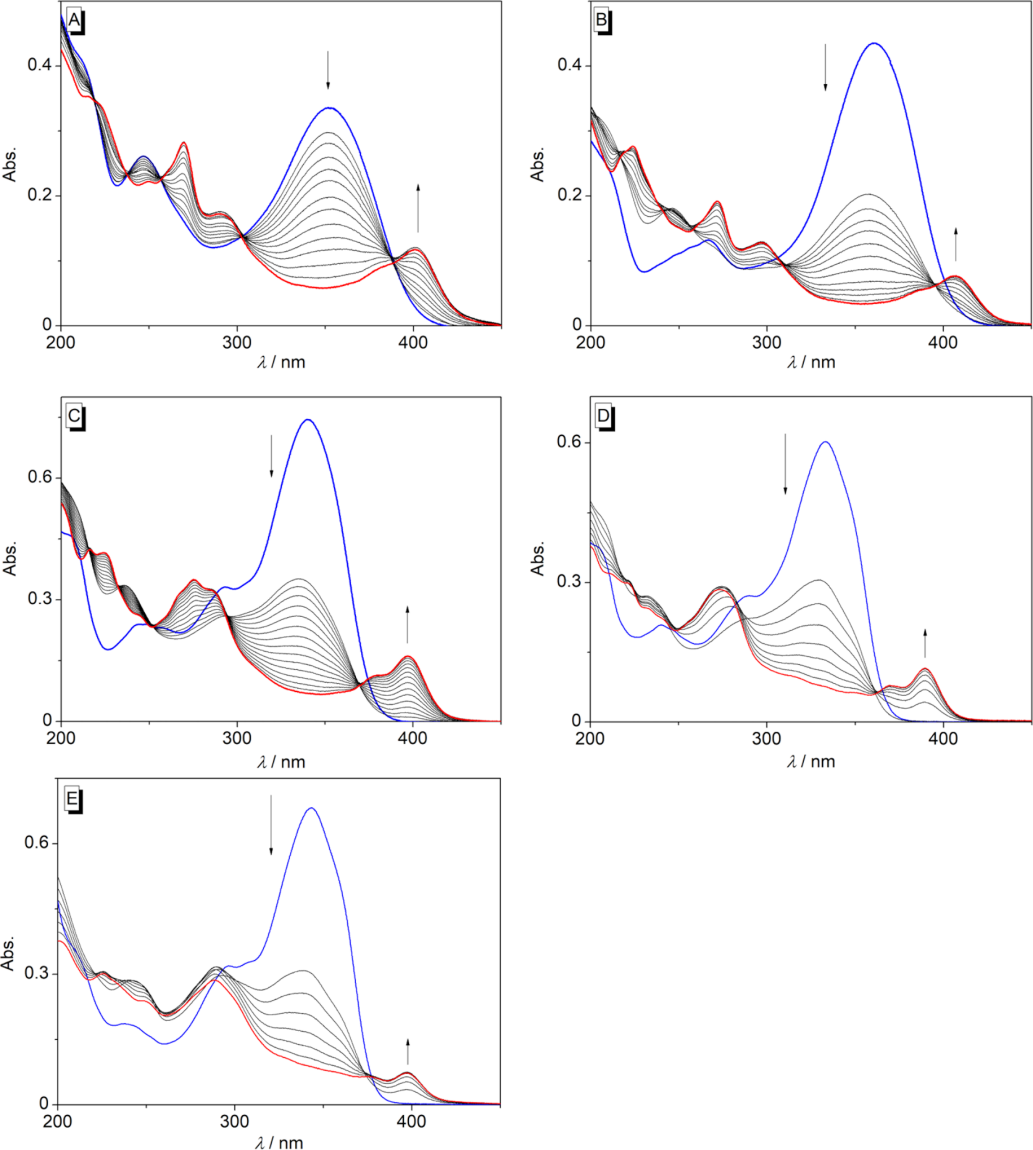
Changes of the absorption spectra during the irradiation of **2c** for 13 min (A), **2d** for 12 min (B), **2e** for 15 min (C), **2f** for 10 min (D), and **2g** for 7 min (E) (in MeCN, *c* = 20 µM, λ_ex_ > 220 nm, irradiated in a cuvette). Blue: spectrum of the starting material before irradiation; red: spectrum at the end of the irradiation.

Direct irradiation of the styrylpyridine derivatives **2c**–**g** in MeCN led to the formation of new absorption bands in the range from λ = 389 nm (**2f**) to λ = 407 nm (**2d**) with bathochromic shifts of Δλ = 47–57 nm (Figure 3, Supporting Information File 1, Table S1), which indicated the formation of the benzo[*c*]quinolizinium ions **3c**–**g** as photocyclization products [20]. The absence of isosbestic points during the photometric monitoring of the photoreactions indicated a stepwise formation of different intermediates in the reaction sequence starting with *E*–*Z* isomerization, followed by photocyclization and subsequent oxidation.

**Scheme 3:**
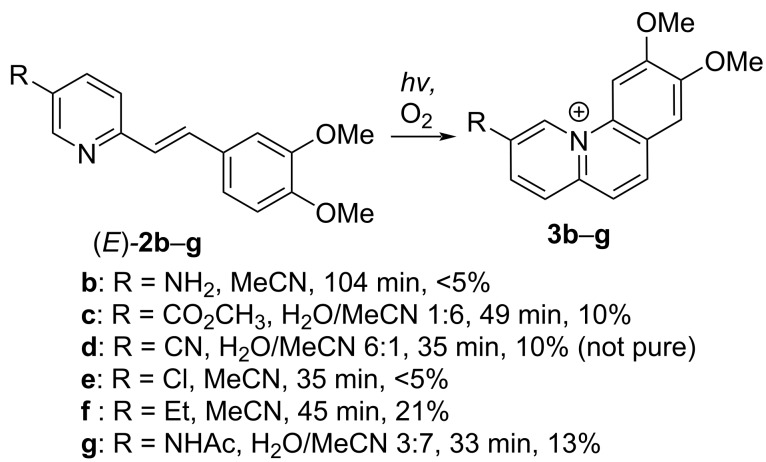
Photoinduced formation of styrylpyridine derivatives **2b**–**g** to the benzo[*c*]quinolizinium ions **3b**–**g** (yields in % refer to isolated products).

In general, the photoreaction was more efficient in polar, protic aqueous solvents (cf. Supporting Information File 1, Figures S2B–S6B) or in buffer solution (cf. Supporting Information File 1, Figures S3C–S6C) than in polar, aprotic MeCN solution. In MeOH the photoreactions were inefficient as indicated by the lack of red-shifted bands or by formation of broad, weak absorption bands (cf. Supporting Information File 1, Figures S1, S2A, S3A, S4A, S5A, and S6A). Because of the low water solubility of the styrylpyridine derivatives **2a**–**g**, solvent mixtures of MeCN/H_2_O or pure MeCN were used for the preparative photocyclization reactions (Scheme 3, cf. Supporting Information File 1, Figure S2C,D) to provide sufficient solubility of the substrates, as well as optimal efficiencies for the formation of photocyclization products. But even under these optimized conditions, compounds **3c**–**g** could be isolated only in low yields of <5–21%. Hence, to assess whether the products are generally formed to minor extent in the photoreaction or whether the low yields of isolated product result from significant losses during the purification process, the content and yield of the benzo[*c*]quinolizinium ions were determined directly after irradiation of **2c**, **2e**–**g** by photometric analysis of the reaction mixture (Figure 3, red spectra). The yield of the product **3d** could not be determined because it was not available in pure form on preparative scale. With the absorption data, specifically the molar extinction coefficients, obtained from the isolated benzo[*c*]quinolizinium derivatives, the yields of the initially formed photoproducts **3c**, **3e**, **3f**, and **3g** in the reaction mixtures were determined to be 78%, 73%, 30%, and 20% in MeCN, >97%, 80%, 44% and 55% in H_2_O, and 32%, 76%, 53% and 38% in Na phosphate buffer. Overall, the initially formed amounts of photoproducts are significantly larger than the ones of the isolated compounds, in the case of **3c** and **3e** even with good yields, so that it may be concluded that the small amounts of isolated product result from losses during work-up and purification.

### DNA-binding properties of benzoquinolizinium derivatives **3c**,**e–g**

**Figure 4:**
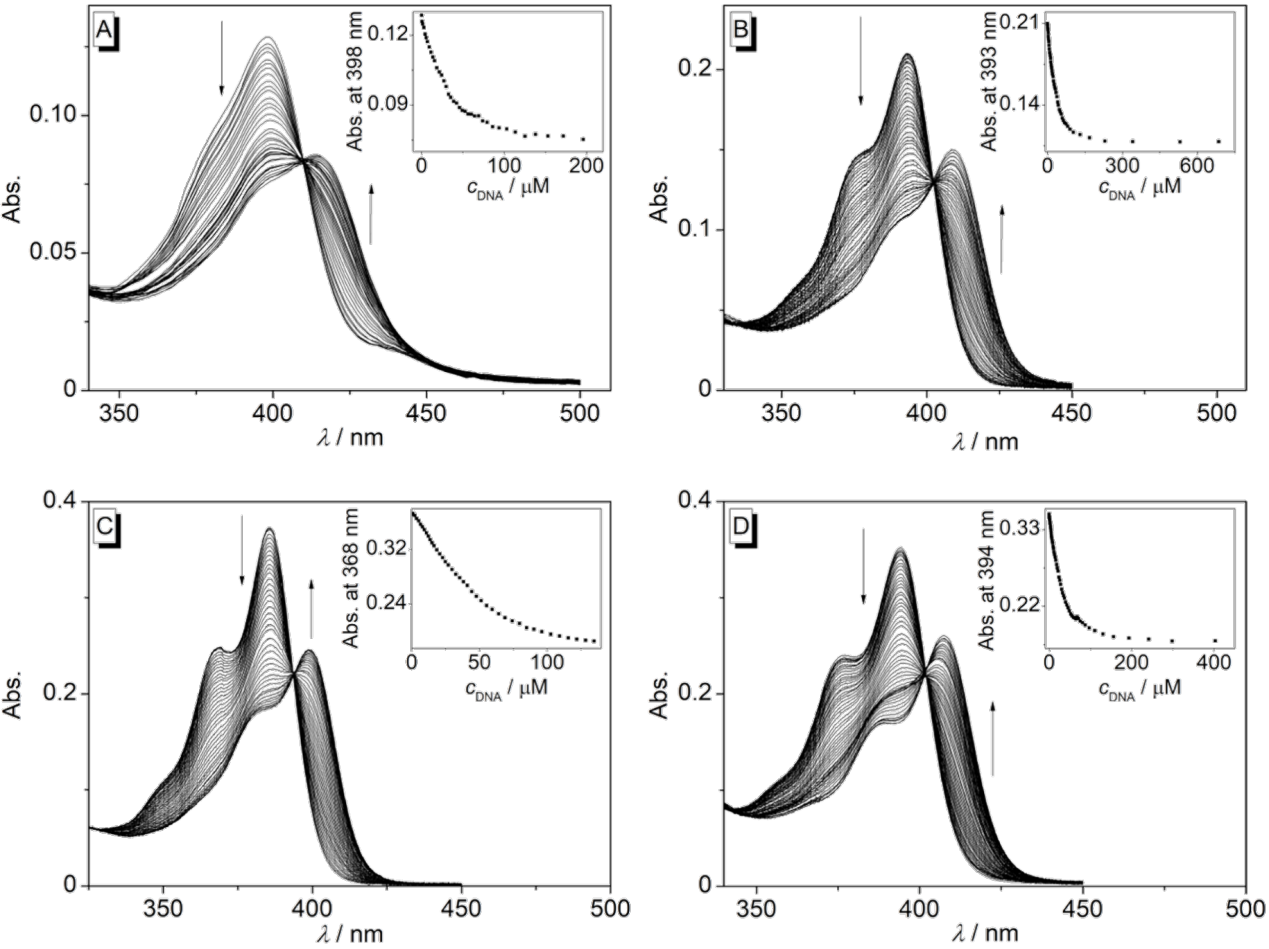
Photometric titration of ct DNA to **3c** (A) **3e** (B) **3f** (C) and **3g** (D) (*c* = 20 µM) in Na phosphate buffer (pH 7.0, *T* = 20 °C, *c*_Na+_ = 16 mM). The arrows indicate the development of the absorption bands during titration. Inset: plot of absorption versus *c*_DNA_.

**Figure 5:**
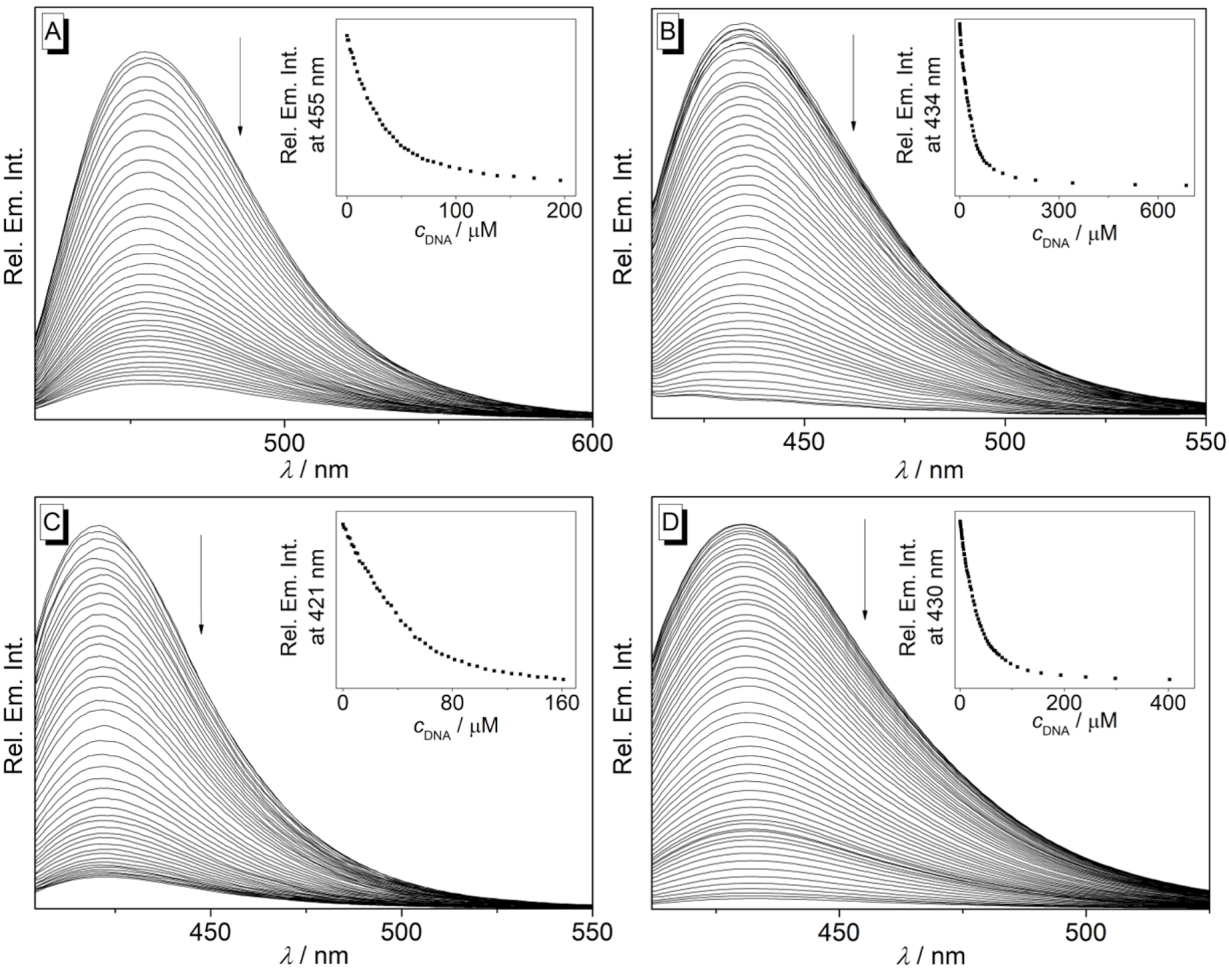
Fluorimetric titration of ct DNA to **3c** (A), **3e** (B), **3f** (C), and **3g** (D) (*c* = 20 µM) in Na phosphate buffer (pH 7.0, *T* = 20 °C, *c*_Na+_ = 16 mM). The arrows indicate the development of the emission bands during titration. Inset: plot of relative emission intensity versus *c*_DNA_.

The styrylpyridines **2d**–**g** and the benzo[*c*]quinolizinium derivatives **3c**,**e**–**g** were investigated with respect to their DNA-binding properties with calf thymus (ct) DNA. The titrations of ct DNA to compounds **2d**–**g** resulted in no or only negligible changes of the absorption and fluorescence spectra (cf. Supporting Information File 1, Figures S8–S11), indicating that these substrates do not interact significantly with DNA. In contrast, upon addition of DNA to compounds **3c**,**e**–**g**, the absorption maxima at 398 nm, 393 nm, 386 nm and 394 nm were red-shifted with a hypochromic effect, and isosbestic points developed during all titrations (Figure 4, Table 1). Furthermore, the addition of DNA to substrates **3c**,**e**–**g** led to efficient fluorescence quenching (Figure 5), which is commonly observed with this class of cationic ligands [3,70], mainly as a result of a photoinduced electron transfer from the excited, DNA-bound ligand with the DNA bases [71]. The binding isotherms obtained from the titration data were used to determine the binding constants, *K*_b_, of the DNA ligands. Thus, the derivatives **3c**, **3e**, and **3g** bind to ct DNA with *K*_b_ values of 6.0 × 10^4^ M^−1^, 5.9 × 10^4^ M^–1^, and 6.4 × 10^4^ M^−1^, respectively, whereas the affinity of ligand **3f** is slightly higher with *K*_b_ = 1.1 × 10^5^ M^−1^, which is in the same range as the binding constant reported for the 8,9-dimethoxybenzo[*c*]quinolizinium (*K*_b_ = 1.2 × 10^5^ M^−1^) [20]. The slightly lower binding constants of derivatives **3c**, **3e**, and **3g** as compared with the one of compound **3f**, may be explained by the larger substituents of the former ligands, which cause more steric repulsion within the binding site.

**Table 1:** Absorption and emission maxima of **3c**–**g** in the absence and presence of ct DNA and binding constants of their complexes with ct DNA.

	λ_abs_ / nm^a^		Δλ / nm	*K*_b_ / 10^4^ M^−1b^
	
	without DNA	with DNA		

**3c**	398	415	17	6.0
**3d**	–	–	–	–
**3e**	393	405	12	5.9
**3f**	386	398	22	11
**3g**	394	409	15	6.4

^a^In Na phosphate buffer, *c*_Na+_ = 16 mM, pH 7.0, 20 °C. ^b^Determined from photometric titrations.

**Figure 6:**
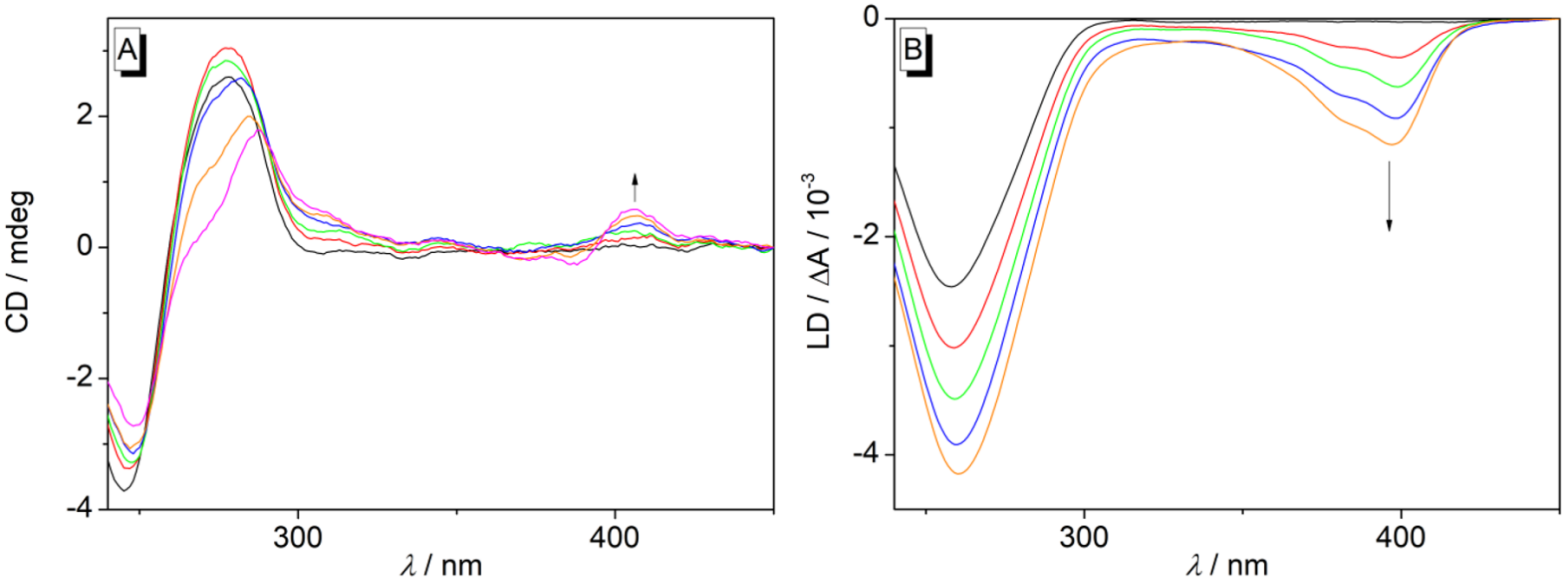
CD (A) and LD (B) spectra of **3f** and ct DNA (*c*_DNA_ = 20 µM) in Na phosphate buffer (pH 7.0, *T* = 20 °C, *c*_Na+_ = 16 mM) at LDR = 0 (black), 0.2 (red), 0.5 (green), 1.0 (blue), 1.5 (orange), and 2.0 (magenta).

The binding mode of the benzo[*c*]quinolizinium derivatives **3c**,**e**–**g** with DNA was further examined with circular dichroism (CD) and linear dichroism (LD) spectroscopy (Figure 6 and Supporting Information File 1, Figures S12–S14). Hence, with increasing ligand-DNA ratio (LDR) weak positive induced CD (ICD) signals developed in the long-wavelength absorption range of ligands **3e**–**g**, that is, where the DNA bases do not absorb (Figure 6A and Supporting Information File 1, Figures S13A and S14A), which is a commonly observed indication of DNA binding as the ICD bands result from non-degenerative coupling of the dipole moments of the ligand with the DNA bases [72]. The association of the ligands **3e**–**g** with DNA was further confirmed by the formation of negative LD bands with increasing *LDR* developing in the absorption range of the ligands (λ > 300 nm) (Figure 6B and Supporting Information File 1, Figures S13B and S14B), which is a characteristic indication of a coplanar alignment of the ligand relative to the base pairs in an intercalative binding mode [73]. In contrast, ligand **3c** did not exhibit an ICD signal in the presence of DNA (Supporting Information File 1, Figure S12A) and gave rather weak and less structured LD bands (Figure S12B) as compared with the ones of ligands **3e**–**g**. The lack of distinct ICD bands of DNA-bound **3c** might be explained by the very weak signals, which are usually observed for this class of DNA binder because of unfavorable angles between transition moments of ligand and DNA bases [72].

To gain additional information about the orientation of the benzo[*c*]quinolizinium ligand in the intercalation site, the reduced LD (LD*_r_*) spectrum was determined exemplarily for ligand **3f** (Supporting Information File 1, Figure S15) [74–75]. The analysis of the data revealed a binding angle α = 59° between the ligand **3f** and the DNA helix, thus indicating a tilted orientation of the ligand relative to the DNA base pairs within the binding site.

**Figure 7:**
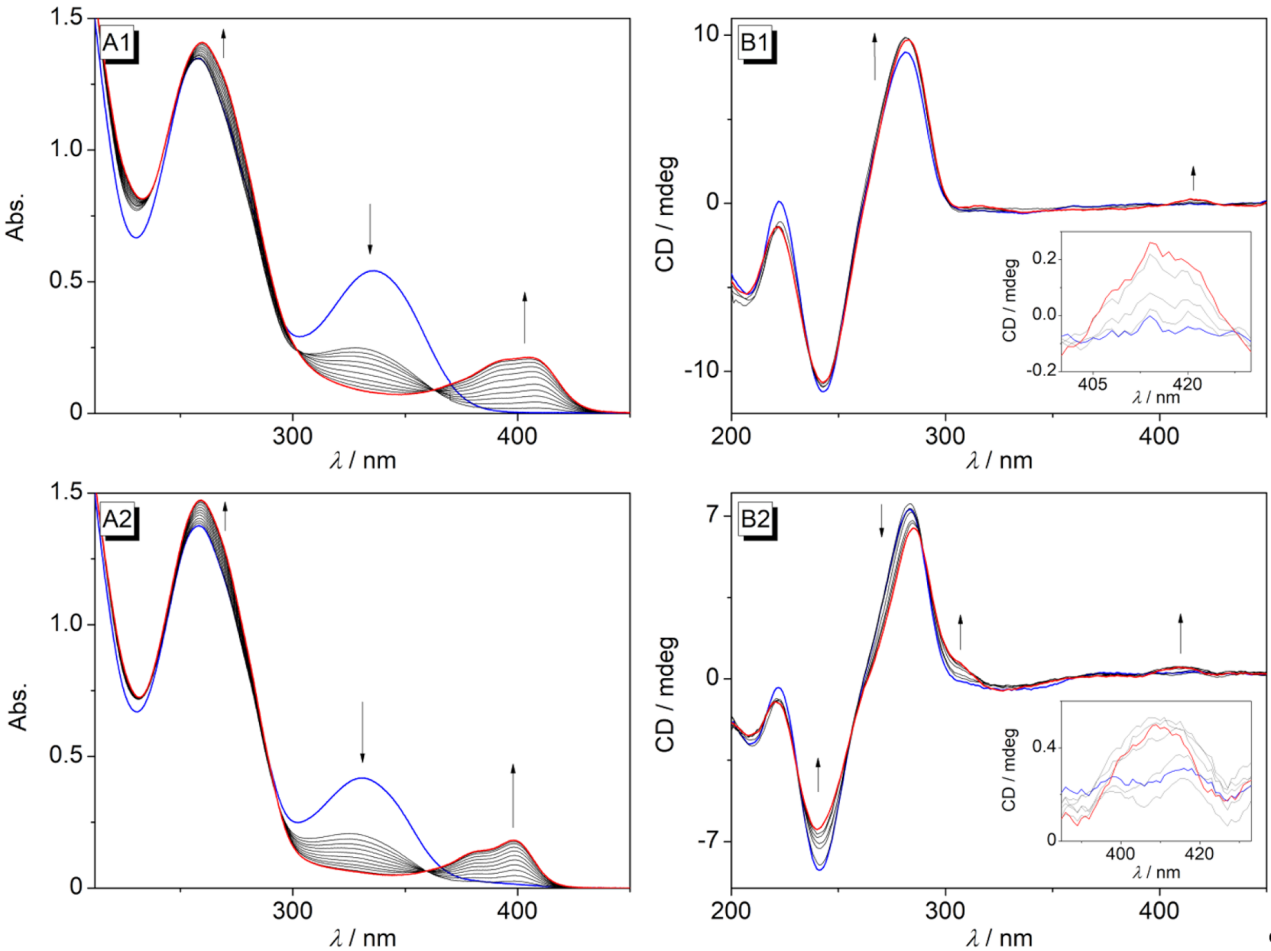
Changes of the absorption (A) and CD (B) spectra during the irradiation of **2e** (1) and **2f** (2) (*c* = 20 μM) in the presence of ct DNA (*c*_DNA_ = 0.1 mM) in Na phosphate buffer, *c*_Na+_ = 16 mM. Blue: spectrum of the starting material before irradiation; red: spectrum at the end of the irradiation.

The DNA-binding ligands were also generated in situ in the presence of DNA. For that purpose, solutions of the styrylpyridines **2d**–**g** and ct DNA were irradiated in phosphate buffer, and the formation of the dimethoxybenzo[*c*]quinolizinium ions and their subsequent binding to the DNA were shown photometrically by the development of the characteristic red-shifted absorption bands (Figure 7A, cf. Supporting Information File 1, Figure S7A), which matched the ones of the independently formed complexes of these ligands with DNA (see above). In the case of **3e**–**g**, the binding event was also confirmed by CD spectroscopy, namely by the formation of weak ICD signals (Figure 7B, cf. Supporting Information File 1, Figure S7B). Because of the very low solubility of compound **2c** in aqueous solutions, the former could not be irradiated in situ in the presence of DNA.

### Photoinduced DNA damage

In first orienting experiments, the photoinduced DNA damage by benzo[*c*]quinolizinium derivatives was examined exemplarily with ligand **3f** and plasmid DNA pBR322 (Table 2). To assess the optimal parameters for the photoreaction, the ligand was irradiated in the presence of the DNA under anaerobic conditions at different irradiation times and concentrations of the ligand. The DNA-strand cleavage was analyzed by agarose-gel electrophoresis. In this assay, the DNA-strand cleavage in the supercoiled plasmid DNA pBR322 is indicated by the formation of the relaxed, open-circular form [76]. It has to be noted that under these conditions the DNA is already damaged in the absence of the photosensitizer. Therefore, each series of experiments is complemented for comparison with control experiments without photosensitizer. In general, a photoinduced DNA damage by the quinolizinium **3f** was observed, whose extent increased with increasing irradiation time and with increasing concentration of **3f**. Thus, after 2 min of irradiation with fixed concentration (*c* = 2.5 × 10^−5^ M) 38% of the supercoiled DNA were transformed to the open-circular form, whereas after 5 min, 73% of the DNA were damaged by strand cleavage (Table 2A). At the same time, experiments with varying ligand concentration revealed 81% of damaged DNA after 2 min of irradiation with *c* = 5.0 × 10^−5^ M, which was almost twice as much as the cleavage (41%) determined with *c* = 2.5 × 10^−5^ M Table 2B). In addition, a small series of benzo[*c*]quinolizinium ligands **3c**,**e**–**g** was tested under conditions optimized for derivative **3f**. Within this series, the amount of DNA cleavage ranged fom 39% (**3c**) to 51% (**3e**) after 2 min of irradiation (Table 2C).

**Table 2:** Gel-electrophoretic analysis of photoinduced DNA-strand cleavage in the presence of **3f** depending on the irradiation time (A), on the concentration (B), and in the presence of **3c**,**e**–**g** (C). Lanes 1, 10 and 11 (A) and lane 11 (B): control experiment without **3f**. Lanes 1 and 2 (B): control experiment without **3f**, irradiated for 2 min. In all cases: *c***_3_** = 2.5 × 10^−5^ M, *c*_DNA_ = 3.5 × 10^−9^ M, anaerobic conditions, irradiation time: 2 min, λ_max_ = 366 nm.

A											
supercoiled open-circular	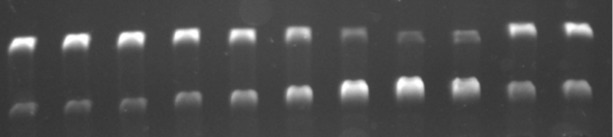										

lane	1	2	3	4	5	6	7	8	9	10	11

irradiation time / min	0	0	0	0.5	1	2	5	10	15	15	15
Strand cleavage / %	11	9	9	16	25	38	73	83	73	24	26

B											
	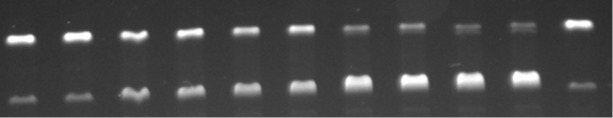										

lane	1	2	3	4	5	6	7	8	9	10	11

*c* / 10^−5^ M	0	0	1.3	1.3	2.5	2.5	3.8	3.8	5.0	5.0	0
strand cleavage / %	13	11	31	32	41	41	71	73	81	81	13

C											
	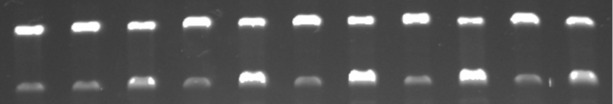										

lane	1	2	3	4	5	6	7	8	9	10	11

irradiation time / min	0	2	2	0	2	0	2	0	2	0	2
substrate	−	−	ref	**3f**	**3f**	**3g**	**3g**	**3e**	**3e**	**3c**	**3c**
strand cleavage / %	14	15	31	15	42	15	46	16	51	17	39

To investigate the mechanism of the DNA cleavage, commonly employed control experiments [46,77] were performed exemplarily with ligand **3f** (Table 3 and Supporting Information File 1, Figure S16). To assess the influence of oxygen, the DNA–ligand mixture was irradiated for 2 min under ambient aerobic conditions as well as in argon- and oxygen-saturated medium (Supporting Information File 1, Figure S16A). Under anaerobic atmosphere, 50% of the supercoiled DNA were transformed to open-circular DNA after 2 min of irradiation, whereas under atmospheric conditions and oxygen-saturation only 36% and 30% of the DNA were cleaved, respectively. However, with prolonged irradiation time, even under oxygen atmosphere the photocleavage activity increased to give 65% strand cleavage after 5 min and 85% after 10 min of irradiation (Supporting Information File 1, Figure S16B).

**Table 3:** Photoinduced DNA-strand cleavage by benzoquinolizinium **3f** under different conditions.^a^

Additive	*c*_Additive_	Atmosphere	Strand cleavage / %^b^

–		anaerobic^c^	50
DMSO	5 vol %	anaerobic^c^	34
*t-*BuOH	5 vol %	anaerobic^c^	28
2-PrOH	5 vol %	anaerobic^c^	23
TEMPO^d^	1.3 × 10^−2^ M	anaerobic^c^	13
2-mercaptoethanol	2.0 × 10^−2^ M	anaerobic^c^	18
2-mercaptoethylamine·HCl	2.0 × 10^−2^ M	anaerobic^c^	16
–		aerobic	36
–		O_2_ saturated	30
–		O_2_ saturated^e^	65
–		O_2_ saturated^f^	85
NaN_3_	2.5 × 10^−4^ M	aerobic	25
D_2_O (solvent)		aerobic	23

^a^In all cases: *c***_3f_** = 2.5 × 10^–5^ M, *c*_DNA_ = 3.5 × 10^–9^ M (pBr322), irradiation time = 2 min; λ_ex_ = 366 nm. ^b^Determined by agarose gel electrophoresis and staining with ethidium bromide. ^c^Ar saturated. ^d^TEMPO = 2,2,6,6-tetramethylpiperidine 1-oxyl. ^e^Irradiation time = 5 min. ^f^Irradiation time = 10 min.

To clarify whether the mechanism of the DNA photodamage proceeds through the formation of radicals, experiments with commmonly employed radical scavengers were conducted (Table 3, Supporting Information File 1, Figure S17). In the presence of hydroxyl-radical scavengers DMSO, *t*-BuOH, and 2-propanol [78] the cleavage of the DNA was reduced to 34%, 28%, and 23%, respectively, which may indicate that the cleavage of DNA involves hydroxyl radicals (Supporting Information File 1, Figure S17A). At the other hand, a clear decrease of DNA damage to ca. 13%, 18% and 16% in the presence of 2,2,6,6-tetramethylpiperidine 1-oxyl (TEMPO), 2-mercaptoethanol and 2-mercaptoethylamine hydrochloride, respectively, showed that C-centered radicals contribute even more to the DNA damage than hydroxyl radicals (Supporting Information File 1, Figure S17B). It should be noted, however, that these scavengers may also intercept hydroxyl radicals [79] or interfere with the DNA damage by alternative pathways [80]. In any case, the significant decrease of DNA damage in the presence of the radical scavengers indicated the formation and direct or indirect participation of carbon radicals and hydroxyl radicals in the photoinduced DNA damage.

In order to investigate the involvement of singlet oxygen in the DNA cleavage process, the samples were irradiated in the presence of NaN_3_ or in D_2_O (Table 3, Supporting Information File 1, Figure S18). The latter is known to extend the lifetime of singlet oxygen by a factor of ca. 10 as compared with H_2_O. Therefore, strand cleavage reactions induced by singlet oxygen are more efficient in D_2_O than in H_2_O [81]. However, irradiation of **3f** in D_2_O resulted in essentially the same DNA cleavage (23%) as compared with the reaction in H_2_O (25%) under otherwise identical conditions (Supporting Information File 1, Figure S18A). In the presence of NaN_3_ (2.5 × 10^−5^ M), which is a known radical scavenger for singlet oxygen [82], a strand cleavage of 25% occurred, whereas 38% cleavage was observed in the absence of NaN_3_. Nevertheless, a larger access of the scavenger (2.5 × 10^−4^ M) resulted in a decrease of the cleavage to 17%. It has to be noted, however, that an inhibition of DNA cleavage in the presence of NaN_3_ may also be induced by direct deactivation of the excited photosensitizer by the azide and not only from quenching of singlet oxygen [83]. Similar, seemingly contradictory effects of D_2_O and NaN_3_ on the photoinduced DNA cleavage were observed with other photosensitizers [84–85]. In addition, it has been reported that a relative large excess of NaN_3_ is required to detect an efficient inhibition of DNA-photocleavage [86]. Overall, these results as well as the efficient photocleavage under oxygen-saturated conditions, at least with long irradiation times (Supporting Information File 1, Figure S18B), indicated the direct or indirect involvement of singlet oxygen in the overall mechanism; yet, only to a small extent.

**Scheme 4:**
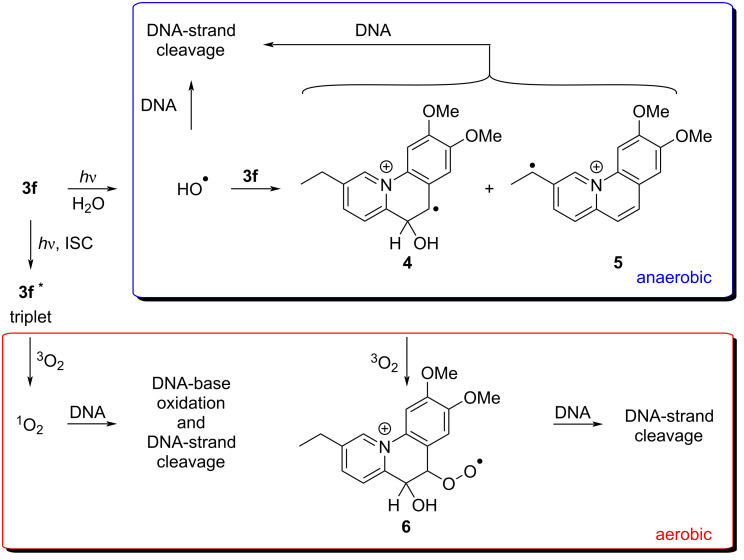
Proposed mechanisms for the photoinduced DNA damage initiated by photoexcitation of benzoquinolizinium **3f**.

Overall, the experiments on the photoinduced DNA damage by benzo[*c*]quinolizinium **3f** revealed a more complex mechanistic scenario (Scheme 4). While it became clear that the irradiation of this substrate in the presence of DNA leads to efficient DNA-strand cleavage, the systematic assessment of parameters that influence this reaction revealed the formation of different reactive intermediates (Scheme 4). Under anaerobic conditions, the DNA damage is similar to the one observed with the isomeric benzo[*c*]quinolizinium ions [35]. In the latter case, it has been shown that frank DNA-strand breaks are induced by hydroxyl radicals, supposedly formed by photoinduced electron transfer (PET) reaction of the strongly oxidizing excited quinolizinium ion. Likewise, the results obtained with **3f** point to the formation of hydoxyl radicals that are known to induce DNA-strand breaks. At the same time, the formation of C-centered radicals was indicated by the pronounced decrease of photocleavage in the presence of the corresponding radical scavengers. As there is no obvious reaction mechanism for the direct formation of C-radicals upon irradiation of **3f** it is proposed that the reaction of the initially formed hydroxyl radicals with the benzoquinolizinium **3f** leads to the formation of the C-centered radicals **4** and **5**, namely by addition of the radical or by hydrogen abstraction at the methylene group of the ethyl substituent (Scheme 4). Subsequently, the intermediate radicals **4** and **5** induce DNA-strand breaks initiated by hydrogen abstraction reactions at the ribose residues [78,87].

Most notably, under aerobic conditions, a reduced DNA photocleavage was observed as compared with the reaction under anaerobic conditions. This result is somewhat surprising as the formation of singlet oxygen, ^1^O_2_, by the reaction of the triplet-excited **3f** and oxygen was confirmed in control experiments. And the reactive intermediate ^1^O_2_ is known to induce DNA damage [78,88]. Nevertheless, these lesions, namely DNA base oxidations, often require alkaline treatment to lead to a strand cleavage, so that the DNA damage remained mainly unnoticed in the employed assay. At the same time, it has been reported that ^1^O_2_ can also efficiently induce single-strand breaks directly [89], so that the observed low DNA cleavage under aerobic conditions may be assigned to such a reaction. Moreover, it should be noted that both ^3^O_2_ and ^1^O_2_ [46] might react with other intermediates formed during the photoreaction, for example by the reaction with C-radicals **4** and **5** to give peroxides such as **6** (Scheme 4), by cycloaddition of ^1^O_2_ to alkene and diene units, or by deactivation of the excited state in a triplet-triplet annihilation [90], all of which leading to a reduced photocleavage efficiency. However, with much longer irradiation time under oxygen-saturated conditions, a more efficient photodamage was observed, which may be induced by those intermediates or secondary products formed from these ^1^O_2_ reactions.

In comparison with the already known quinolizinium-type photosensitizers [35,91], the photoinduced DNA damage by benzo[*c*]quinolizinium derivatives is more efficient under resembling conditions. Thus, the isomeric benzo[*b*]quinolizinium cations showed DNA damage to a lower extent after irradiation for 10 min with 15–20% and 20–25% DNA cleavage under anaerobic and aerobic conditions, respectively [35]. Likewise, naphthoquinolizinium salts exhibited DNA cleavage of about 20% and 50% after 5 min and 10 min, respectively, under anaerobic conditions, thus showing lower efficiencies as compared with photosensitizer **3f** with DNA cleavage of 73% and 83%, respectively [91].

## Conclusion

In summary, it was demonstrated that the photoinduced cyclization reaction of readily available styrylpyridine derivatives **2a**–**g** gives the corresponding benzo[*c*]quinolizinium derivatives and that these reactions are more efficient in aqueous solutions than in organic solvents. The benzo[*c*]quinolizinium derivatives have the typical properties of DNA intercalators and bind to DNA with *K*_b_ values of 6.0–11 × 10^4^ M^–1^. Importantly, the ligand–DNA complex may be accessed as needed in situ upon irradiation of the styrylpyridines **2c**,**e**–**g** in the presence of DNA, which is a useful feature of DNA-targeting substrates, specifically for a spatio-temporal control of this biological activity.

Furthermore, we have discovered that this class of compounds has a large potential to operate as photosensitizer that induces DNA damage already at relatively low irradiation times and low concentrations. Most notably, the photoinduced DNA damage does not necessarily require oxygen, unlike type-II photosensitizers. In fact, the representative compound **3f** is a more efficient DNA-damaging photosensitizer under anaerobic conditions, which may be an advantage for applications in hypoxic cancer cells. Preliminary investigations of the mechanism of the DNA damage revealed the involvement of intermediate hydroxyl radicals and C-centered radicals. Singlet oxygen, one of the most important reactive intermediates in conventional PDT, however, only contributes to marginal extent to the DNA damage. Therefore, these results are a promising starting point for the development of novel photosensitizers based on benzo[*c*]quinolizinium derivatives because their particular mode of activity may offer complementary applications in addition to the already established photosensitizers.

Overall, the benzo[*c*]quinolizinium scaffold offers some advantageous properties for its use as DNA-targeting agent, both as photo-controllable DNA binder and as DNA-damaging photosensitizer. Still, some key parameters have to be optimized by variation of the substitution pattern. For example, the water solubility of the styrylpyridine substrates has to be increased, and the excitation wavelength for the photocyclization reaction has to be red-shifted. But with a focus on the improvement of these properties the benzo[*c*]quinolizinium ion should be considered as a promising platform for further development of DNA-binding and DNA-photodamaging reagents.

## Experimental

### General methods

The commercially available chemicals (Alfa, Merck, Fluorochem or BLDpharm) were of reagent grade and used without further purification. ^1^H NMR spectra were recorded with a JEOL ECZ 500 (^1^H: 500 MHz and ^13^C: 125 MHz) and a Varian VNMR S600 (^1^H: 600 MHz and ^13^C: 150 MHz) at *T* = 25 °C. The ^1^H NMR and ^13^C{1H} NMR spectra were referenced to the residual proton signal of the solvent [CD_3_CN: δ(^1^H) = 1.94 ppm, δ(^13^C) = 118.36 ppm or DMSO-*d*_5_: δ(^1^H) = 2.50 ppm, δ(^13^C) = 39.52 ppm] or to an internal standard in CDCl_3_ [TMS: δ(^1^H) = 0.00 ppm, δ(^13^C) = 0.00 ppm]. Structural assignments were made with additional information from gCOSY, gHSQC, and gHMBC experiments. The spectra were processed with the MestreNova software. The mass spectra were recorded with a Finnigan LCQ Deca (driving current: 6 kV, collision gas: argon, capillary temperature: 200 °C, support gas: nitrogen) and an Orbitrap mass spectrometer Thermo Fisher Exactive (driving current: 3.5 kV, capillary temperature: 300 °C, capillary voltage: 45 V, injection rate: 5 μL/min, scanning range: 150−750 *m*/*z*, and resolution: ultra-high) and processed with the software Xcalibur. The CHNS analysis data were determined in-house with a HEKAtech EuroEA combustion analyzer. The melting points were measured with a melting point apparatus BÜCHI 545 (Büchi, Flawil, CH) and are uncorrected. The absorption spectra were recorded on a Varian Cary 100 Bio absorption spectrometer with Hellma quartz glass cuvettes 110-QS (layer thickness *d* = 10 mm). The emission spectra were recorded on a Varian Cary Eclipse fluorescence spectrometer with Hellma quartz glass cuvettes 115 FQS (layer thickness *d* = 10 mm). All measurements were recorded at *T* = 20 °C as adjusted with a thermostat, if not stated otherwise. The sample solutions in the DNA experiments were mixed with a reaction vessel shaker Top-Mix 11118 (Fisher Bioblock Scientific). E-Pure water was obtained with an ultrapure water system D 4632-33 (Wilhelm Werner GmbH, Leverkusen, Germany) with filters D 0835, D 0803, and D 5027 (2×). CD- and LD-spectroscopic measurements were performed on a Chirascan spectrometer (Applied Photophysics). For LD-spectroscopic experiments, the spectrometer was equipped with a High Shear Couette Cell Accessory. The samples were oriented in a rotating Couette with a shear gradient of 1200 s^−1^. Photochemical reactions were carried out with a high-pressure Hg lamp (Heraeus TQ 150) in a quartz glass photoreactor or in a cuvette. Photoreactions for the investigation of the DNA damage were performed with a Rayonet (RPR-100) photoreactor equipped with 12 ultraviolet lamps (8 W, λ_exc_ = 366 nm).

### Synthesis

#### General procedure (GP)

A solution of the styrylpyridine derivatives (*c* = 0.24–0.95 mM) in MeCN or in a mixture of MeCN/H_2_O was saturated with oxygen gas for 5–15 min, and the solutions were irradiated in an immersion-well photoreactor with a high-pressure Hg lamp. The reaction was controlled by absorption spectroscopy. After completion of the reaction, the solvent was removed by distillation, and the residue was purified by washing with *n*-pentane, *n*-hexane or *n*-hexane and EtOAc and subsequently by column chromatography or recrystallization from MeOH with addition of HClO_4_.

#### 2-Methoxycarbonyl-8,9-dimethoxybenzo[*c*]quinolizinium perchlorate (**3c**)

According to GP, a solution (*c* = 0.80 mM) of **2c** (100 mg, 334 µmol) in MeCN/H_2_O (6:1, 400 mL) was irradiated for 49 min. The crude product was washed with *n*-hexane (2 × 3 mL) and EtOAc (3 × 5 mL) and recrystallized from MeOH with addition of HClO_4_ to give the product as brown amorphous solid (14 mg, 35 µmol, 10%); mp > 200 °C (decomp.); ^1^H NMR (600 MHz, CD_3_CN) δ 4.07 (s, 3H, OCH_3_), 4.09 (s, 3H, CO_2_CH_3_), 4.23 (s, 3H, OCH_3_), 7.71 (s, 1H, 7-H), 8.11 (s, 1H, 10-H), 8.15 (d, ^3^*J* = 9 Hz, 1H, 5-H), 8.49 (d, ^3^*J* = 9 Hz, 1H, 4-H), 8.61 (d, ^3^*J* = 9 Hz, 1H, 6-H), 8.67 (dd, ^3^*J* = 9 Hz, ^4^*J* = 1 Hz, 1H, 3-H), 10.15 (s, 1H, 1-H); ^13^C NMR (150 MHz, CD_3_CN) δ 54.3 (CO_2_*C*H_3_), 57.4 (OCH_3_), 58.2 (OCH_3_), 100.1 (C10), 110.1 (C7), 121.5 (C5), 124.4 (C6a), 127.3 (C2), 129.8 (C4), 131.9 (C10a), 135.4 (C1), 136.4 (C3), 139.5 (C6), 144.8 (C4a), 153.4 (C8), 156.4 (C9), 164.1 (*C*O_2_CH_3_); MS (ESI^+^) *m/z*: [M^+^] 298 (100); Anal. calcd for C_17_H_16_ClNO_8_·0.5HClO_4_: C, 45.58; H, 3.71; N, 3.13; found: C, 45.56; H, 3.89; N, 3.32.

#### 2-Cyano-8,9-dimethoxybenzo[*c*]quinolizinium perchlorate (**3d**)

According to GP, a solution (*c* = 0.24 mM) of **2d** (30.0 mg, 110 µmol) in H_2_O/MeCN (6:1, 420 mL) was irradiated for 35 min. The product was purified by recrystallization from MeOH with addition of HClO_4_ and by column chromatography (SiO_2_, CHCl_3_/MeOH 95:5 → 90:10). The crude product was filtered through celite, washed with *n*-pentane (3 × 10 mL), suspended in CHCl_3_ (1 mL) and filtered to give the product as brown amorphous solid, containing small amounts of impurities according to ^1^H NMR-spectroscopic analysis (4.0 mg, 11 µmol, 10%); ^1^H NMR (500 MHz, CD_3_CN) δ 4.06 (s, 3H, OCH_3_), 4.20 (s, 3H, OCH_3_), 7.73 (s, 1H, 10-H), 8.08 (s, 1H, 7-H), 8.15 (d, ^3^*J* = 9 Hz, 1H, 6-H), 8.41 (dd, ^3^*J* = 9 Hz, ^4^*J* = 1 Hz, 1H, 3-H), 8.50 (d, ^3^*J* = 9 Hz, 1H, 4-H), 8.67 (d, ^3^*J* = 9 Hz, 1H, 5-H), 10.22 (s, 1H, 1-H).

#### 2-Chloro-8,9-dimethoxybenzo[*c*]quinolizinium perchlorate (**3e**)

According to GP, a solution (*c* = 0.70 mM) of **2e** (44 mg, 0.16 mmol) in MeCN (300 mL) was irradiated for 35 min. The crude product was washed with *n*-hexane (3 mL) and recrystallized from MeOH with addition of HClO_4_. The remaining solid was dissolved in H_2_O (50 mL), filtered from a black precipitate and a saturated aq solution of NaBF_4_ (7 mL) was added to the solution. The aqueous layer was extracted with MeNO_2_ (2 × 30 mL) and the combined organic layers were washed with H_2_O (2 × 30 mL) and dried with Na_2_SO_4_. The solvent was evaporated to give a yellow oil. The residue was suspended in CHCl_3_ (1 mL) and the remaining solid was filtered off to give the product as yellow amorphous solid (2.5 mg, 6.9 µmol, <5%); mp 164–166 °C (decomp.); ^1^H NMR (500 MHz, CD_3_CN) δ 4.07 (s, 3H, OCH_3_), 4.20 (s, 1H, OCH_3_), 7.68 (s, 1H, 7-H), 8.04 (s, 1H, 10-H), 8.10 (d, ^3^*J* = 9 Hz, 1H, 5-H), 8.31 (dd, ^3^*J* = 9 Hz, ^4^*J* = 1 Hz, 1H, 3-H), 8.40 (d, ^3^*J* = 9 Hz, 1H, 4-H), 8.53 (d, ^3^*J* = 9 Hz, 1H, 6-H), 9.88 (d, ^4^*J* = 1 Hz, 1H, 1-H); ^13^C NMR (125 MHz, CD_3_CN) δ 57.4 (OCH_3_), 58.3 (OCH_3_), 100.0 (C10), 109.9 (C7), 121.5 (C5), 124.5 (C6a), 130.2 (C4), 131.0 (C10a), 131.8 (C1), 132.7 (C2), 137.8 (C6), 138.4 (C3), 142.0 (C4a), 153.5 (C8), 156.4 (C9); MS (ESI^+^) *m*/*z*: [M^+^] 274 (100); HRMS–ESI^+^ (*m*/*z*): [M]^+^ calcd. for C_15_H_13_NO_2_Cl, 274.0629; found, 274.0625.

#### 2-Ethyl-8,9-dimethoxybenzo[*c*]quinolizinium perchlorate (**3f**)

According to GP, a solution (*c* = 0.95 mM) of **2f** (102 mg, 379 µmol) in MeCN (400 mL) was irradiated for 45 min and the crude product was washed with *n*-hexane (3 mL) and recrystallized from MeOH with addition of HClO_4_ to give a yellow amorphous solid (29 mg, 79 µmol, 21%); mp 215–217 °C (decomp.); ^1^H NMR (500 MHz, CD_3_CN) δ 1.42 (t, ^3^*J* = 8 Hz, 3H, CH_3_), 3.08 (q, ^3^*J* = 8 Hz, 2H, CH_2_), 4.05 (s, 3H OCH_3_), 4.20 (s, 1H, OCH_3_), 7.63 (s, 1H, 7-H), 8.01 (d, ^3^*J* = 9 Hz, 1H, 5-H), 8.05 (s, 1H, 10-H), 8.26 (dd, ^3^*J* = 9 Hz, ^4^*J* = 2 Hz, 1H, 3-H), 8.33 (d, ^3^*J* = 9 Hz, 1H, 4-H), 8.40 (d, ^3^*J* = 9 Hz, 1H, 6-H), 9.56 (s, 1H, 1-H); ^13^C NMR (125 MHz, CD_3_CN) δ 15.6 (CH_3_), 27.0 (CH_2_), 57.3 (OCH_3_), 58.1 (OCH_3_), 99.9 (C10), 110.0 (C7), 121.5 (C5), 124.0 (C6a), 129.0 (C4), 130.9 (C10a), 131.4 (C1), 136.2 (C6), 139.3 (C3), 141.9 (C4a), 142.1 (C2), 152.9 (C9), 155.7 (C8); MS (ESI^+^) *m/z*: [M^+^] 268 (100); Anal. calcd for C_17_H_18_ClNO_6_: C, 55.52; H, 4.93; N, 3.81; found: C, 55.71; H, 4.89; N, 3.83.

#### 2-Acetylamino-8,9-dimethoxybenzo[*c*]quinolizinium tetrafluoroborate (**3g**)

According to GP, a solution (*c* = 0.25 mM) of (*E*)-5-acetylamino-2-(3,4-dimethoxystyryl)pyridine (30.0 mg, 101 µmol) in a mixture of MeCN/H_2_O (7:3, 400 mL) was irradiated for 33 min and the crude product was washed with *n*-hexane (3 mL) and redissolved in dest. H_2_O (5 mL). A saturated aq solution of NaBF_4_ (3 mL) was added to the solution. A brown precipitate was removed by filtration. The filtrate was extracted with MeNO_2_ (3 × 10 mL) and the solvent was evaporated to give a brown solid, which was purified by column chromatography (SiO_2_, CHCl_3_/MeOH, 95:5, *R*_f_ = 0.1) and washed with pentane (3 × 1 mL) to give the product as yellow amorphous solid (5.0 mg, 13 µmol, 13%); mp > 260 °C (decomp.); ^1^H NMR (500 MHz, CD_3_CN) δ 2.27 (s, 3H, COCH_3_), 4.05 (s, 3H, OCH_3_), 4.17 (s, 3H, OCH_3_), 7.61 (s, 1H, 7-H), 7.85 (s, 1H, 10-H), 7.97 (d, ^3^*J* = 9 Hz, 1H, 5-H), 8.30 (d, ^3^*J* = 9 Hz, 1H, 4-H), 8.34 (d, ^3^*J* = 9 Hz, 1H, 6-H), 8.36 (dd, ^3^*J* = 9 Hz, ^4^*J* = 1 Hz, 1H, 3-H), 9.48 (brs, 1H, NH), 10.45 (d, ^4^*J* = 1 Hz, 1H, 1-H); ^13^C NMR (125 MHz, CD_3_CN) δ 24.4 (CO*C*H_3_), 57.3 (OCH_3_), 57.5 (OCH_3_), 99.3 (C10), 110.0 (C7), 121.4 (C5), 121.9 (C1), 124.2 (C6a), 129.5 (C4), 130.6 (C3), 135.2 (C6), 137.3 (C4a), 139.5 (C10a), 153.1 (C9), 155.6 (C8), 171.5 (*C*OCH_3_); HRMS–ESI^+^ (*m*/*z*): [M]^+^ calcd. for C_17_H_17_N_2_O_3_^+^, 297.1234; found, 297.1235.

## Supporting Information

File 1 Detailed experimental procedures, additional spectroscopic data and ^1^H NMR spectra.

## Data Availability

All data that supports the findings of this study is available in the published article and/or the supporting information to this article.
