# Supplementary material for: Photoinduced in situ generation of DNA-targeting ligands: DNA-binding and DNA-photodamaging properties of benzo[c]quinolizinium ions
Source: Beilstein J Org Chem. 2024 Jan 18;20:101–17. doi: 10.3762/bjoc.20.11 (PMC10804566; doi:10.3762/bjoc.20.11)
Supplement: File 1 — Detailed experimental procedures, additional spectroscopic data and 1H NMR spectra. [file Beilstein_J_Org_Chem-20-101-s001.pdf]

BEILSTEIN JOURNAL OF ORGANIC CHEMISTRY

Supporting Information

for

Photoinduced in situ generation of DNA-targeting ligands: DNA-binding and DNA-photodamaging properties of benzo[*c*]quinolizinium ions

Julika Schlosser, Olga Fedorova, Yuri Fedorov and Heiko Ihmels

Beilstein J. Org. Chem. **2024,** *20,* 101–117. doi:10.3762/bjoc.20.11

Detailed experimental procedures, additional spectroscopic data and ^{1}H NMR spectra

License and Terms: This is a supporting information file under the terms of the Creative Commons Attribution License (https://creativecommons.org/licenses/by/4.0). Please note that the reuse, redistribution and reproduction in particular requires that the author(s) and source are credited and that individual graphics may be subject to special legal provisions.
The license is subject to the *Beilstein Journal of Organic Chemistry* terms and conditions: (https://www.beilstein-journals.org/bjoc/terms)

Table of contents

1. Materials..S2
2. Synthesis..S2
3. Photometric data S6
3.1. Absorption properties S6
3.2. Photoreactions followed by absorption spectroscopy S6
4. In situ photoreactions in the presence of ct DNA..S9
5. Photometric and fluorimetric DNA titrations S10
6. CD and LD spectra..S12
7. Gel electrophoresis S13
8. NMR spectra S18
9. References..S29

1. Materials

$Ca(OTf)_2$ was synthesized according to literature protocol.[1]

Commercially available reagents were purchased from the following companies:

– Alfa Aesar GmbH & Co KG (Haverhill, USA): veratraldehyde, Pd/C, hydrazine hydrate, acetyl chloride.

– BLD Pharmatech. Ltd (Shanghai, China): 2-methyl-5-nitropyridine, 6-methyl-3-pyridine-carbonitrile, 5-methoxycarbonyl-2-methylpyridine.

– Merck KGaA (Darmstadt, Germany): pyridine, CuCl, Bu_4NPF_6, gel loading buffer.

– Thermo Fisher Scientific (Waltham, MA, USA): $NaNO_2$, piperidine, 5-ethyl-2-methyl-pyridine.

Anhydrous THF was stored over Na wire and distilled prior to use.

All other reactants and solvents were used without further purification.

Calf thymus DNA (ct DNA, type I; highly polymerized sodium salt; ε = 12 824 cm^{-1} M^{-1}) was purchased from Sigma-Aldrich (St. Louis, USA) and pBR322 plasmid DNA (lyophilized) was purchased from Carl Roth and used without further purification.

2. Synthesis

(*E*)-5-Nitro-2-(3,4-dimethoxystyryl)pyridine (2a)

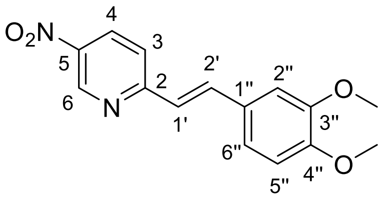

A solution of **1a** (100 mg, 720 µmol), veratraldehyde (180 mg, 1.08 mmol) and piperidine (70 µL) in MeOH (5 mL) was stirred under reflux for 12 h. The formed precipitate was isolated by filtration and washed with MeOH (10 mL) to give **2a** as dark orange-colored microcrystalline solid (130 mg, 460 µmol, 65%); mp 151–152 °C. – ^{1}H NMR (500 MHz, $CDCl_3$): δ = 3.94 (s, 3H, OCH_3), 3.96 (s, 3H, OCH_3), 6.91 (d, ^3J = 8 Hz, 1H, 5“-H), 7.11 (d, ^3J = 16 Hz, 1H, 1-H), 7.17 (d, ^4J = 2 Hz, 1H, 2“-H), 7.20 (dd, ^3J = 8 Hz, ^4J = 2 Hz, 1H, 6“-H), 7.47 (d, ^3J = 9 Hz, 1H, 3-H), 7.81 (d, ^3J = 16 Hz, 1H, 2‘-H), 8.42 (dd, ^3J = 9 Hz, ^4J = 3 Hz, 1H, 4-H), 9.39 (d, ^4J = 3 Hz, 1H, 6-H). – ^{13}C NMR (125 MHz, $CDCl_3$): δ = 56.0 (2 × OCH_3), 109.5 (C2“), 111.2 (C5“), 121.2 (C3), 122.1 (C6“), 123.8 (C1’), 128.7 (C1“), 131.7 (C4), 138.1 (C2”), 142.1 (C5), 145.5 (C6), 149.3 (C3“), 150.7 (C4“), 161.3 (C2). – MS (ESI$^+$): *m/z* (%) = 287 (100) [M – H^+]. – El. Anal. for $C_{15}H_{14}N_2O_4$ (286.3), calcd (%): C 62.93, H 4.93, N 9.79; found (%): C 62.96, H 4.76, N 9.53.

(*E*)-5-Amino-2-(3,4-dimethoxystyryl)pyridine (2b)

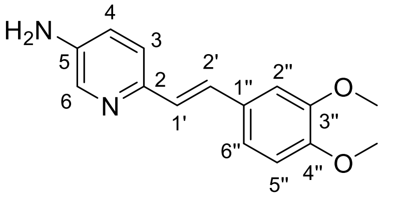

A solution of **2a** (250 mg, 860 µmol) and hydrazine hydrate (104 µL, 2.15 mmol) in MeOH (30 mL) was heated to 70 °C, and Pd/C (65 mg) in MeOH (1 mL) was added. The reaction mixture was stirred under reflux for 1.5 h, cooled to room temperature and the catalyst was removed by filtration through a pad of celite. The solvent was removed by destillation to give **2b** as yellow solid (180 mg, 700 µmol, 83%). An analytically pure sample was obtained from recrystallization from EtOH to give a yellow microcrystalline solid; mp 168–169 °C. – ^{1}H NMR (500 MHz, DMSO-d_6): δ = 3.76 (s, 3H, OCH_3), 3.81 (s, 3H, OCH_3), 5.43 (br s, 2H, NH_2), 6.89 (dd, ^{3}J = 8 Hz, ^{4}J = 3 Hz, 1H, 4-H), 6.92 (d, ^{3}J = 8 Hz, 1H, 5'-H), 6.99 (d, ^{3}J = 16 Hz, 1H, 1'-H), 7.04 (dd, ^{3}J = 8 Hz, ^{4}J = 2 Hz, 1H,6''-H), 7.18 and 7.19 (overlapping d, 2H, ^{4}J = 2 Hz, 2''-H, ^{3}J = 8 Hz, 3-H), 7.22 (d, ^{3}J = 16 Hz, 1H, 2'-H), 7.94 (d, ^{4}J = 3 Hz, 1H, 6-H). – ^{13}C NMR (125 MHz, DMSO-d_6): δ = 55.4 (OCH_3), 55.5 (OCH_3), 109.1 (C2''), 111.9 (C5''), 119.6 (C6''), 120.0 (C4), 122.3 (C3), 126.5 (C1' and C2'), 130.3 (C1''), 136.1 (C6), 143.4 (C2), 143.7 (C5), 148.4 (C4''), 148.9 (C3''). – MS (ESI$^+$): *m/z* (%) = 257 (100) [M – H$^+$]. – El. Anal. for $C_{15}H_{16}N_2O_2$ (256.1), calcd (%) C 70.29, H 6.29, N 10.93; found (%) C 69.99, H 6.35, N 10.74.

(*E*)-5-Methoxycarbonyl-2-(3,4-dimethoxystyryl)pyridine (2c)

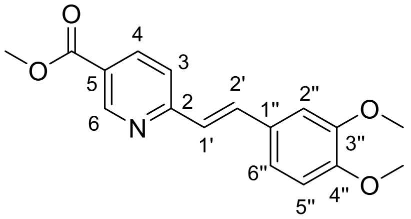

A solution of **1c** (100 mg, 660 µmol), veratraldehyde (160 mg, 990 µmol) and piperidine (70 µL) in MeOH (5 mL) was stirred under reflux for 3 d. Upon cooling, a colorless precipitate formed, which was isolated by filtration (71.0 mg, 240 µmol, 36%). An analytically pure sample was obtained by recrystallization from EtOH to give **2c** as a colorless amorphous solid; mp 132–133 °C. – ^{1}H NMR (500 MHz, $CDCl_3$): δ = 3.92 (s, 3H, OCH_3), 3.94 (s, 3H, OCH_3), 3.95 (s, 3H, OCH_3), 6.89 (d, ^{3}J = 9 Hz, 1H, 5'-H), 7.09 (d, ^{3}J = 16 Hz, 1H, 1'-H), 7.16 (overlapping d and dd, 2H, 2''-H, 6''-H), 7.43 (d, ^{3}J = 8 Hz, 1H, 3-H), 7.70 (d, ^{3}J = 16 Hz, 1H, 2'-H), 8.24 (dd, ^{3}J = 8 Hz, ^{4}J = 2 Hz, 1H, 4-H), 9.17 (d, ^{3}J = 2 Hz, 1H, 6-H). – ^{13}C NMR (125 MHz, $CDCl_3$): δ = 52.3 (OCH_3), 55.9 (OCH_3), 56.0 (OCH_3), 109.3 (C2''), 111.2 (C5''), 121.0 (C3), 121.5 (C6''), 123.6 (**C**O_2CH_3), 125.1 (C1'), 129.2 (C1''), 135.5 (C2'), 137.6 (C4), 149.2 (C3''), 150.2 (C4''), 151.0 (C6), 159.6 (C2), 165.9 (C5). – MS (ESI$^+$): *m/z* (%) = 300 (100) [M – H$^+$]. – El. Anal. for $C_{17}H_{17}NO_4$ (299.1), calcd (%) C 68.22, H 5.72, N 4.68; found (%) C 68.13, H 5.43, N 4.55.

(*E*)-5-Cyano-2-(3,4-dimethoxystyryl)pyridine (2d)

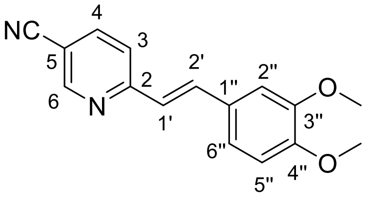

A mixture of **1d** (500 mg, 4.34 mmol), veratraldehyde (840 mg, 5.08 mmol), $Ca(OTf)_2$ (76.5 mg, 210 µmol) and Bu_4NPF_6 (33.0 mg, 84.0 µmol) was stirred at 130 °C for 4 d. The reaction mixture was dissolved in EtOAc (100 mL), washed with aq. NH_4Cl (100 mL), and the aqueous layer was extracted with EtOAc (3 × 50 mL). The combined organic layers were dried with Na_2SO_4 and the solvent was removed by destillation to give a brown oil. The crude product was purified by column chromatography (SiO_2, *n*-hexane/EtOAc 4:1 → 1:1) to give **2d** as yellow solid (470 mg, 1.76 mmol, 42%). An analytically pure sample was obtained from recrystallization from EtOH as yellow needles; mp 115–116 °C. – ^1H NMR (500 MHz, $CDCl_3$): δ = 3.92 (s, 3H, OCH_3), 3.95 (s, 3H, OCH_3), 6.90 (d, ^3J = 8 Hz, 1H, 5''-H), 7.03 (d, ^3J = 16 Hz, 1H, 1'-H), 7.15 (overlapping d and dd, 2H, 2''-H, 6''-H), 7.41 (dd, ^3J = 8 Hz, ^5J = 1 Hz, 1H, 3-H), 7.74 (d, ^3J = 16 Hz, 1H, 2'-H), 7.87 (dd, ^3J = 8 Hz, ^4J = 2 Hz, 1H, 4-H), 8.81 (dd, ^4J = 2 Hz, ^5J = 1 Hz, 1H, 6-H). – ^{13}C NMR (125 MHz, $CDCl_3$): δ = 55.9 (OCH_3), 56.0 (OCH_3), 106.8 (CN), 109.4 (C2''), 111.2 (C5''), 117.3 (C5), 121.2 (C3), 121.9 (C6''), 124.2 (C1'), 128.8 (C1''), 137.0 (C2'), 139.5 (C4), 149.3 (C4''), 150.5 (C3''), 152.4 (C6), 159.2 (C2). – MS (ESI$^+$): *m/z* (%) = 267 (100) [M – H$^+$]. – El. Anal. for $C_{16}H_{14}N_2O_2$ (266.1), calcd (%) C 72.17, H 5.30, N 10.52; found (%) C 72.05, H 5.19, N 10.48.

(*E*)-5-Chloro-2-(3,4-dimethoxystyryl)pyridine (2e)

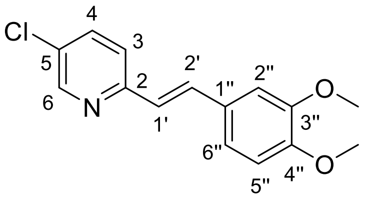

To a suspension of **2b** (250 mg, 980 µmol) in aq. HCl (19%, 10 mL) was added a solution of $NaNO_2$ (80.0 mg, 1.18 mmol) in H_2O (1 mL) at 0 °C. After stirring for 30 min at 0 °C, an ice-cooled solution of CuCl (290 mg, 2.94 mmol) in HCl (37%, 5 mL) was added and the mixture was stirred for 2 h at room temperature and 30 min at 60 °C. After cooling to room temperature, the reaction mixture was extracted with CH_2Cl_2 (3 × 50 mL). The combined organic layers were dried with Na_2SO_4 and the solvent was removed by destillation to give a yellow solid. The residue was purified by column chromatography (SiO_2, *n*-hexane/EtOAc 10:1→1:1) to give **2e** as yellow microcrystalline solid (53.0 mg, 192 µmol, 20%). An analytically pure sample was obtained from recrystallization from MeOH as fine colorless needles; mp 95–97 °C. – ^1H NMR (500 MHz, $CDCl_3$): δ = 3.91 (s, 3H, OCH_3), 3.94 (s, 3H, OCH_3), 6.88 (d, ^3J = 8 Hz, 1H, 5'-H), 7.00 (d, ^3J = 16 Hz, 1H, 1'-H), 7.12 (overlapping dd, ^4J = 2 Hz, 1H, 6''-H), 7.13 (s, 1H, 2''-H), 7.32 (d, ^3J = 9 Hz, 1H, 3-H), 7.53 (d, ^3J = 16 Hz, 1H, 2'-H), 7.62 (dd, ^3J = 8 Hz, ^4J = 3 Hz, 1H, 4-H), 8.53 (d, ^4J = 3 Hz, 1H, 6-H). – ^{13}C NMR (125 MHz, $CDCl_3$): δ = 55.9 (OCH_3), 56.0 (OCH_3), 109.1 (C2''), 111.2 (C5''), 121.0 (C6''), 122.1 (C3), 124.8 (C1'), 129.4 (C1''), 129.6 (C5), 133.3 (C2'), 136.2 (C4), 148.5 (C6), 149.2 (C4''),

149.8 (C3”), 154.2 (C2). – MS (ESI[+]): *m/z* (%) = 276 (100) [M – H^+]. – El. Anal. for $C_{15}H_{14}ClNO_2$ (275.1), calcd (%) C 65.34, H 5.12, N 5.08; found (%) C 64.95, H 5.15, N 5.00.

(*E*)-5-Ethyl-2-(3,4-dimethoxystyryl)pyridine (2f)

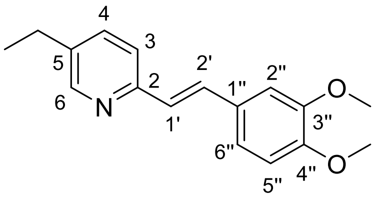

A mixture of **1f** (500 mg, 4.13 mmol), veratraldehyde (820 mg, 4.95 mmol), $Ca(OTf)_2$ (76.0 mg, 210 µmol) and Bu_4NPF_6 (31.0 mg, 80.1 µmol) was stirred for 5 d. The reaction mixture was dissolved in EtOAc (50 mL) and washed with aq. NH_4Cl (50 mL). The aqueous layer was washed with EtOAc (3 × 50 mL). The combined organic layers were dried with Na_2SO_4 and the solvent was removed by destillation to give a brown oil. The crude product was purified by column chromatography (SiO_2, *n*-hexane/EtOAc 4:1; R_f = 0.55) to give the product as colorless microcrystalline solid (140 mg, 520 µmol, 13%). An analytically pure sample was obtained from recrystallization from EtOH and subsequent recrystallization from Et_2O to give the product as colorless crystalline plates; mp 80–83 °C. – ^{1}H NMR (500 MHz, $CDCl_3$): δ = 1.27 (t, ^3J = 8 Hz, 3H, CH_3), 2.66 (q, ^3J = 8 Hz, 2H, CH_2), 3.91 (s, 3H, OCH_3), 3.94 (s, 3H, OCH_3), 6.87 (d, ^3J = 8 Hz, 1H, 5”-H), 7.04 (d, ^3J = 16 Hz, 1H, 1’-H), 7.11 (dd, ^3J = 8 Hz, ^4J = 2 Hz, 1H, 6”-H), 7.14 (d, ^4J = 2 Hz, 1H, 2”-H), 7.32 (d, ^3J = 8 Hz, 1H, 3-H), 7.49 (dd, ^3J = 8 Hz, ^4J = 2 Hz, 1H, 4-H), 7.50 (d, ^3J = 16 Hz, 1H, 2’-H), 8.43 (d, ^3J = 2 Hz, 1H, 6-H). – ^{13}C NMR (125 MHz, $CDCl_3$): δ = 15.3 (CH_3), 25.9 (CH_2), 55.9 (OCH_3) , 56.0 (OCH_3), 109.0 (C2”), 111.2 (C5”), 120.7 (C6”), 121.2 (C3), 126.2 (C1’), 130.0 (C1”), 131.5 (C2’), 135.8 (C4), 137.5 (C5), 149.2 (C3”), 149.4 (C6), 149.4 (C4”), 153.5 (C2). – MS (ESI[+]): *m/z* (%) = 270 (100) [M – H^+]. – El. Anal. for $C_{17}H_{19}NO_2$ (269.3) calcd (%) C 75.81, H 7.11, N 5.20; found (%) C 75.67, H 7.28, N 4.86.

(*E*)-5-Acetylamino-2-(3,4-dimethoxystyryl)pyridine (2g)

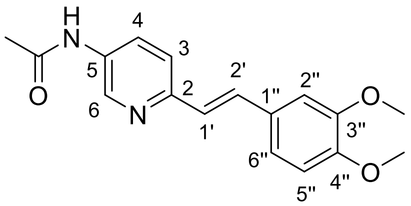

A solution of acetyl chloride (27.8 µL, 390 µmol), pyridine (31.4 µL, 390 µmol), and **2b** (100 mg, 390 µmol) in anhydrous THF (5 mL) was stirred at room temp. for 3 h. Water (15 mL) was added and the product was extracted with CH_2Cl_2 (3 × 10 mL). The combined organic layer was washed with water (10 mL), dried with Na_2SO_4, filtered and the solvent was removed by destillation to give a yellow oil. The crude product was purified by column chromatography (SiO_2, *n*-hexane/EtOAc 1:50, R_f = 0.34) to give **2g** as orange-colored amorphous solid (33.0 mg, 110 µmol, 28%). An analytically pure sample was obtained from recrystallization from EtOH to give a light-yellow amorphous solid; mp 153–155 °C. – ^{1}H NMR (500 MHz, $CDCl_3$): δ = 2.22 (s, 3H, $COCH_3$), 3.90 (s, 3H, OCH_3), 3.93 (s, 3H, OCH_3), 6.86 (d, ^3J = 8 Hz, 1H, 5”-H), 7.01 (d, ^3J = 16 Hz, 1H, 2’-H), 7.09 (dd, ^3J = 8 Hz, ^4J = 2 Hz, 1H, 6”-H), 7.11 (d, ^4J = 2 Hz, 1H, 2”-H), 7.37 (d, ^3J = 9 Hz, 1H, 3-H), 7.44 (d, ^3J = 16 Hz, 1H,

1'-H), 7.61 (br s, 1H, NH), 8.17 (dd, ^3J = 9 Hz, ^4J = 2 Hz, 1H, 4-H), 8.50 (d, ^4J = 2 Hz, 1H, 6-H). – ^{13}C NMR (125 MHz, $CDCl_3$): δ = 24.5 (CO**C**H_3), 55.9 (OCH_3), 56.0 (OCH_3), 109.0 (C2''), 111.2 (C5''), 120.8 (C6''), 121.6 (C3), 125.4 (C2'), 127.8 (C4), 129.8 (C1''), 131.8 (C1'), 133.1 (C5), 140.7 (C6), 149.2 (C3''), 149.5 (C4''), 151.8 (C2), 168.7 (**C**OCH_3). – MS (ESI^+): *m/z* (%) = 299 (100) [M – H^+]. – El. Anal. for $C_{17}H_{18}N_2O_3$ (298.3) calcd (%) C 68.44, H 6.08, N 9.39; found (%) C 68.41, H 6.18, N 9.25.

3. Photometric data

3.1. Absorption properties

Table S1: Absorption data for derivatives **2a**–**g** and **3c**–**g** and binding constants of **3c**, **3e**–**g** with ct DNA.

	λ_{abs}/nm[a,b]	λ_{abs}/nm[a,c]		λ_{abs}/nm[a,b]	$\Delta\lambda$ / nm[b]	K_b / 10^4 M^{-1}[d]
**2a**	394					
**2b**	347	343				
**2c**	352		**3c**	402	50	5.8
**2d**	360	357	**3d**	407	47	
**2e**	340	336	**3e**	397	57	6.6
**2f**	333	332	**3f**	389	56	14
**2g**	343	340	**3g**	398	55	5.7

[a] Long-wavelength absorption maximum; *c* = 20 µM. [b] In MeCN. [c] In H_2O. [d] Determined from fluorimetric titrations.

3.2. Photoreactions followed by absorption spectroscopy

Solutions were prepared for each measurement from stock solutions of the derivatives **2a**–**g** in MeCN (c = 1.0 mM). Aliquots of the stock solution were thoroughly evaporated under a stream of nitrogen, and the residue was redissolved in the respective solvent or solvent mixture. The solutions were irradiated in a quartz cuvette with a high-pressure Hg-lamp and the reaction progress was monitored by absorption spectroscopy. The absorption spectra were recorded in a range of 200–450 nm with a collection rate of 120 nm / min.

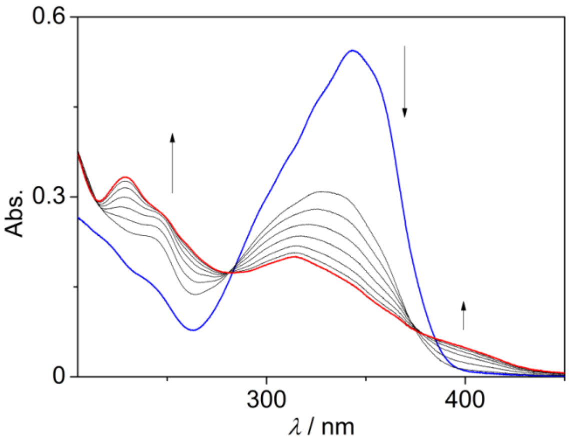

Figure S1: Spectral changes during the irradiation of **2b** (*c* = 20 µM in MeOH) for 7 min. Blue: spectrum of the starting material before irradiation; red: spectrum at the end of the irradiation.

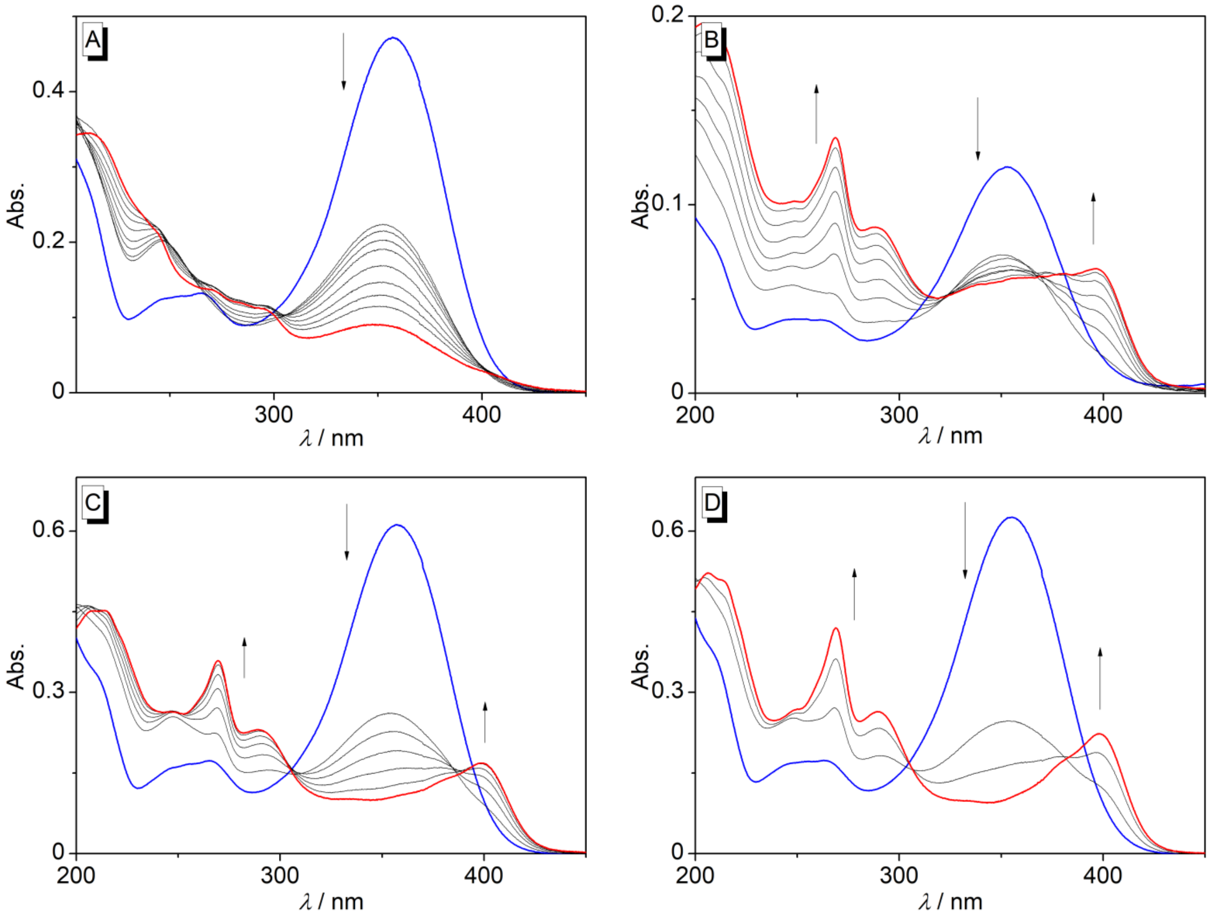

Figure S2: Spectral changes during the irradiation of **2c** (c = 20 µM) in MeOH for 16 min (A), in H_2O for 7 min (B), in MeCN/H_2O 1:1 for 6 min (C) and in MeCN/H_2O 1:9 for 3 min (D). Blue: spectrum of the starting material before irradiation; red: spectrum at the end of the irradiation.

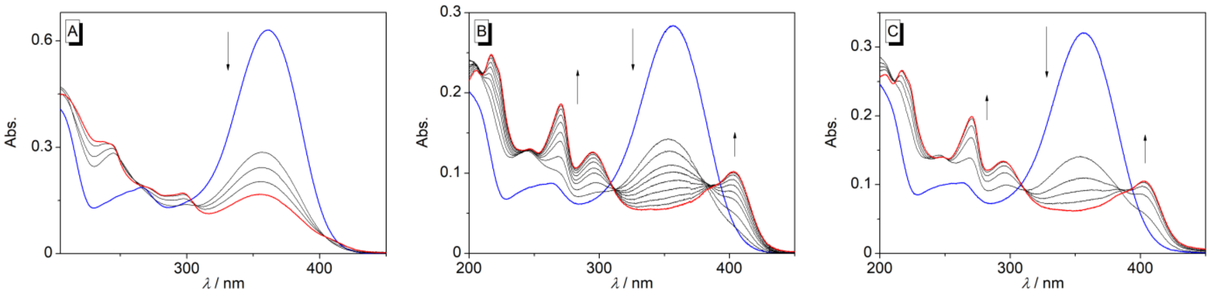

Figure S3: Spectral changes during the irradiation of **2d** (c = 20 µM) in MeOH for 8 min (A), in H_2O for 9 min (B) and in Na phosphate buffer for 50 sec (C). Blue: spectrum of the starting material before irradiation; red: spectrum at the end of the irradiation.

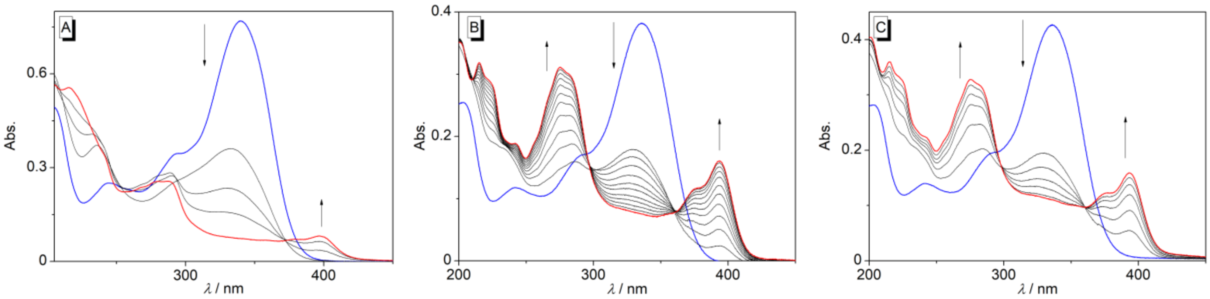

Figure S4: Spectral changes during the irradiation of **2e** (c = 20 μM) in MeOH for 47 min (A), in H_2O for 11 min (B) and in Na phosphate buffer for 60 sec (C). Blue: spectrum of the starting material before irradiation; red: spectrum at the end of the irradiation.

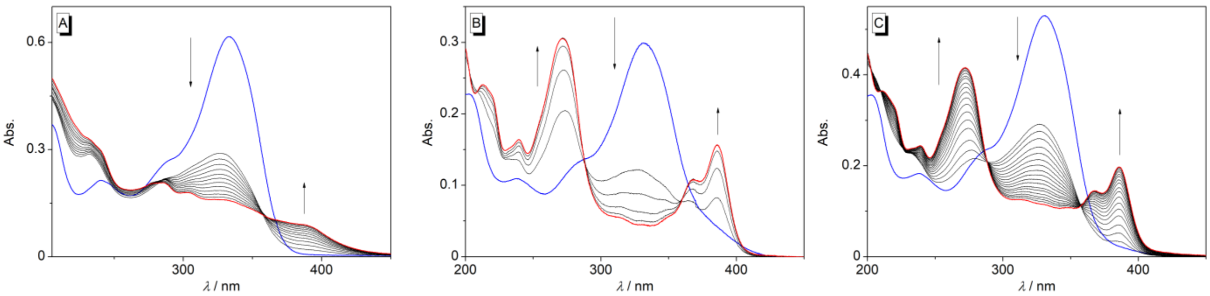

Figure S5: Spectral changes during the irradiation of **2f** (c = 20 μM) in MeOH for 10 min (A), in H_2O for 5 min (B) and in Na phosphate buffer for 180 sec (C). Blue: spectrum of the starting material before irradiation; red: spectrum at the end of the irradiation.

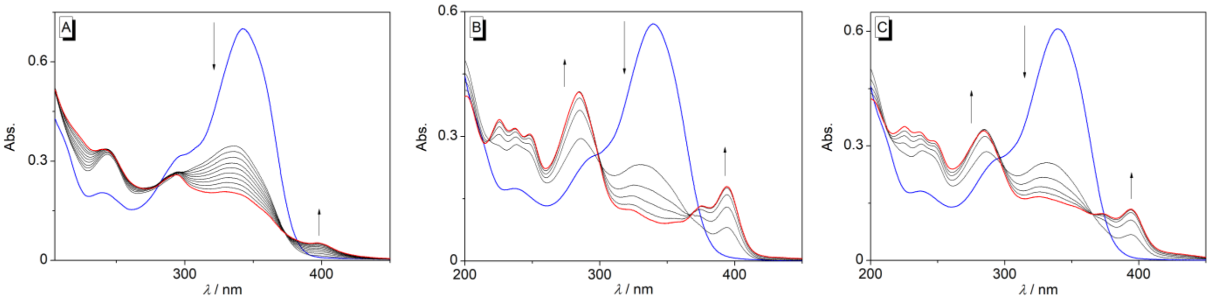

Figure S6: Spectral changes during the irradiation of **2g** (c = 20 μM) in MeOH for 10 min (A), in H_2O for 150 sec (B) and in Na phosphate buffer for 50 sec (C). Blue: spectrum of the starting material before irradiation; red: spectrum at the end of the irradiation.

4. In situ photoreactions in the presence of ct DNA

Solutions were prepared for each measurement from stock solutions of the derivatives **2d–g** in MeCN (c = 1.0 mM). Aliquots of the stock solution were thoroughly evaporated under a stream of nitrogen, and the residue was redissolved in Na phosphate buffer containing ct DNA. The solutions were irradiated in a quartz cuvette with a high-pressure Hg-lamp (Heraeus TQ 150) and the reaction progress was monitored by absorption and CD spectroscopy.

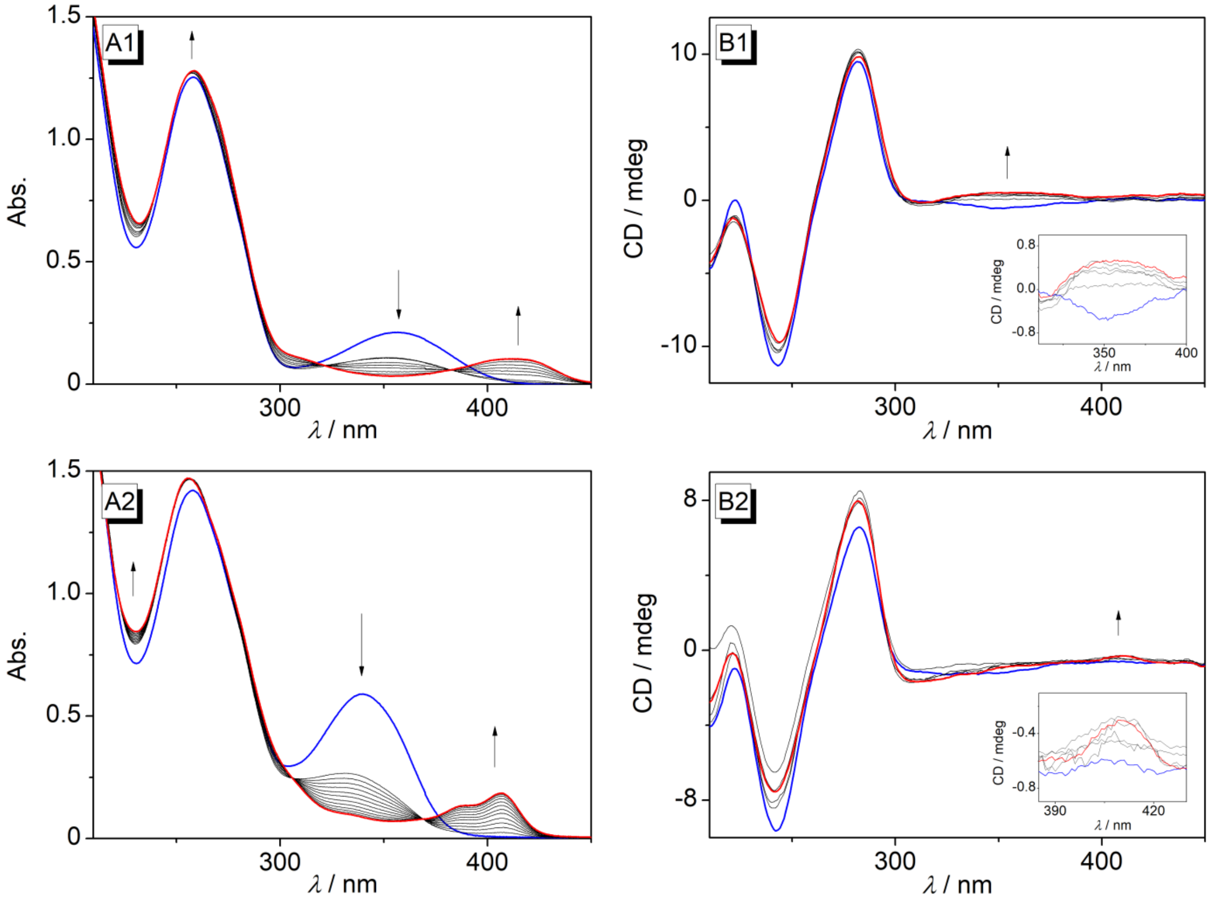

Figure S7: Changes of the absorption (A) and CD (B) spectra during the irradiation of **2d** (1) and **2g** (c = 20 µM) (2) in the presence of ct DNA (c_{DNA} = 0.1 mM) in Na phosphate buffer (c_{Na+} = 16 mM). A1: c = 20 µM, 10 min; B1: c = 40 µM, 5 min; A2: 2 min; B2: 5 min. Blue: spectrum of the starting material before irradiation; red: spectrum at the end of the irradiation.

5. Photometric and fluorimetric DNA titrations

Preparation of buffer solutions

The buffer solutions were prepared from biochemistry-grade chemicals (Fluka BioChemika Ultra) and E-Pure® water (18 MΩ cm), filtered through a PVDF membrane filter (pore size 0.45 µm) prior to use and kept at 4 °C. The following concentrations were used:

Na phosphate buffer: $c(Na_2HPO_4)$ = 5.8 mM, $c(NaH_2PO_4)$ = 4.2 mM; a pH value of 7.0 was adjusted with aq. HCl-solution (c = 1.0 M). Final salt concentration: $c(Na^+)$ = 38 mM.

BPE buffer: $c(Na_2HPO_4)$ = 6.0 mM, $c(NaH_2PO_4)$ = 2.0 mM, $c(Na_2EDTA)$ = 1.0 mM; a pH value of 7.0 was adjusted with aq. HCl-solution (c = 1.0 M).

HEPES buffer: c(HEPES) = 1.0 M; a pH value of 7.5 was adjusted with aq. NaOH-solution (c = 10 M).

Preparation of DNA solutions

The ct DNA (approximately 2 mg/mL) was dissolved in Na phosphate buffer, stored at 4 °C for at least 24 h and filtered through a membrane filter before use (pore size 0.45 µM; Carl Roth GmbH, Karlsruhe). The concentration (in base pairs, bp) was determined photometrically (λ_{max} = 260 nm, ε = 12824 cm^{-1}M^{-1}).

Preparation of ligand solutions

Starting from the stock solutions, a solution of the ligand (c = 20 µM) was prepared in Na phosphate buffer. The sample volume was V_{sample} = 1500 µL for the DNA titrations. The ligand was added in the same concentration to the DNA solution to prevent a dilution effect. After every titration step, the solution was equilibrated and an absorption or an emission spectrum was recorded.

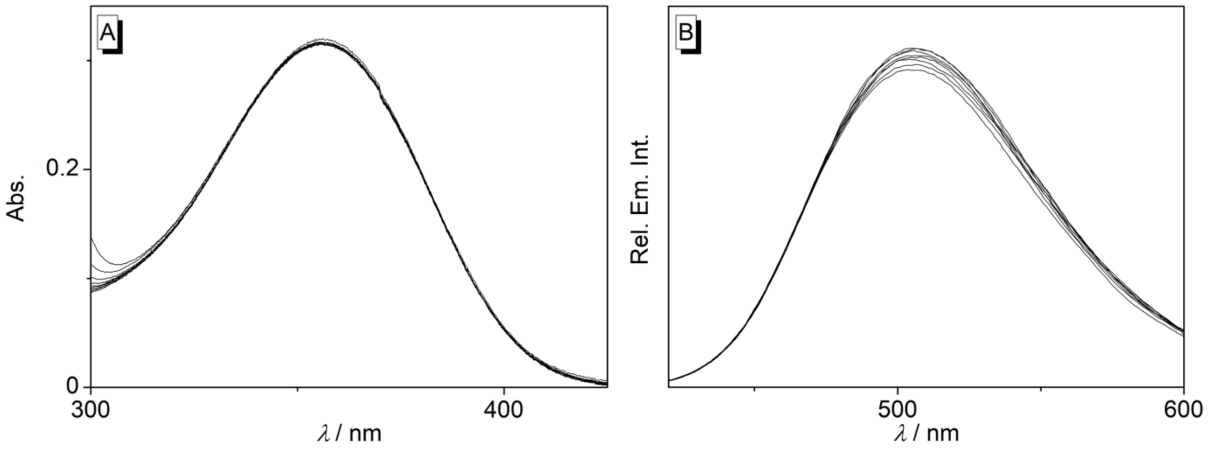

Figure S8: Photometric (A) ($c_{DNA}/c_{\mathbf{2c}}$ = 0–6.4) and fluorimetric (B) ($c_{DNA}/c_{\mathbf{2c}}$ = 0–12.5) titration of ct DNA to **2d** ($c_{\mathbf{2c}}$ = 20 µM) in Na phosphate buffer (pH 7.0, T = 20 °C, c_{Na+} = 16 mM), λ_{ex} = 355 nm.

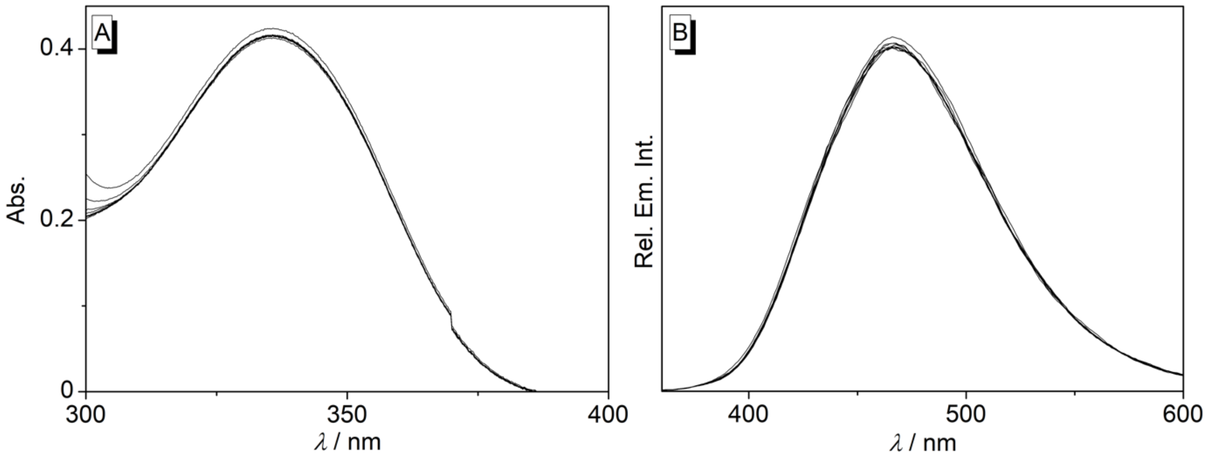

Figure S9: Photometric (A) ($c_{DNA}/c_{\mathbf{2e}}$ = 0–6.3) and fluorimetric (B) ($c_{DNA}/c_{\mathbf{2e}}$ = 0–6.3) titration of ct DNA to **2e** ($c_{\mathbf{2e}}$ = 20 µM) in Na phosphate buffer (pH 7.0, T = 20 °C, c_{Na+} = 16 mM), λ_{ex} = 335 nm.

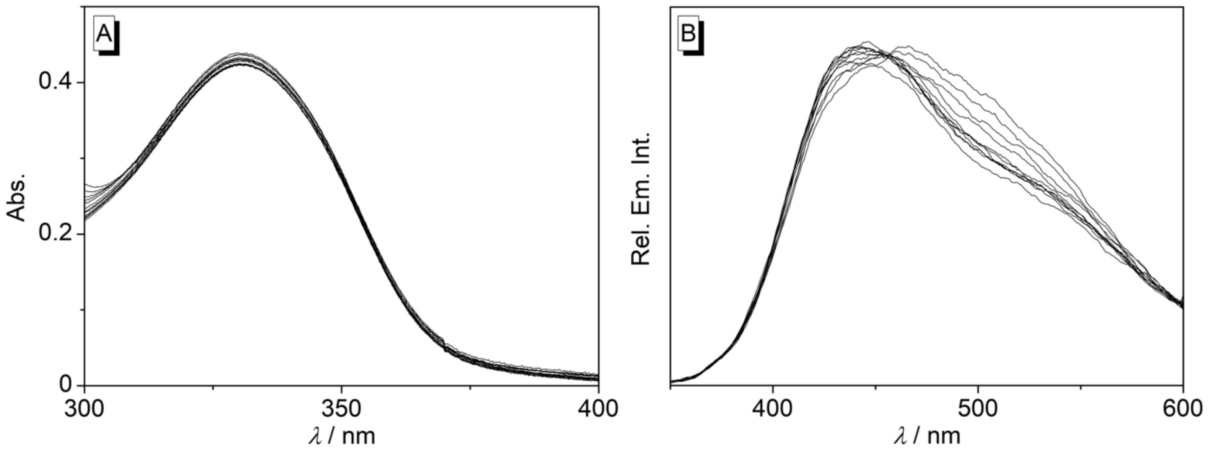

Figure S10: Photometric (A) ($c_{DNA}/c_{\mathbf{2d}}$ = 0–5.2) and fluorimetric (B) ($c_{DNA}/c_{\mathbf{2d}}$ = 0–9.5) titration of ct DNA to **2f** ($c_{\mathbf{2d}}$ = 20 µM) in Na phosphate buffer (pH 7.0, T = 20 °C, c_{Na+} = 16 mM), λ_{ex} = 330 nm.

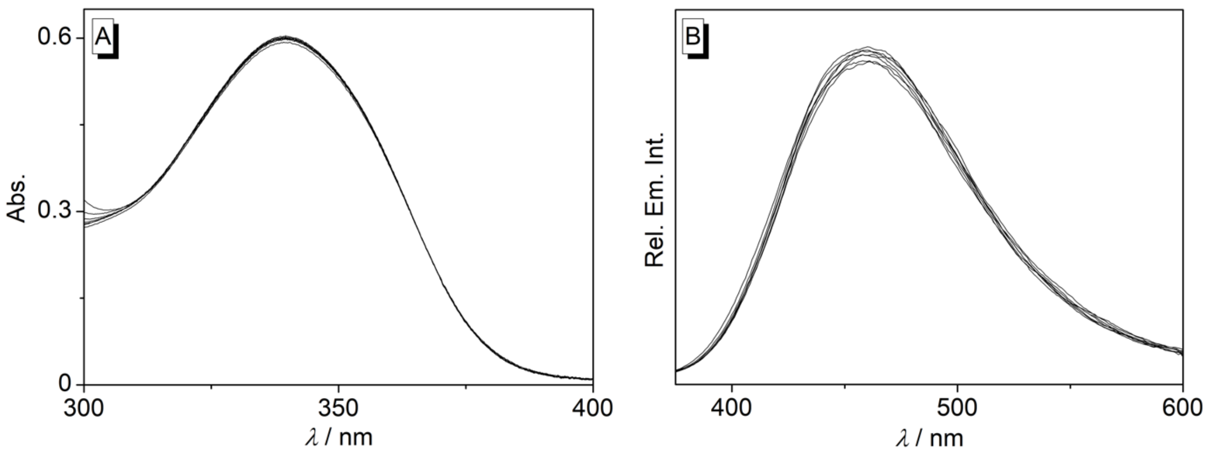

Figure S11: Photometric (A) ($c_{DNA}/c_{\mathbf{2g}}$ = 0–5.9) and fluorimetric (B) ($c_{DNA}/c_{\mathbf{2g}}$ = 0–12.0) titration of ct DNA to **2g** ($c_{\mathbf{2g}}$ = 20 µM) in Na phosphate buffer (pH 7.0, T = 20 °C, c_{Na+} = 16 mM), λ_{ex} = 340 nm.

6. CD and LD spectra

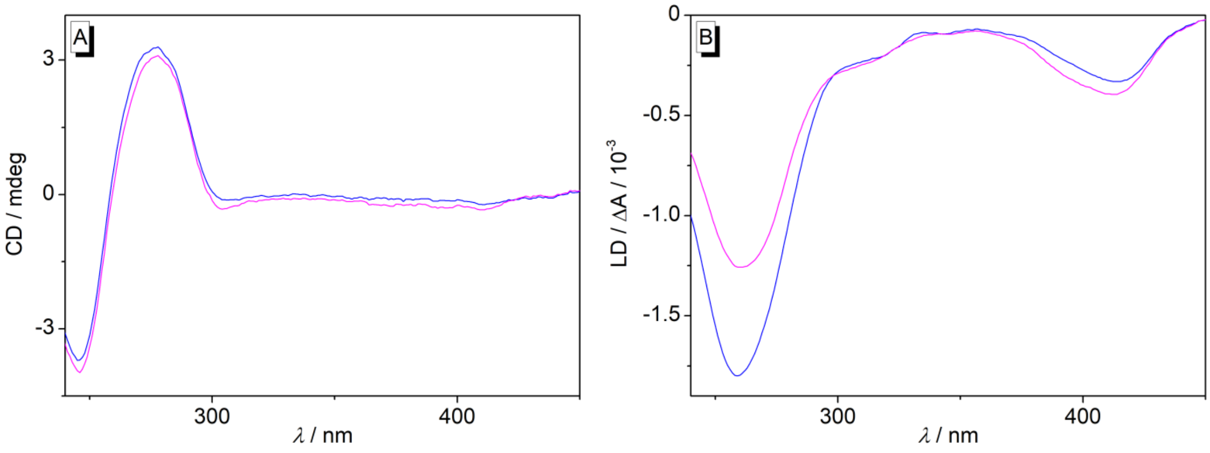

Figure S12: CD (A) and LD (B) spectra of **3c** and ct DNA (c_{DNA} = 20 μM) in Na phosphate buffer (pH 7.0, T = 20 °C, c_{Na+} = 16 mM) at *LDR* = 1.0 (blue) and 2.0 (magenta).

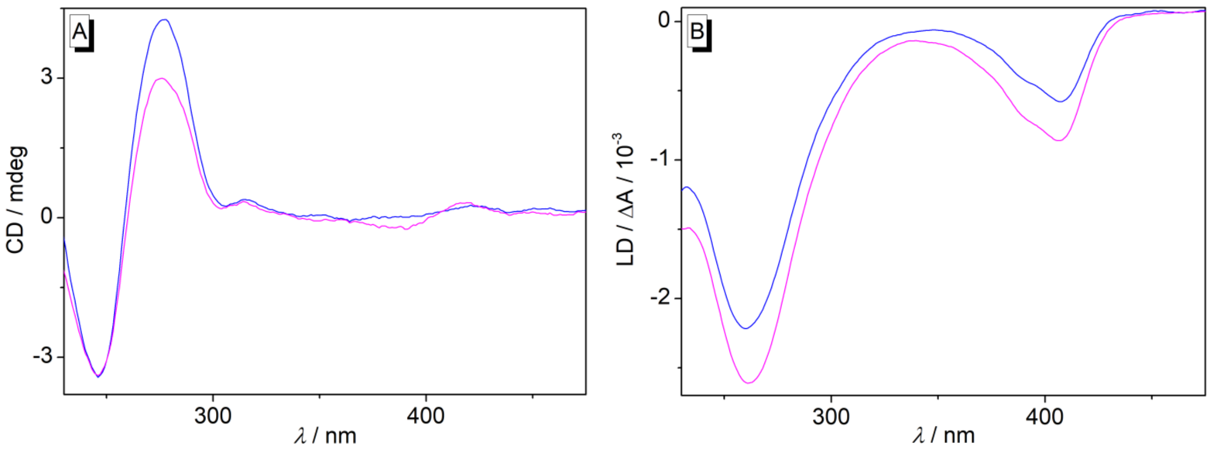

Figure S13: CD (A) and LD (B) spectra of **3e** and ct DNA (c_{DNA} = 20 μM) in Na phosphate buffer (pH 7.0, T = 20 °C, c_{Na+} = 16 mM) at *LDR* = 1.0 (blue) and 2.0 (magenta).

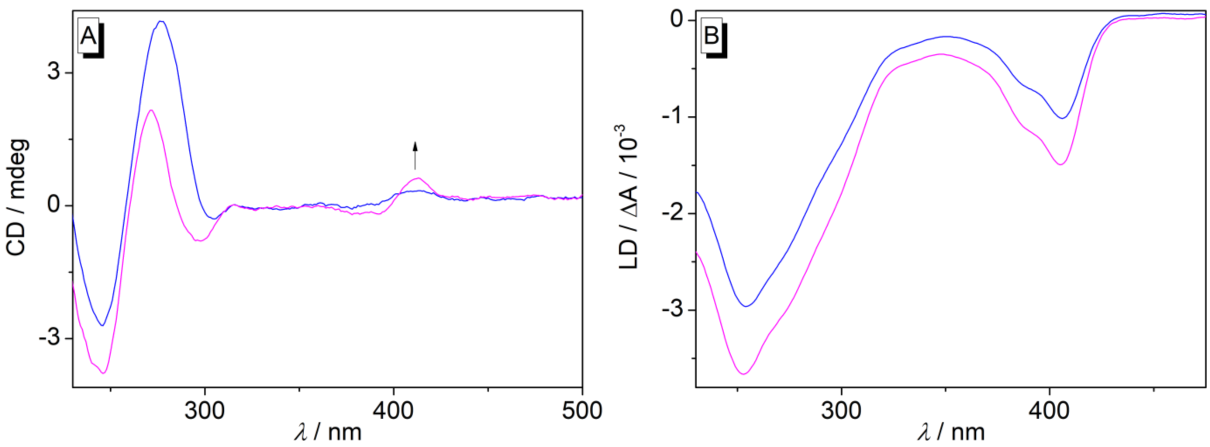

Figure S14: CD (A) and LD (B) spectra of **3g** and ct DNA (c_{DNA} = 20 μM) in Na phosphate buffer (pH 7.0, T = 20 °C, c_{Na+} = 16 mM) at *LDR* = 1.0 (blue) and 2.0 (magenta).

The reduced linear dichroism, LD_r was determined from the wavelength-dependent LD absorption (eq. 1)

$$LD_r = LD / A_{iso} \quad \text{(eq. 1)}$$

A_{iso} is the absorbance of the sample in isotropic medium and is determined under the same conditions as the LD spectra. The LD_r depends on the orientation of the DNA (S) and on the angle α between the transition dipole moment of the ligand and the DNA helix axis (eq. 2).

$$LD_r = \frac{3}{2} \times S\,(3\cos^2\alpha - 1) \quad \text{(eq. 2)}$$

With α = 90° for the DNA bases the α value of a DNA-bound ligand is given by eq. 3.

$$\alpha_L = \arccos\sqrt{\frac{1}{3} \times \left(1 - \frac{(\mathrm{LDr})_L}{(\mathrm{LDr})_{\mathrm{DNA}}}\right)} \quad \text{(eq.3)}$$

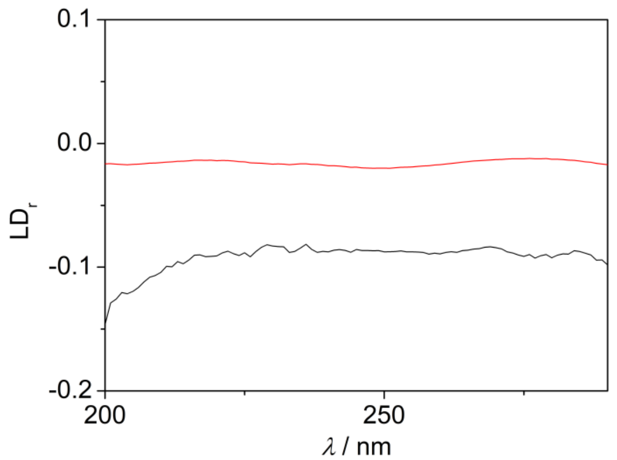

Figure S15: Reduced LD_r of a solution of **3f** and ct DNA (c_{DNA} = 20 µM) in Na phosphate buffer (pH 7.0, T = 20 °C, c_{Na+} = 16 mM) at LDR = 0 (black) and 2.0 (red).

7. Gel electrophoresis

Preparation of buffer solutions

The buffer solutions were prepared from biochemistry-grade chemicals (Fluka BioChemika Ultra) and E-Pure® water (18 MΩ cm), filtered through a PVDF membrane filter (pore size 0.45 µm) prior to use and kept at 4 °C. The following concentrations were used:

TBE buffer: c(tris(hydroxymethyl)aminomethan) = 89.0 mM, $c(H_3BO_3)$ = 89.0 mM, $c(Na_2EDTA)$ = 1.0 mM, pH 8.0.

$I_{1/2}$ buffer: $c(KH_2HPO_4)$ = 5.0 mM, 50.0 mM NaCl; a pH value of 7.4 was adjusted with aq. KOH-solution (c = 10 M).

Preparation of pBr322 DNA solution

The DNA stock solution was obtained by dilution of the commercially available plasmid DNA solution (c_{DNA} = 500 mg/L) with e-pure water to c_{DNA} = 100 mg/L. For preparation of the DNA solution used in the photocleavage experiment, fresh solutions were prepared from the

pBr322 DNA, $I_{1/2}$ buffer solution and e-pure water in a ratio of 1:2:2 (c_{DNA} = 20 mg/L), accounting for 24 µL pBr322 DNA solution, 48 µl $I_{1/2}$ buffer solution and 48 µL e-pure water for 22 pockets.

Preparation of agarose gel

TBE buffer solution (6 mL) and e-pure water (54 mL) were heated to 120 °C, agarose (0.6 g) was added and the suspension was heated to 130 °C until the agarose had dissolved. The gel was cooled down to 70 °C and ethidium bromide (c = 2.0 mg/mL, 15 µL) was added. The gel was poured into a gel chamber, where gel combs were mounted into the gel for the pocket formation. The gel was left to polymerize at room temperature within 1–2 h. The gel combs were removed and the gel was transferred to the electrophoresis chamber containing the running buffer (33 mL TBE buffer, 297 mL e-pure water) and ethidium bromide (2.0 mg/mL, 82.5 µL).

Preparation of sample solutions

The reactions were conducted in Eppendorf vials. For the reference solutions the pBr322 solution (20 mg/L, 5 µL) and e-pure water (5 µL) and for the sample solutions the pBr322 solution (20 mg/L, 5 µL) and the ligand solution with varying concentrations (5 µL) were mixed (Top-Mix) and centrifuged. To provide anaerobic or aerobic conditions, argon gas or oxygen gas was bubbled through the sample solutions with a plastic cannula. For the trapping experiments with radical scavengers, the corresponding solutions of the trapping agent in e-pure water in varying concentrations were added to the reaction mixtures and for the reactions in D_2O, the pBr322, the $I_{1/2}$ buffer and the sample solutions were lyophilized and dissolved in D_2O, which was repeated three times and the samples were then prepared as described above. The samples were irradiated for 0–15 min (λ_{exc}= 366 nm).

Procedure of gel electrophoresis

Loading buffer (2.5 µL) was added to the sample solutions and the samples were mixed and centrifuged. Each pocket of the gel was filled with the sample solution (6.5 µL) and a voltage of 80 V was applied to the electrophoresis chamber. After 1.5 h the gel electrophoresis was stopped and the gel was evaluated. For quantification, the fluorescence intensity of each band was determined, where the intensity of the superhelical DNA was multiplied by 1.66,[2] considering that ethidium bromide is intercalating less efficiently to superhelical than to open circular DNA.

A

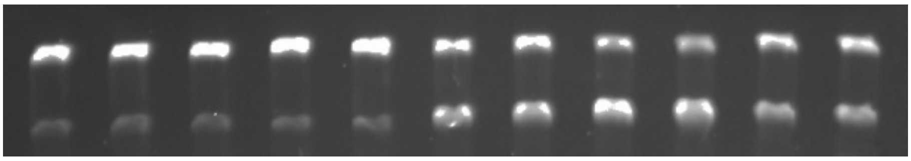

Lane	1	2	3	4	5	6	7	8	9	10	11
Irr. time / min	0	2	2	0	0	2	2	2	2	2	2
Conditions	atm	Ar	O_2	atm	atm	atm	atm	Ar	Ar	O_2	O_2
Strand cleavage / %	8	9	8	8	9	36	35	51	51	30	30

B

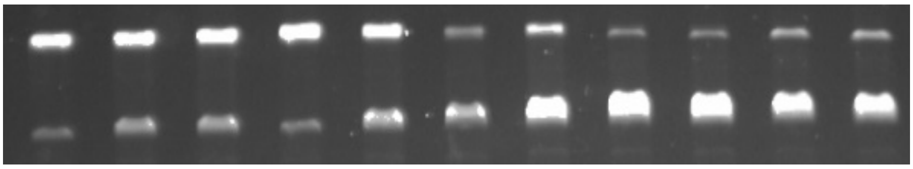

Lane	1	2	3	4	5	6	7	8	9	10	11
Irr. time / min	0	15	15	0	2	5	5	10	10	15	15
Conditions	atm	O_2	O_2	atm	O_2	O_2	O_2	O_2	O_2	O_2	O_2
Strand cleavage / %	11	22	23	12	33	67	63	85	84	73	73

Figure S16: Gel-electrophoretic analysis of photoinduced DNA-strand cleavage in the presence of **3f** under aerobic and anaerobic conditions (A) and with varying irradiation time (B). Lanes 1, 2 and 3 (A and B): control experiment without **3f**. Lane 6 (B): bands are weaker than in lane 7 because of smaller amount in the gel pocket. In all cases: $c_{\mathbf{3f}} = 2.5 \times 10^{-5}$ M, $c_{DNA} = 3.5 \times 10^{-9}$ M, $\lambda_{max} = 366$ nm.

A

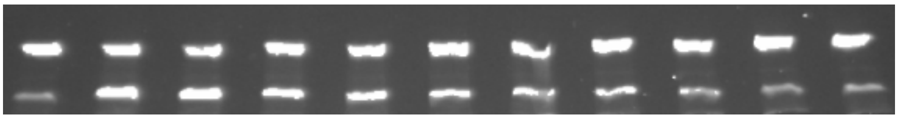

Lane	1	2	3	4	5	6	7	8	9	10	11
Additive	–	–	–	D	D	tB	tB	Pr	Pr	–	–
Strand cleavage / %	16	42	43	31	36	28	28	25	21	15	16

B

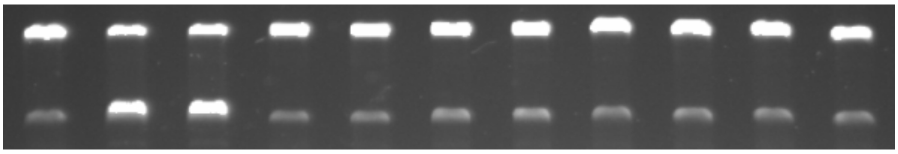

Lane	1	2	3	4	5	6	7	8	9	10	11
Additive	–	–	–	T	T	ME	ME	MA	MA	–	–
Strand cleavage / %	12	43	43	13	12	18	18	15	16	15	16

Figure S17: Gel-electrophoretic analysis of photoinduced DNA-strand cleavage in the presence of **3f** and radical scavengers DMSO (D), tBuOH (tB), and 2-PrOH (Pr) (c = 5 vol.-%) (A) and TEMPO (T) ($c = 1.3 \times 10^{-2}$ M), 2-mercaptoethanol (ME) and 2-mercapto-ethylamine hydrochloride (MA) ($c = 2.0 \times 10^{-2}$ M) (B). Lanes 1, 10 and 11 (A and B): control experiment without **3f**. Lane 1 (A and B): not irradiated. In all cases: $c_{3f} = 2.5 \times 10^{-5}$ M, $c_{DNA} = 3.5 \times 10^{-9}$ M, anaerobic conditions, irradiation time: 2 min, λ_{max} = 366 nm.

A

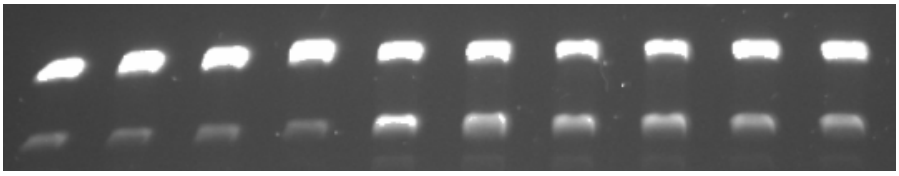

Lane	1	2	3	4	5	6	7	8	9	10
Additive/Solvent	–	–	–	–	–	–	in D_2O	in D_2O	NaN_3	NaN_3
Strand cleavage / %	8	6	7	7	26	23	22	24	18	17

B

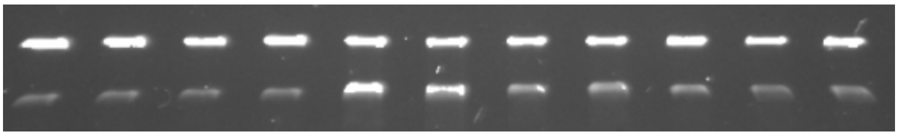

Lane	1	2	3	4	5	6	7	8	9	10	11
Additive	–	–	–	–	–	–	NaN_3	NaN_3	NaN_3	NaN_3	NaN_3
Strand cleavage / %	11	12	13	13	39	36	24	26	18	17	17

Figure S18: Gel-electrophoretic analysis of photoinduced DNA strand cleavage in the presence of **3f** depending on the $^1O_2$-radical probe (A) and on the concentration of the $^1O_2$ scavenger (B). Lanes 1, 2, 3 and 4 (A and B): control experiment without **3f**. Lanes 1 and 2 (A and B): not irradiated. Lanes 9 and 10 (A) and lanes 7 and 8 (B): $c_{\mathbf{3f}} = 1.3 \times 10^{-5}$ M. Lanes 9, 10 and 11 (B): $c_{\mathbf{3f}} = 1.3 \times 10^{-5}$ M, $c_{azide} = 2.5 \times 10^{-4}$ M. In all cases: $c_{\mathbf{3f}} = 2.5 \times 10^{-5}$ M, $c_{DNA} = 3.5 \times 10^{-9}$ M, $c_{sodium\ azide} = 2.5 \times 10^{-5}$ M, aerobic conditions, irradiation time: 2 min, λ_{max} = 366 nm.

8. NMR spectra

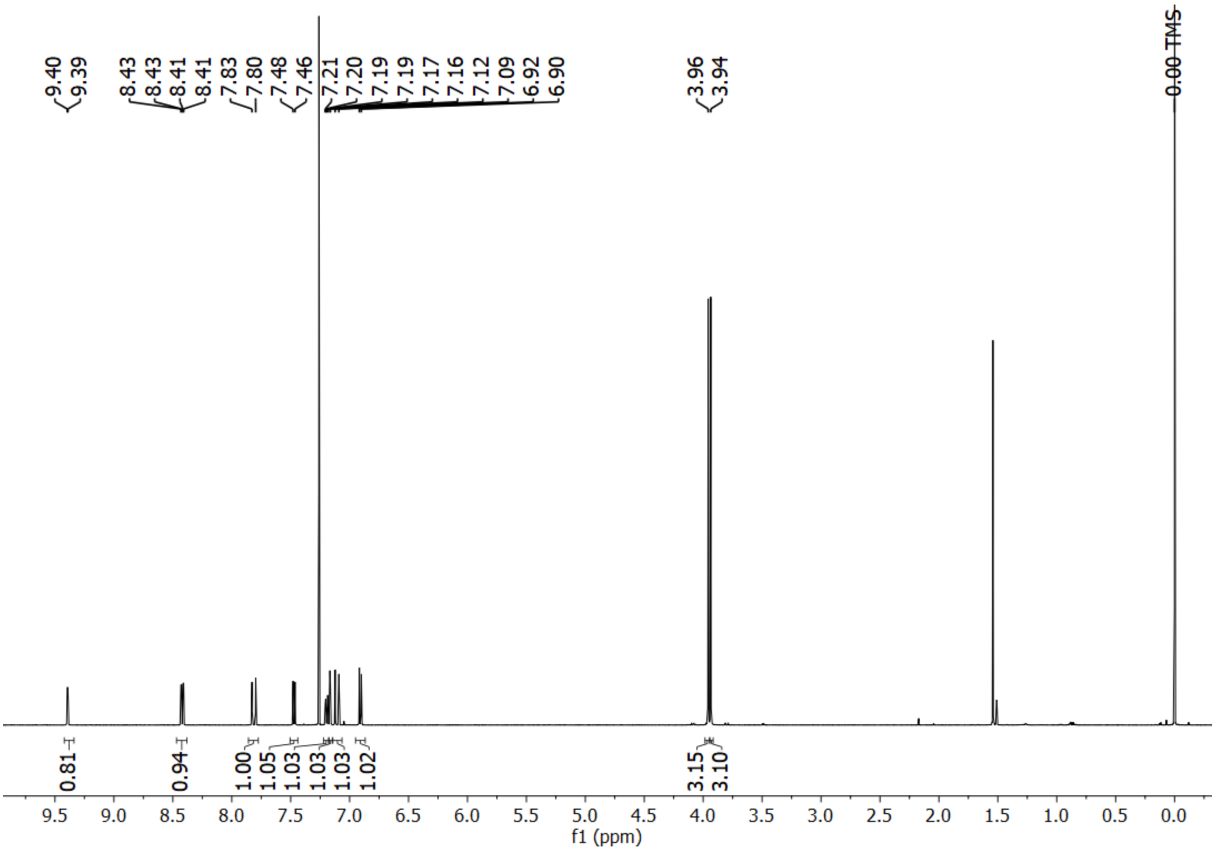

Figure S19: ^{1}H NMR spectrum (500 MHz) of **2a** in $CDCl_3$.

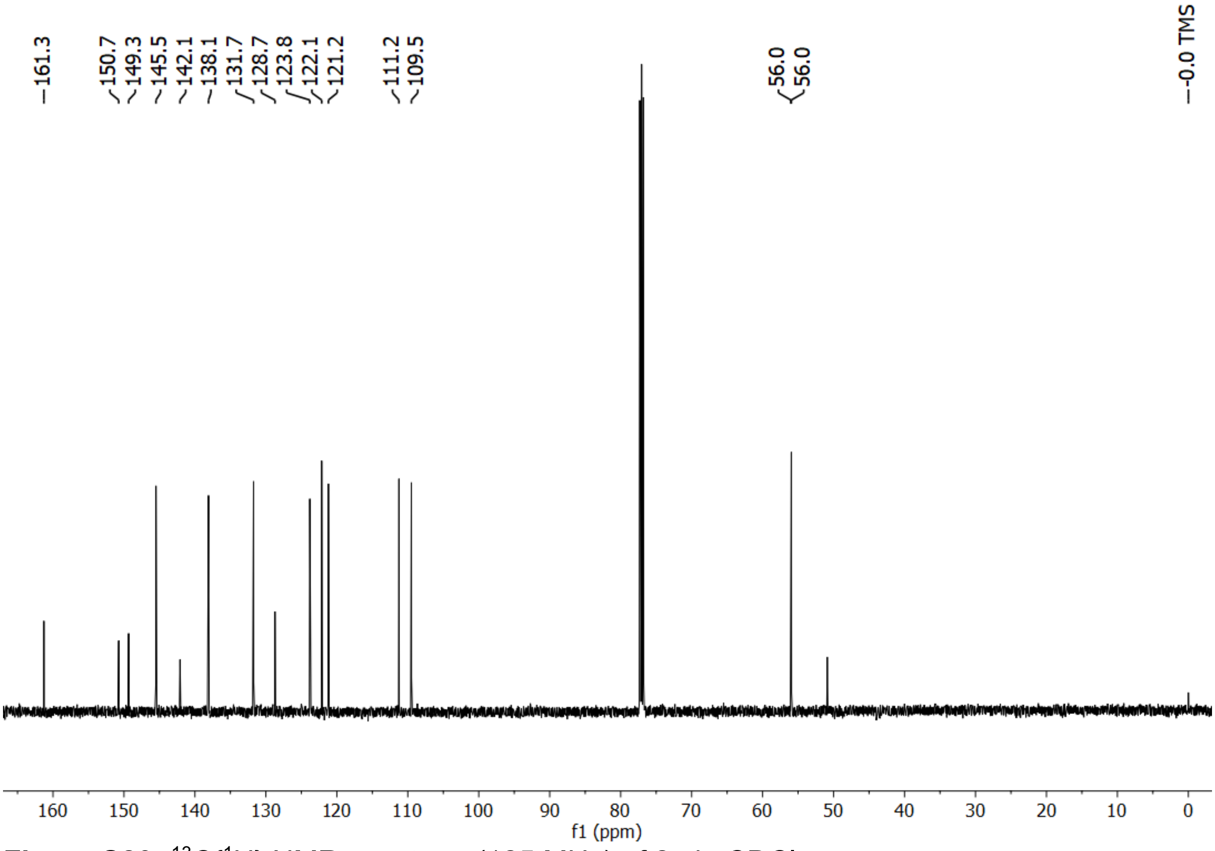

Figure S20: ^{13}C{^{1}H} NMR spectrum (125 MHz) of **2a** in $CDCl_3$.

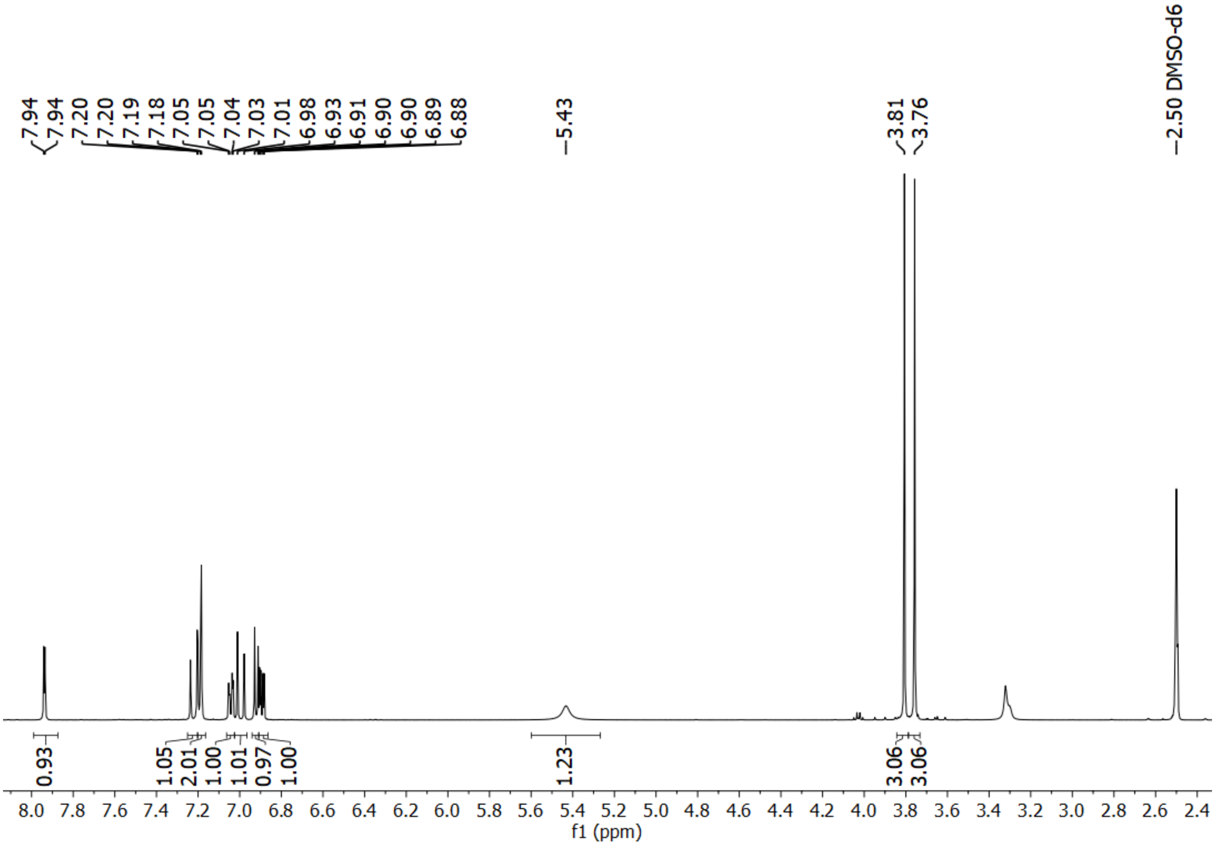

Figure S21: ^{1}H NMR spectrum (500 MHz) of **2b** in DMSO-d_6.

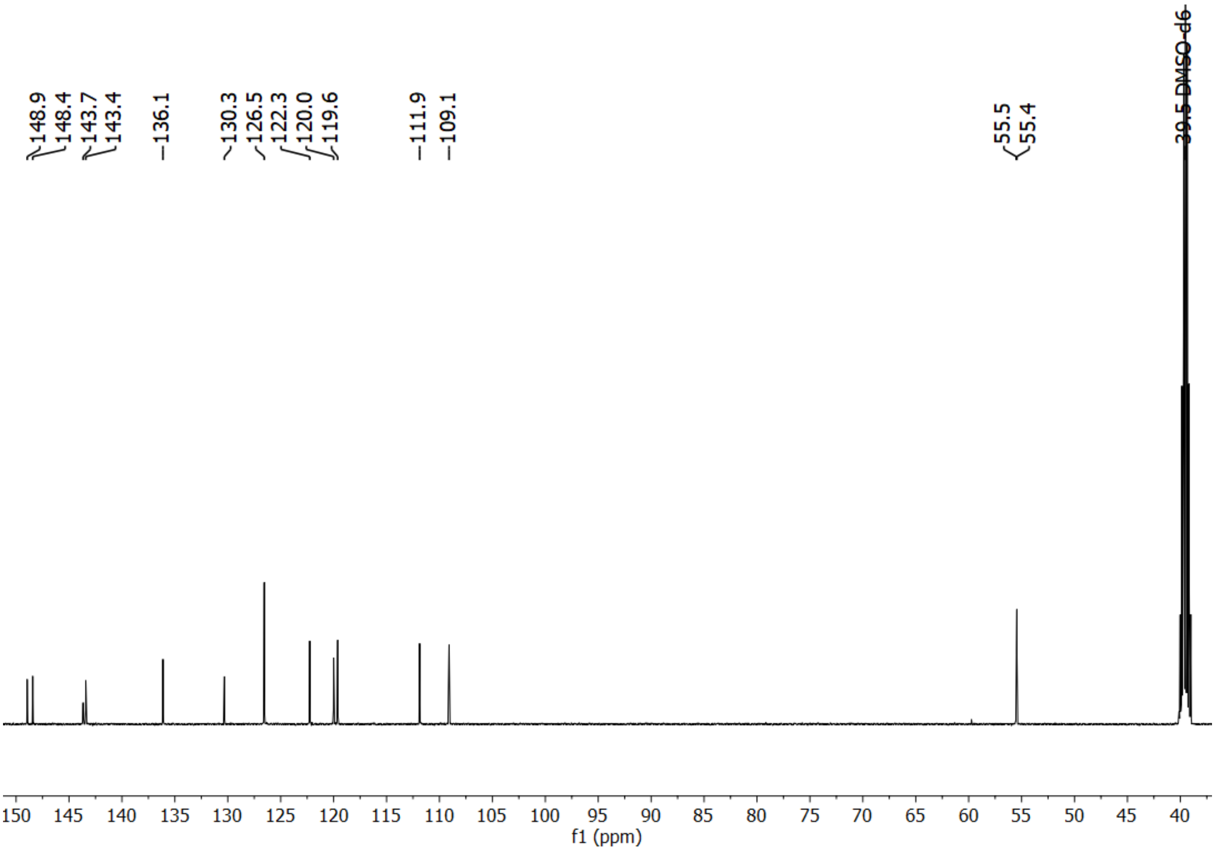

Figure S22: ^{13}C{^{1}H} NMR spectrum (125 MHz) of **2b** in DMSO-d_6.

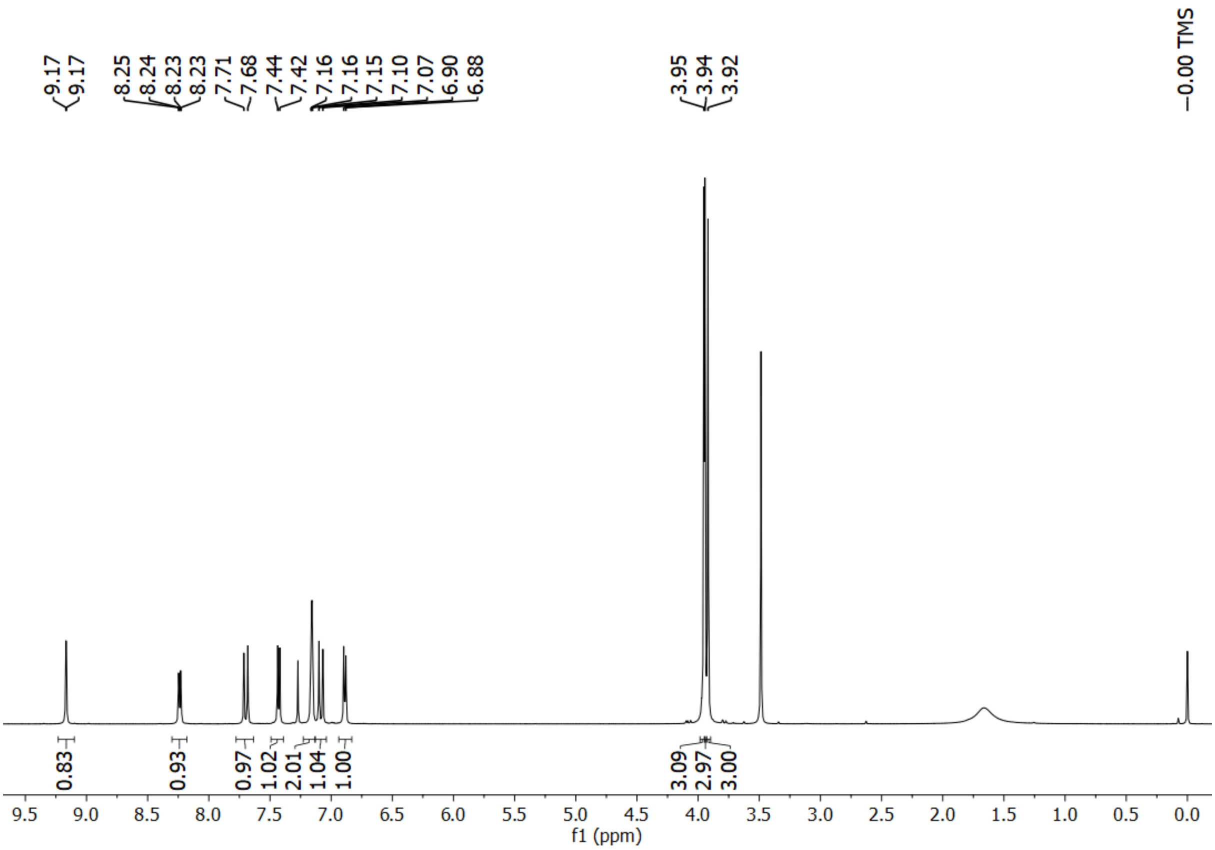

Figure S23: ^{1}H NMR spectrum (500 MHz) of **2c** in $CDCl_3$.

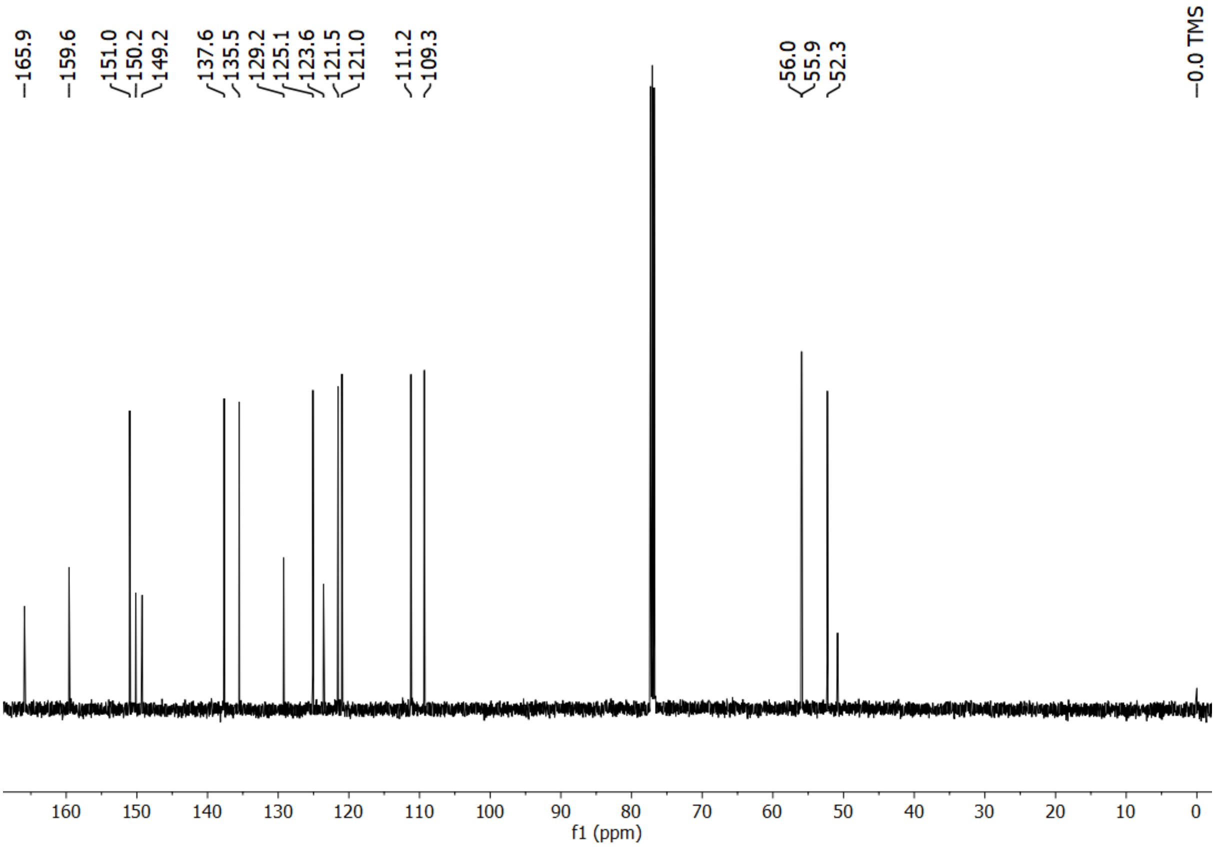

Figure S24: $^{13}C\{^{1}H\}$ NMR spectrum (125 MHz) of **2c** in $CDCl_3$.

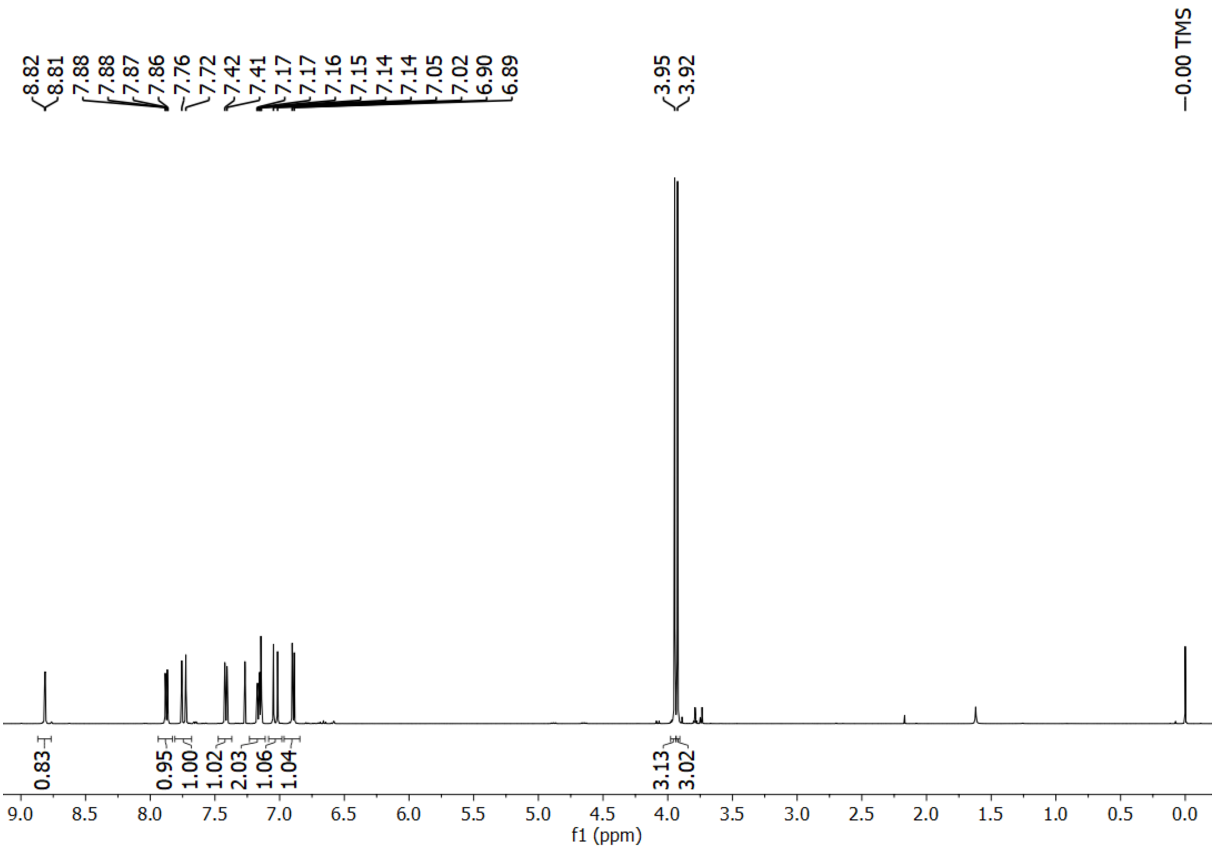

Figure S25: ^{1}H NMR spectrum (500 MHz) of **2d** in $CDCl_3$.

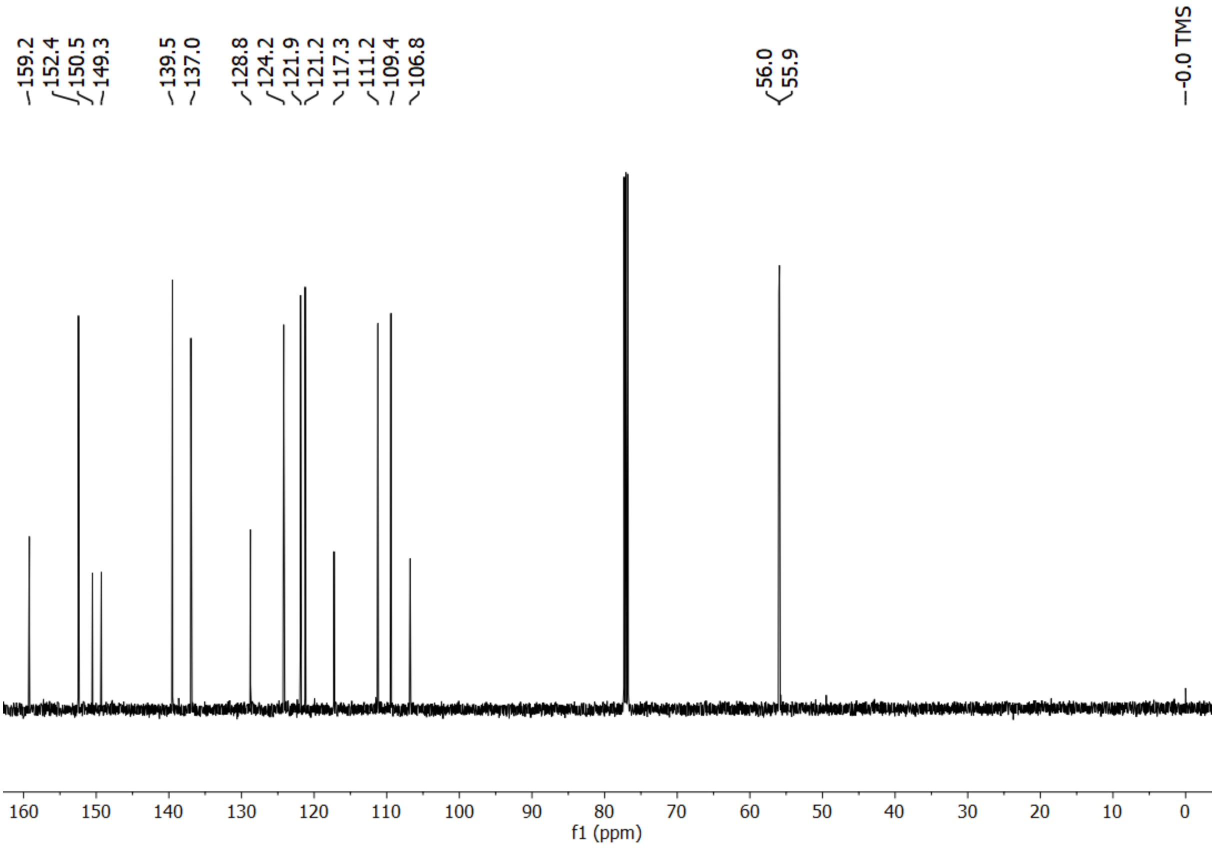

Figure S26: $^{13}C\{^{1}H\}$ NMR spectrum (125 MHz) of **2d** in $CDCl_3$.

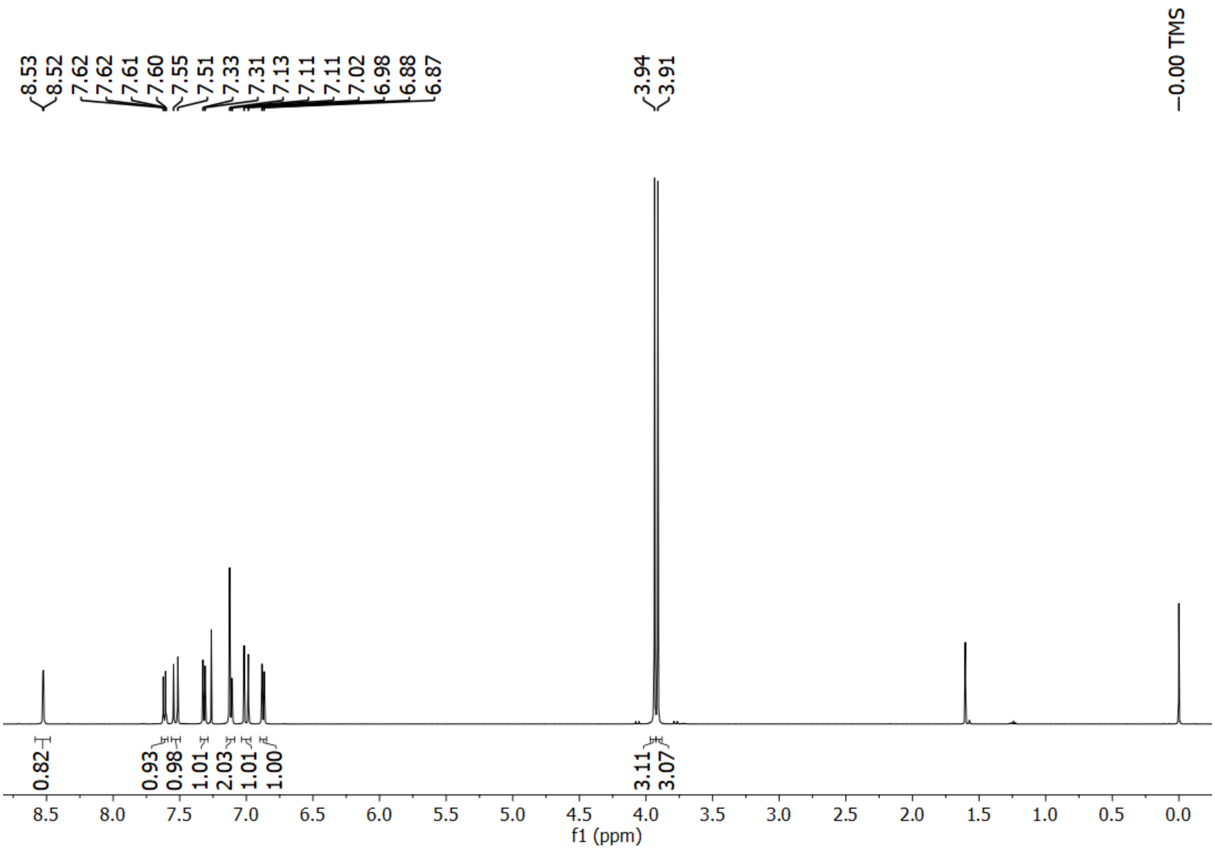

Figure S27: ^{1}H NMR spectrum (500 MHz) of **2e** in $CDCl_3$.

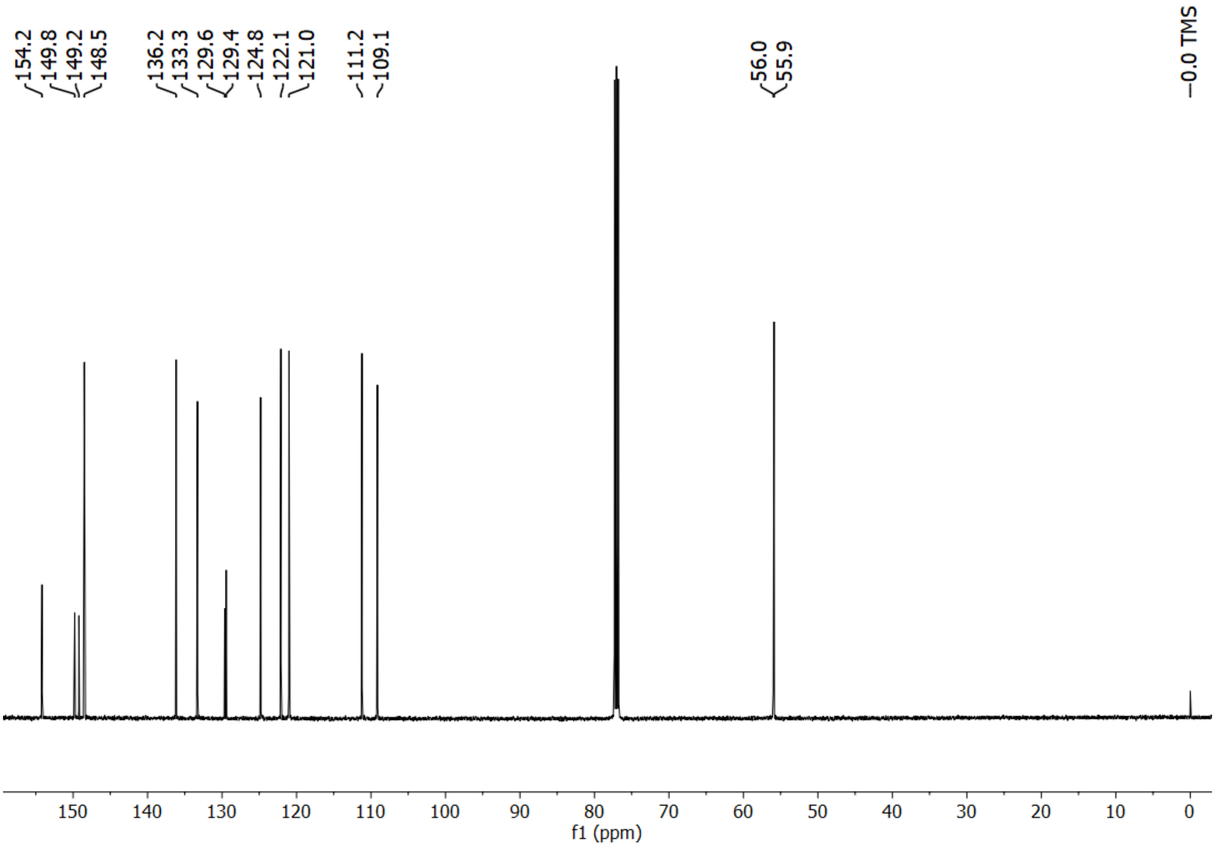

Figure S28: $^{13}C\{^{1}H\}$ NMR spectrum (125 MHz) of **2e** in $CDCl_3$.

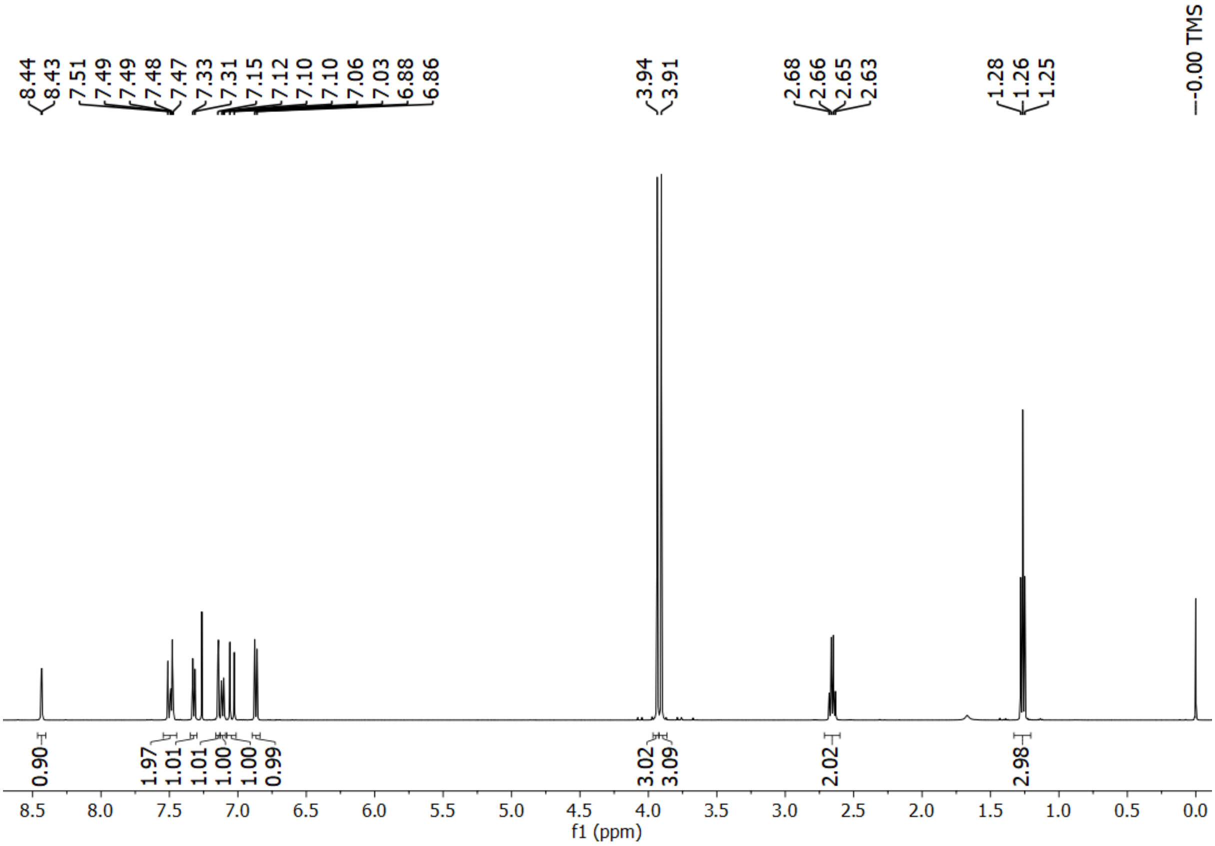

Figure S29: ^{1}H NMR spectrum (500 MHz) of **2f** in $CDCl_3$.

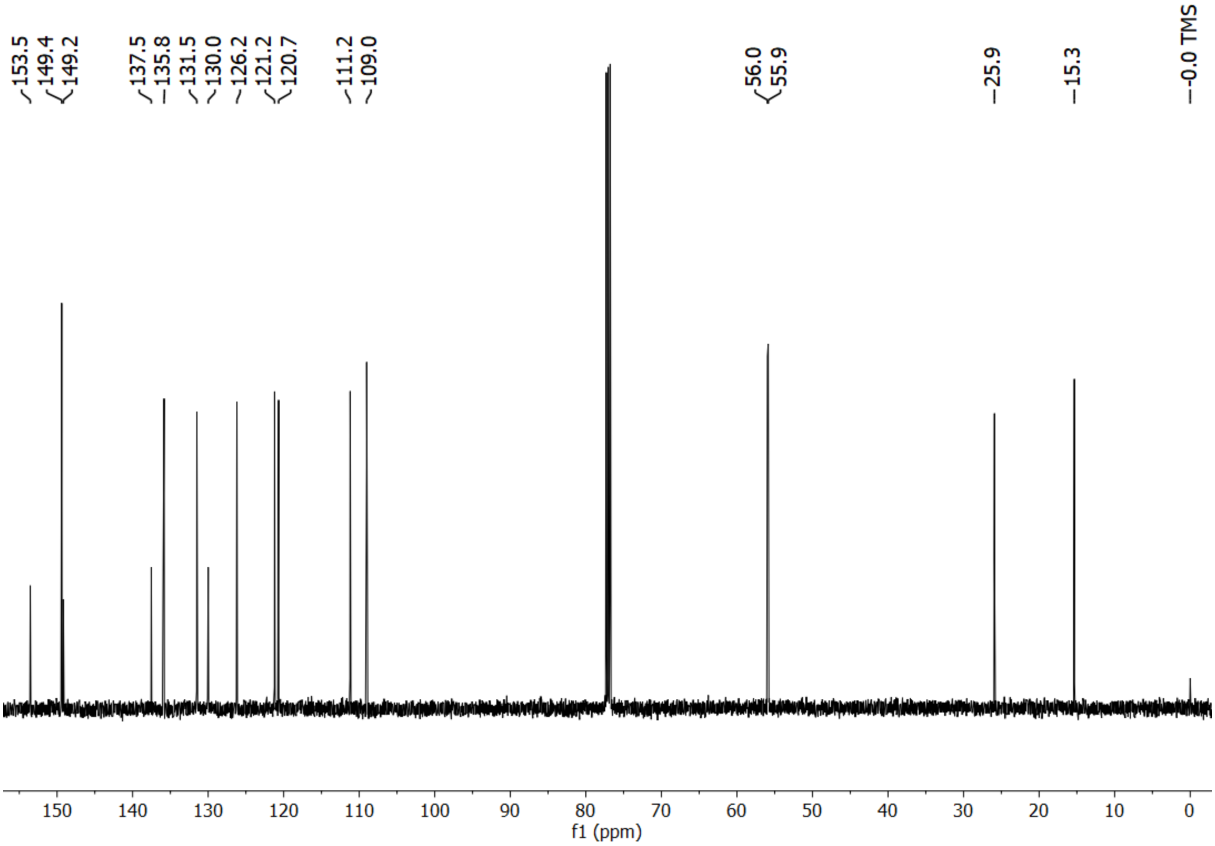

Figure S30: $^{13}C\{^{1}H\}$ NMR spectrum (125 MHz) of **2f** in $CDCl_3$.

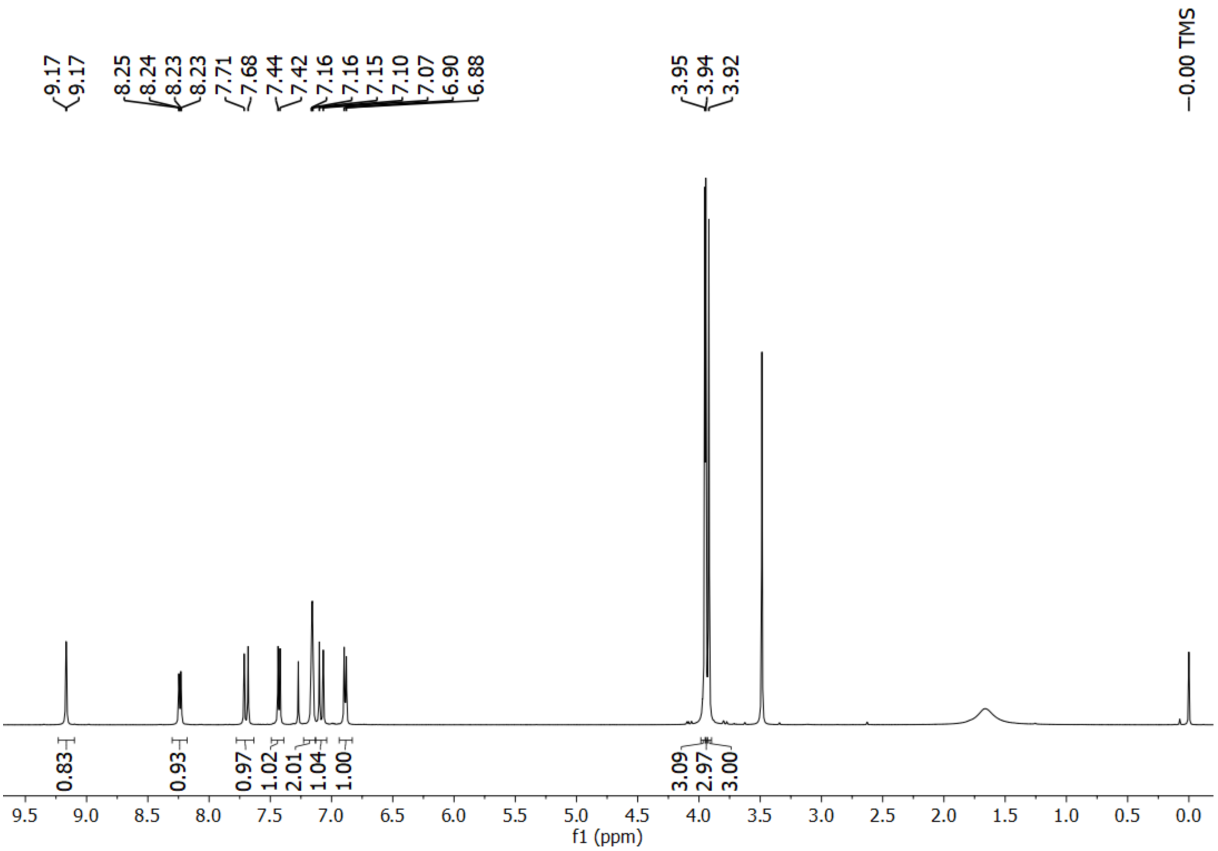

Figure S31: ^{1}H NMR spectrum (500 MHz) of **2g** in $CDCl_3$.

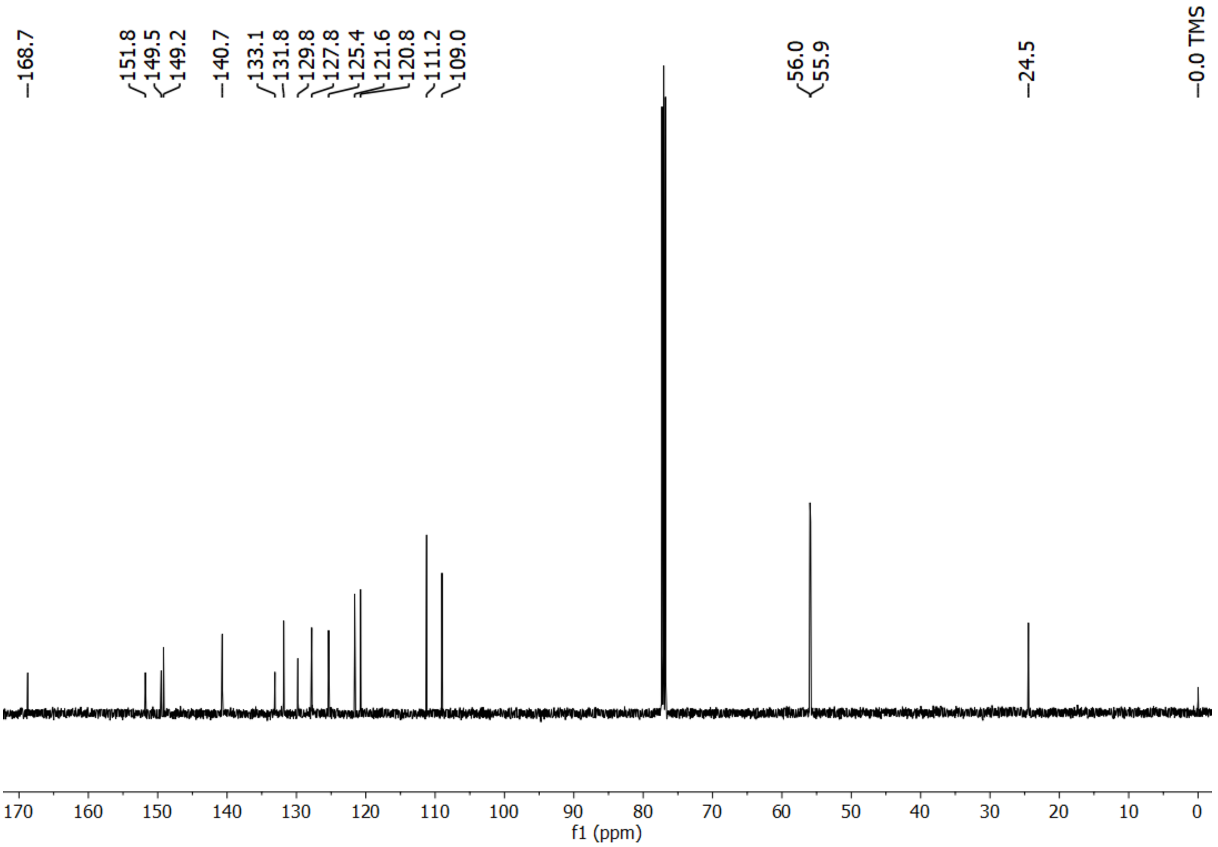

Figure S32: $^{13}C\{^{1}H\}$ NMR spectrum (125 MHz) of **2g** in $CDCl_3$.

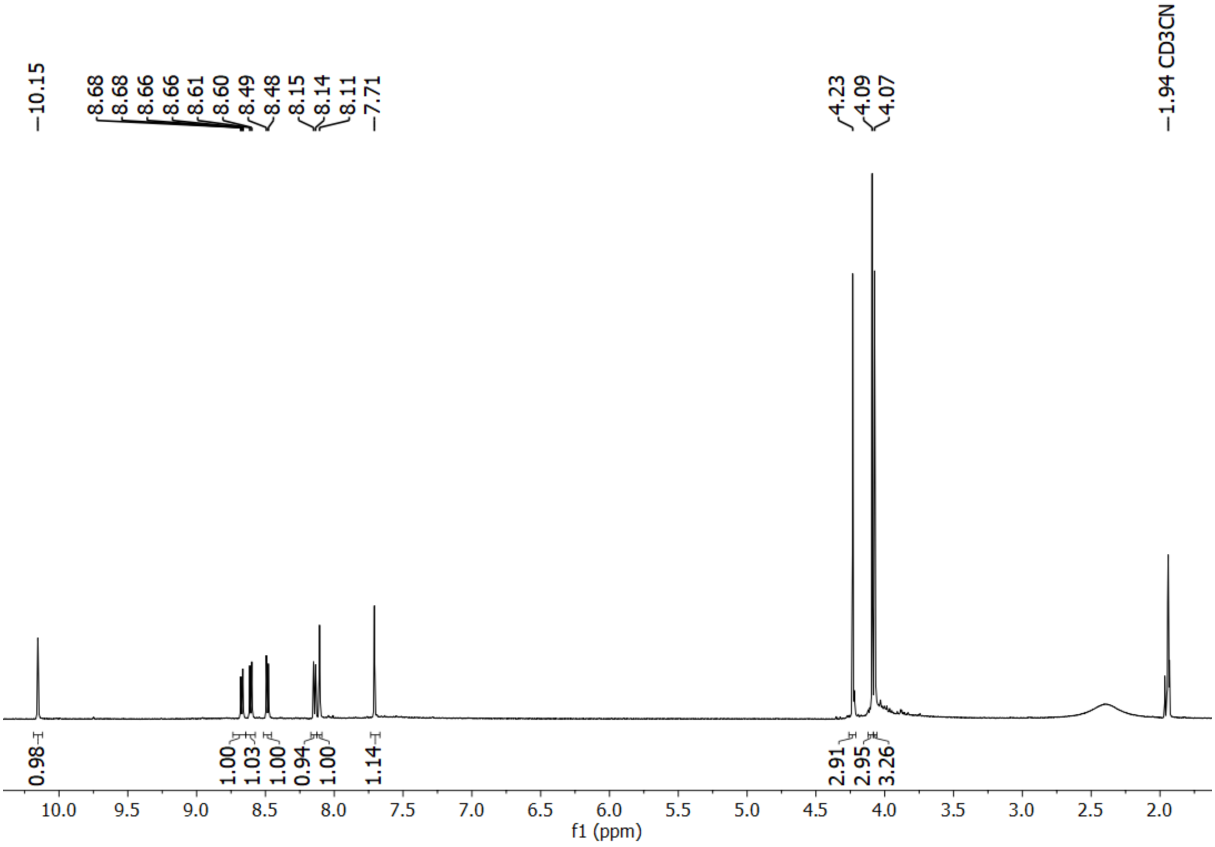

Figure S33: ^{1}H NMR spectrum (500 MHz) of **3c** in CD_3CN.

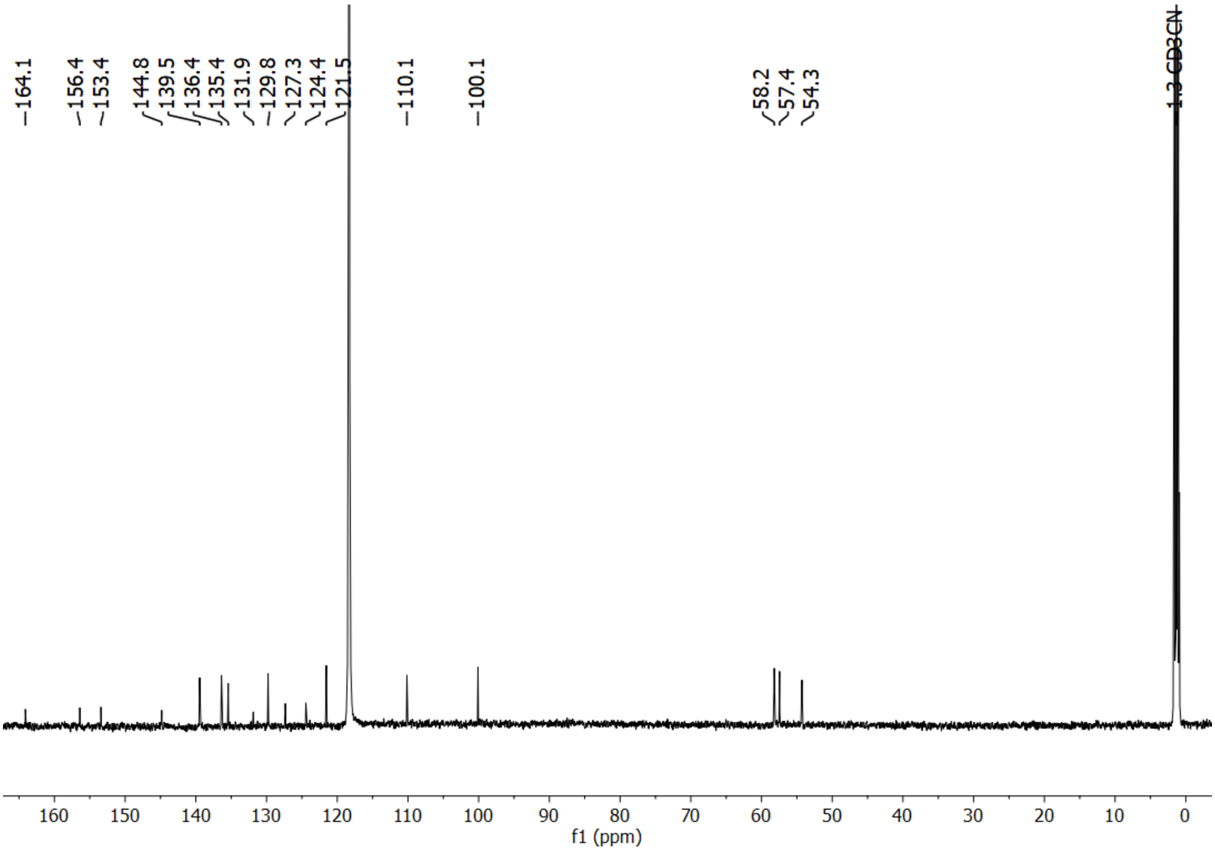

Figure S34: $^{13}C\{^{1}H\}$ NMR spectrum (125 MHz) of **3c** in CD_3CN.

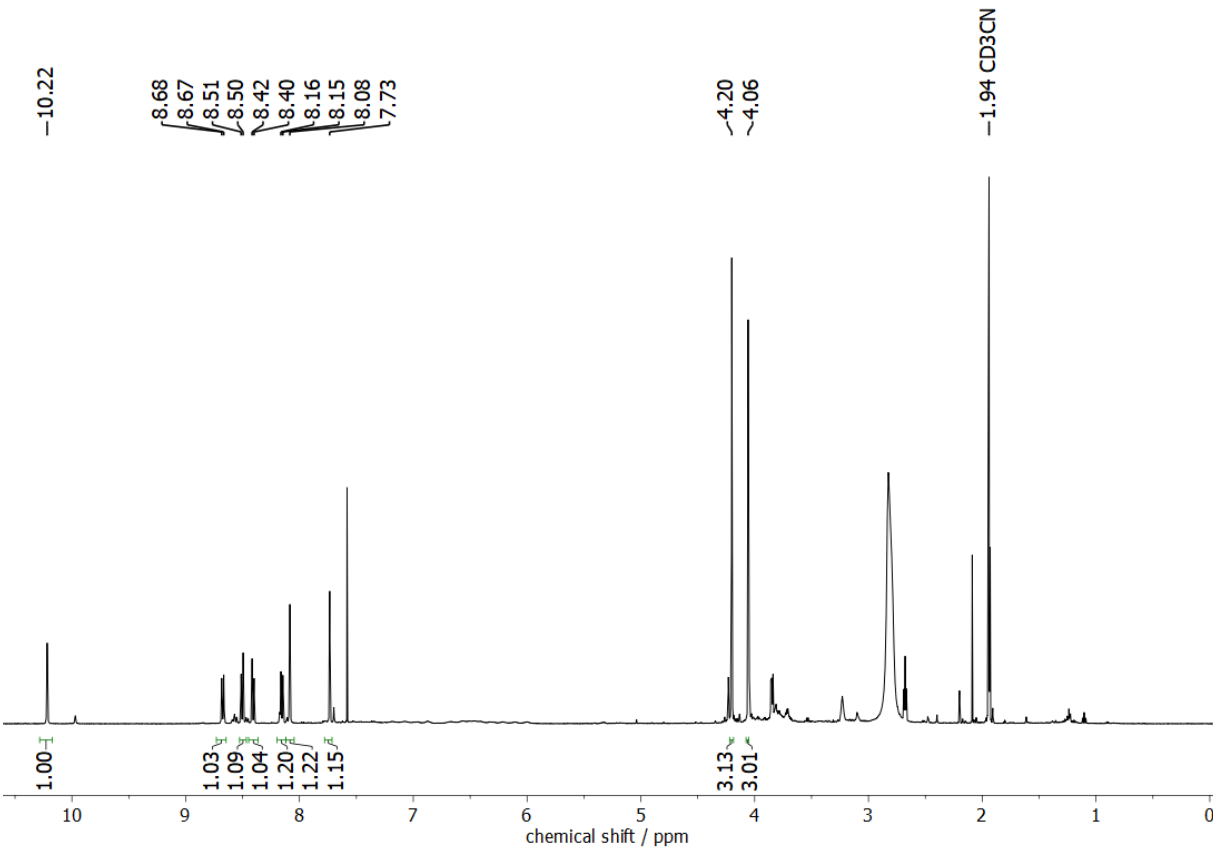

Figure S35: ^{1}H NMR spectrum (500 MHz) of **3d** in CD_3CN.

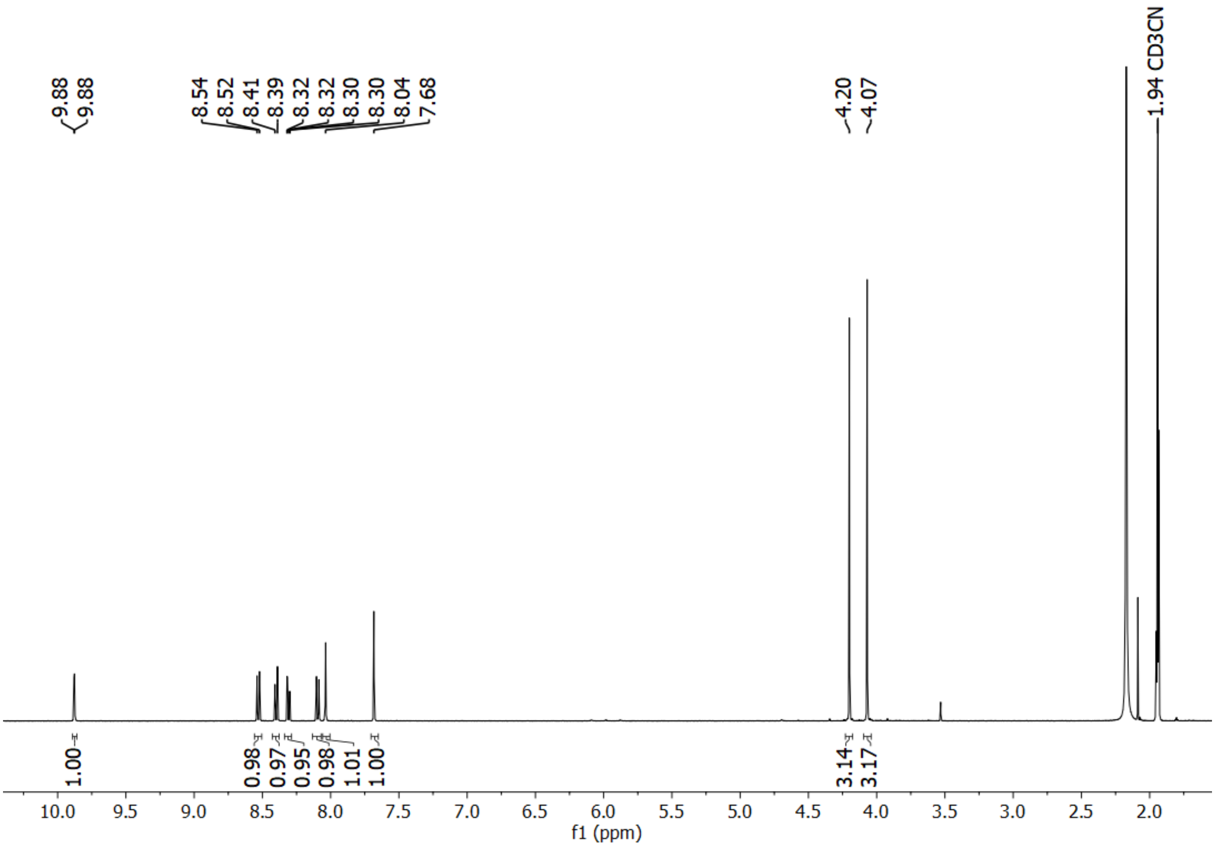

Figure S36: ^{1}H NMR spectrum (500 MHz) of **3e** in CD_3CN.

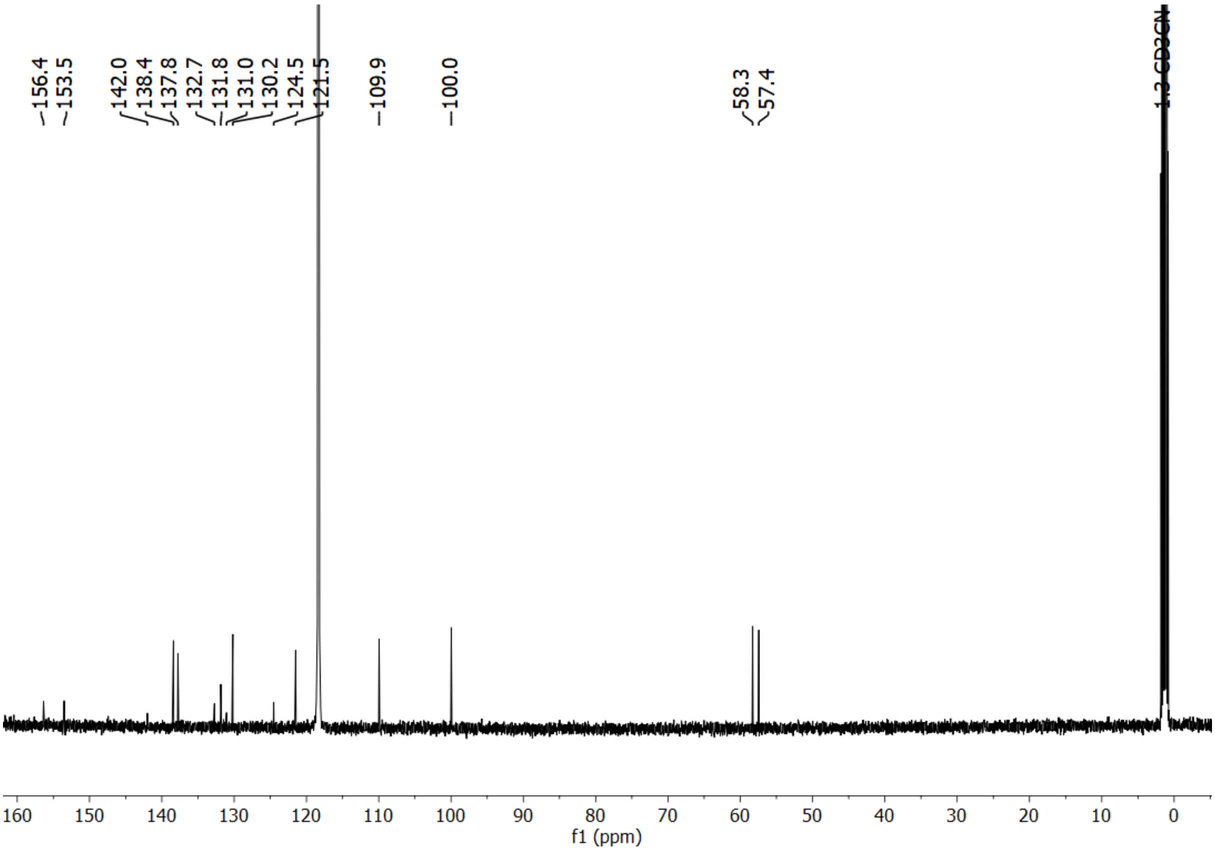

Figure S37: $^{13}C\{^{1}H\}$ NMR spectrum (125 MHz) of **3e** in CD_3CN.

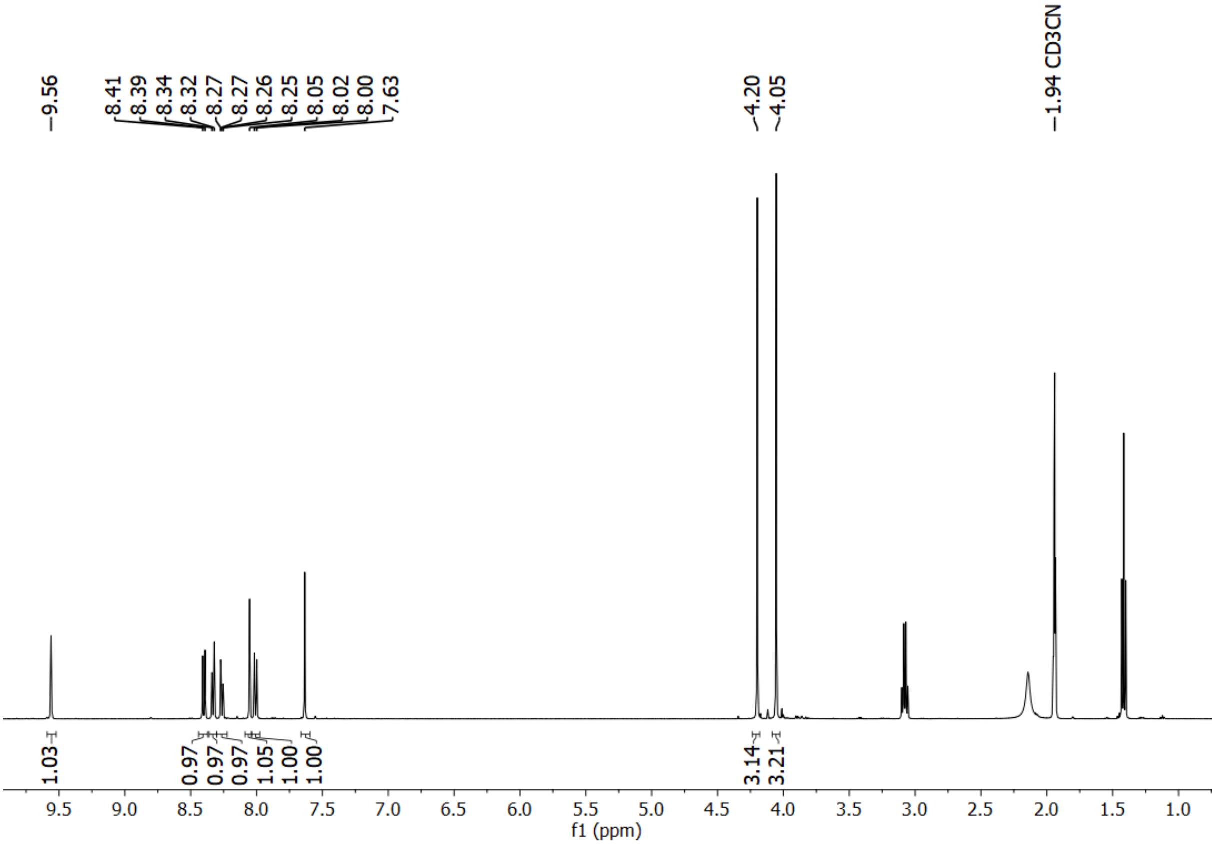

Figure S38: ^{1}H NMR spectrum (500 MHz) of **3f** in CD_3CN.

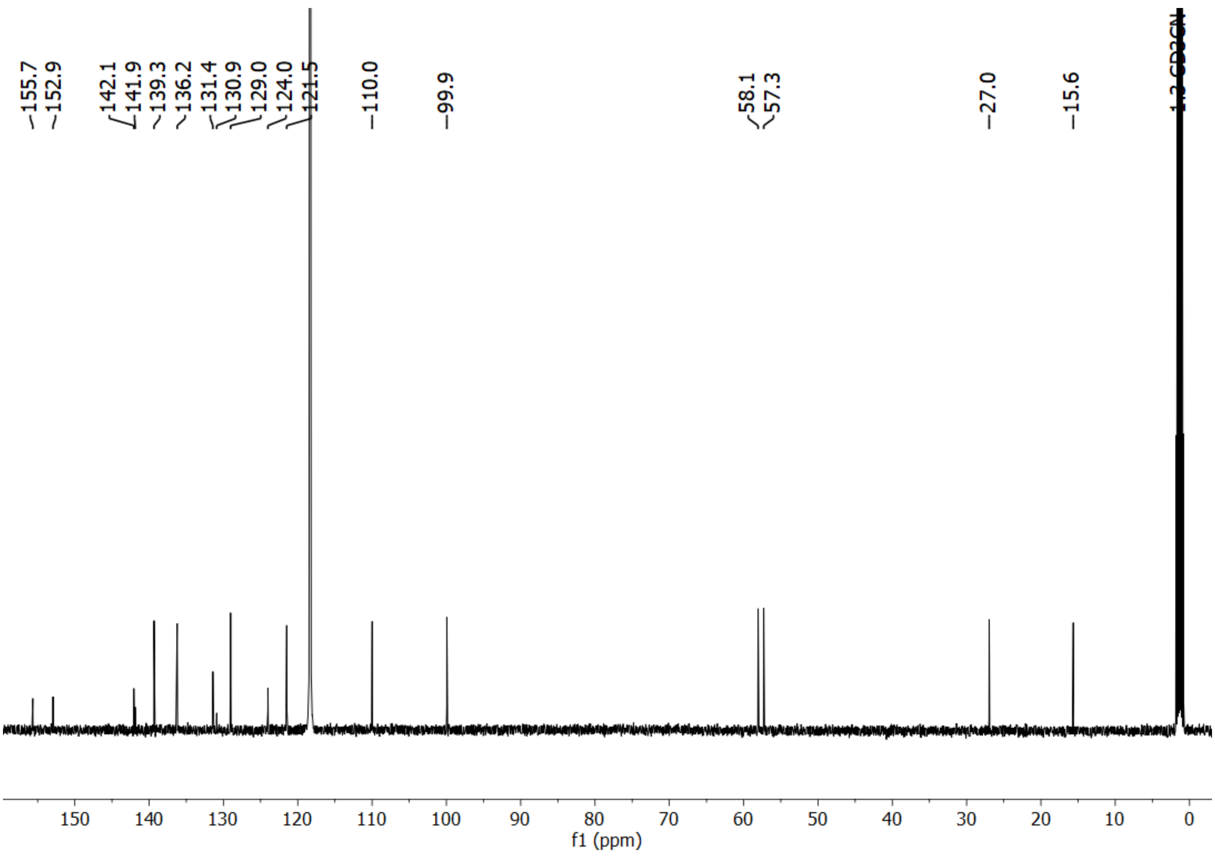

Figure S39: $^{13}C\{^{1}H\}$ NMR spectrum (125 MHz) of **3f** in CD_3CN.

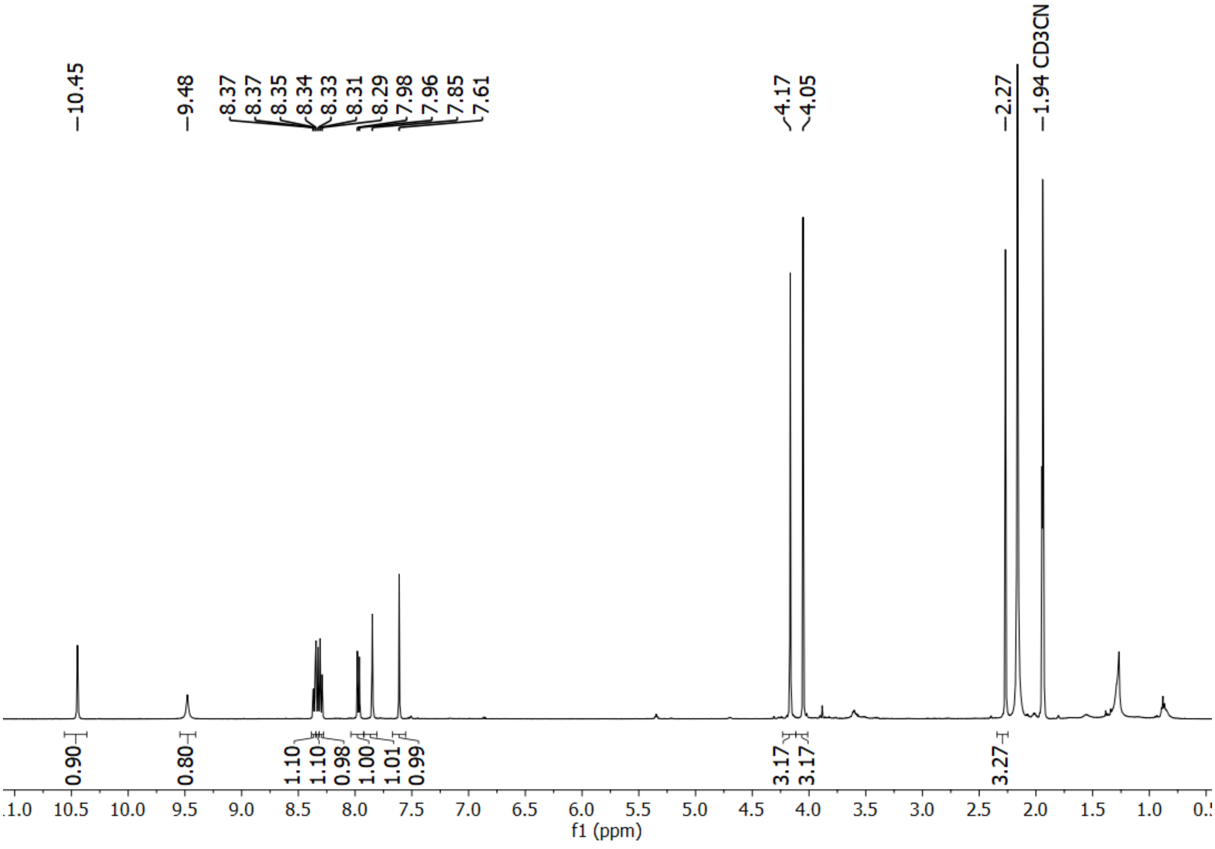

Figure S40: ^{1}H NMR spectrum (500 MHz) of **3g** in CD_3CN.

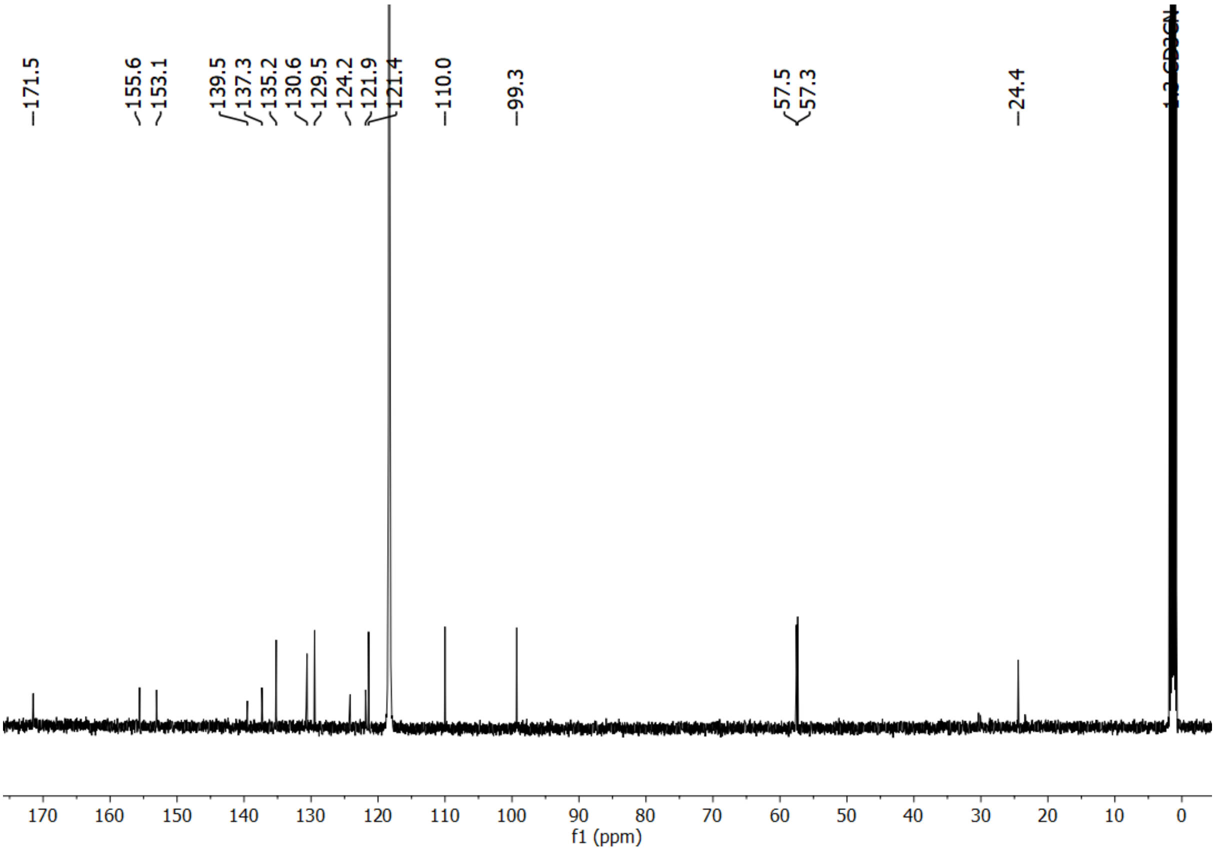

Figure S41: $^{13}C\{^1H\}$ NMR spectrum (125 MHz) of **3g** in CD_3CN.

9. References

[1] I. Cepanec, M. Litvić, H. Mikuldaš, A. Bartolinčić, V. Vinković, *Tetrahedron* **2003**, *59*, 2435.

[2] T. A. Ciulla, J. R. van Camp, E. Rosenfeld, I. E. Kochevar, *Photochem. Photobiol.* **1989**, *49*, 293.
